# Distinct Chemical Determinants are Essential for Achieving Ligands for Superior Optical Detection of Specific Amyloid‐β Deposits in Alzheimer's Disease

**DOI:** 10.1002/open.202400186

**Published:** 2024-11-07

**Authors:** Xiongyu Wu, Hamid Shirani, Ruben Vidal, Bernardino Ghetti, Martin Ingelsson, Therése Klingstedt, K. Peter R. Nilsson

**Affiliations:** ^1^ Department of Physics, Chemistry and Biology Linköping University SE-581 83 Linköping Sweden; ^2^ Department of Pathology and Laboratory Medicine Indiana University School of Medicine 46202 Indianapolis, Indiana USA; ^3^ Krembil Brain Institute University Health Network M5T 1 M8 Toronto, Ontario Canada; ^4^ Tanz Centre for Research in Neurodegenerative Diseases Departments of Medicine and Laboratory Medicine & Pathobiology University of Toronto M5T 0S8 Toronto, Ontario Canada; ^5^ Molecular Geriatrics Department of Public Health and Caring Sciences Uppsala University SE-751 85 Uppsala Sweden

**Keywords:** Ligands, Fluorescence, Alzheimer's disease, Amyloid β, Protein aggregates

## Abstract

Aggregated forms of different proteins are common hallmarks for several neurodegenerative diseases, including Alzheimer's disease, and ligands that selectively detect specific protein aggregates are vital. Herein, we investigate the molecular requirements of thiophene‐vinyl‐benzothiazole based ligands to detect a specific type of Aβ deposits found in individuals with dominantly inherited Alzheimer's disease caused by the Arctic *APP E693G* mutation. The staining of these Aβ deposits was alternated when switching the terminal heterocyclic moiety attached to the thiophene‐vinyl‐benzothiazole scaffold. The most prevalent staining was observed for ligands having a terminal 3‐methyl‐1H‐indazole moiety or a terminal 1,2‐dimethoxybenzene moiety, verifying that specific molecular interactions between these ligands and the aggregates were necessary. The synthesis of additional thiophene‐vinyl‐benzothiazole ligands aided in pinpointing additional crucial chemical determinants, such as positioning of nitrogen atoms and methyl substituents, for achieving optimal staining of Aβ aggregates. When combining the optimized thiophene‐vinyl‐benzothiazole based ligands with a conventional ligand, CN‐PiB, distinct staining patterns were observed for sporadic Alzheimer's disease versus dominantly inherited Alzheimer's disease caused by the Arctic *APP E693G* mutation. Our findings provide chemical insights for developing novel ligands that allow for a more precise assignment of Aβ deposits, and might also aid in creating novel agents for clinical imaging of distinct Aβ aggregates in AD.

## Graphical abstract

We have investigated the structural requirements of thiophene‐vinylene‐benzothiazole based ligands for binding to amyloid beta (Aβ) plaques in dominantly inherited Alzheimer's disease caused by the Arctic mutation. By synthesizing structurally diverse ligands we could show that their binding properties were determined by the nature of the terminal heterocyclic moiety and the positioning of nitrogen atoms, as well as methyl substituents, to this moiety.

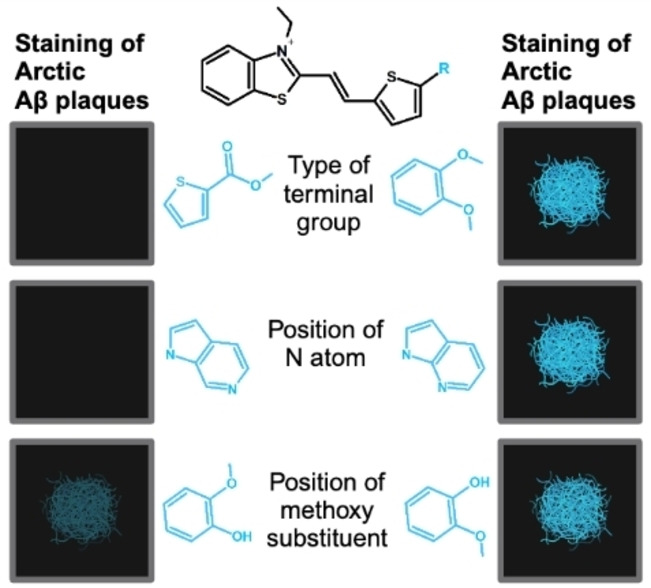

## Introduction

Protein aggregates are one of the pathological features of several proteopathic neurodegenerative diseases,[^1^, ^2^, ^3^, ^4^] including Alzheimer's disease (AD), and a diversity of synthetic fluorescent ligands that can be used for optical imaging of these proteinaceous aggregated species has been presented.[^5^, ^6^, ^7^, ^8^, ^9^, ^10^, ^11^, ^12^, ^13^, ^14^] In AD, the two main neuropathologic lesions resulting from protein aggregation, i. e., the plaques and the neurofibrillary tangles, are respectively composed of the amyloid‐β (Aβ) peptide and the tau protein. For these two proteins, ligands, which are used for positron emission tomography (PET) imaging toward a clinical diagnosis of AD, have been presented.[^15^, ^16^, ^17^, ^18^, ^19^, ^20^, ^21^, ^22^, ^23^] However, different variants of Aβ aggregates have been reported[^24^, ^25^, ^26^, ^27^, ^28^, ^29^] and lately, distinct aggregated Aβ folds have been revealed by cryo electron microscopy.[^30^, ^31^, ^32^, ^33^, ^34^, ^35^] In addition, Aβ deposits found in individuals with dominantly inherited AD (diAD) associated with the Arctic *APP* mutation (*E693G*) have shown to be negative for the conventional amyloid ligands Congo red and thioflavin S, as well as carbon‐11 labelled Pittsburgh compound‐B ([11 C]PiB), the first ligand to be used clinically as a PET tracer for imaging of Aβ deposits.[^36^, ^37^, ^38^] This PET tracer has also shown limited binding to Aβ deposits in some brain regions and to a certain type of Aβ deposits found in cases of diAD with *presenilin‐1* (*PSEN1*) mutations.[^39^, ^40^, ^41^] Hence, a variety of ligands might be required to accomplish an accurate assessment of different types of Aβ aggregates in AD.

Several thiophene‐based ligands have been utilized for fluorescence imaging of protein aggregates,[^42^, ^43^, ^44^, ^45^, ^46^, ^47^, ^48^] and ligands with distinct chemical compositions have also been employed for selective detection of Aβ deposits or tau aggregates.[^49^, ^50^, ^51^] The selectivity towards Aβ and tau aggregates could be switched by chemical reprograming of the ligand, since thiophene‐vinyl‐benzothiazole (TVB) based ligands with distinct terminal groups show different binding properties to Aβ and tau aggregates in brain tissue sections from individuals with sporadic AD (sAD).^[51]^ Recently, a combination of two thiophene‐based ligands was also employed to distinguish distinct Aβ deposits in brain tissue sections from individuals with sporadic AD or diAD associated with the *PSEN1 A431E* mutation.^[52]^ For the latter, [11 C]PiB negative Aβ deposits, denoted cotton wool plaques (CWPs), were clearly identified by a thiophene‐based ligand with a distinct chemical composition. Hence, thiophene‐based molecular scaffolds might serve as a complement to existing tracers for detection of Aβ aggregates.

Herein, we have continued to explore the possibility of generating thiophene‐based ligands that can detect Aβ deposits that are not identified by conventional ligands such as [11 C]PiB. A library of TVB based ligands was screened towards brain tissue sections from an individual with diAD caused by the Arctic *APP* mutation and ligands with distinct terminal heterocyclic motifs showed superior optical detection of Aβ deposits. Furthermore, by synthesizing additional TVB based ligands, certain chemical determinants for TVBs to detect this type of Aβ deposit could be assigned. We foresee that our findings will aid in developing ligands targeting different types of Aβ deposits, as well as facilitate the design of novel agents for clinical imaging of these pathological hallmarks in AD.

## Results and Discussion

### The Chemical Nature of the Terminal Heterocyclic Moiety of TVB Based Ligands Governs the Detection of Aβ Deposits Associated with the Arctic *APP* Mutation

**Figure 1:**
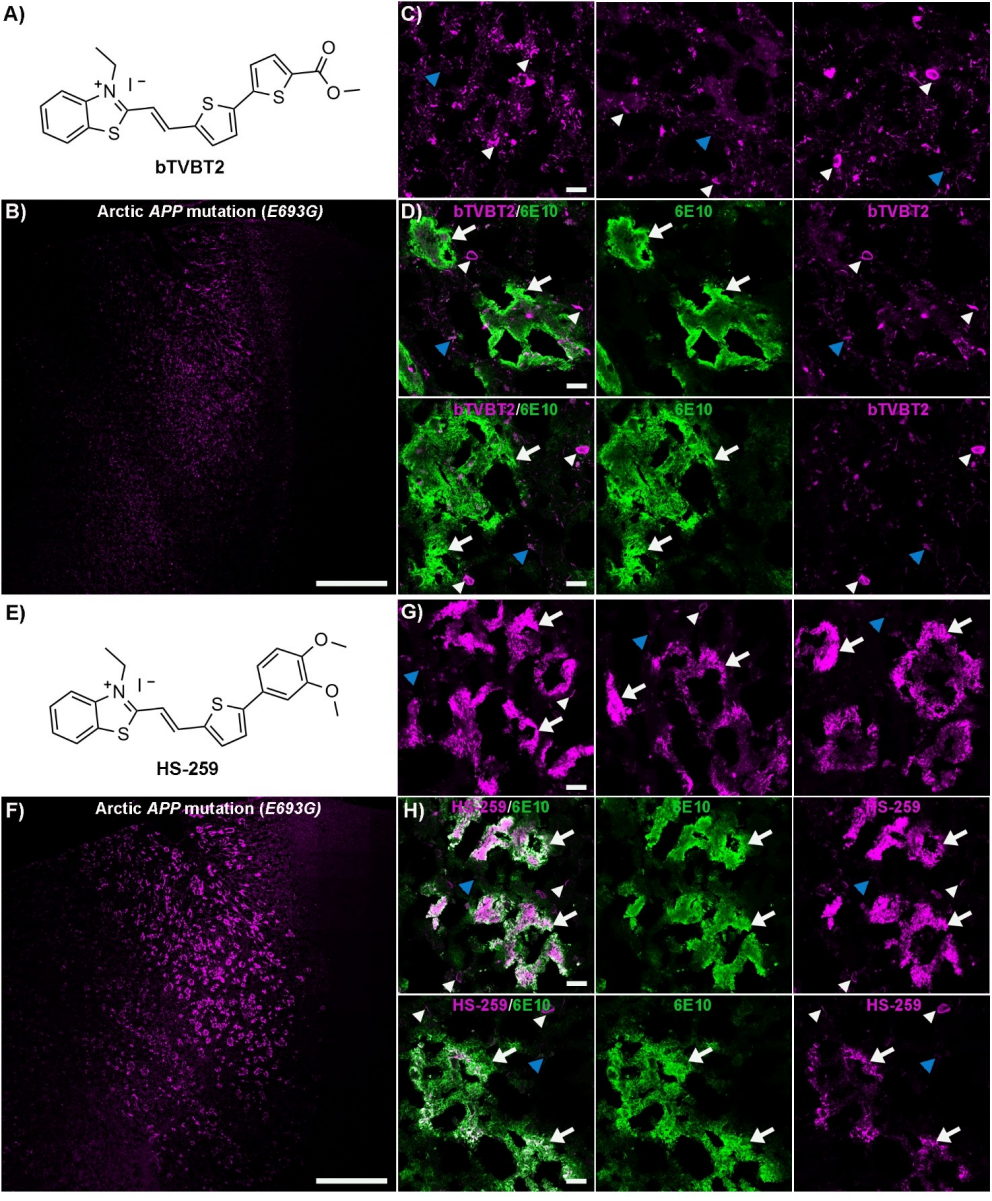
Staining of human brain tissue sections (frontal cortex) from an individual with diAD caused by the Arctic *APP E693G* mutation with bTVBT2 or HS‐259. **A**) Chemical structure of bTVBT2. **B**) Overview tile image of a brain tissue section from an individual with diAD caused by the Arctic *APP E693G* mutation stained with bTVBT2. **C**) Images of protein deposits stained with bTVBT2. **D**) Images of a tissue section co‐stained with bTVBT2 (magenta) and the Aβ antibody 6E10 (green). **E**) Chemical structure of HS‐259. **F**) Overview tile image of a brain tissue section from an individual with diAD caused by the Arctic *APP E693G* mutation stained with HS‐259. **G**) Images of different protein deposits stained with HS‐259. **H**) Images of a tissue section co‐stained with HS‐259 (magenta) and the Aβ antibody 6E10 (green). In C, D, G and H, Aβ deposits are indicated by white arrows, whereas tau aggregates are indicated by white arrowheads and autofluorescence from granular lipofuscin is indicated by blue arrowheads. Scale bars represent 1 mm (B and F) and 20 μm (C, D, G and H).

**Figure 2:**
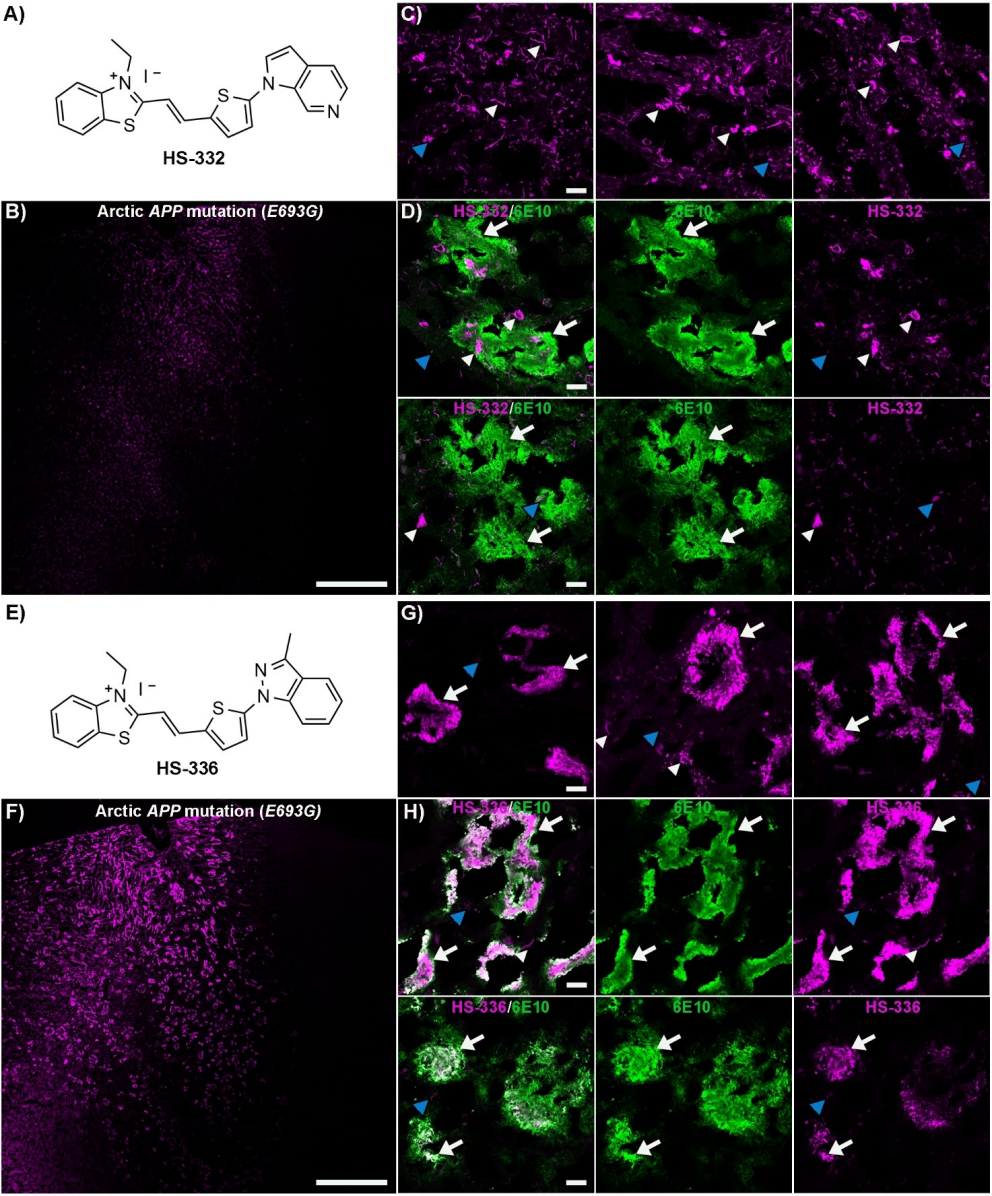
Staining of human brain tissue sections (frontal cortex) from an individual with diAD caused by the Arctic *APP E693G* mutation with HS‐332 or HS‐336. **A**) Chemical structure of HS‐332. **B**) Overview tile image of a brain tissue section from an individual with diAD caused by the Arctic *APP E693G* mutation stained with HS‐332. **C**) Images of protein deposits stained with HS‐332. **D**) Images of a tissue section co‐stained with HS‐332 (magenta) and the Aβ antibody 6E10 (green). **E**) Chemical structure of HS‐336. **F**) Overview tile image of a brain tissue section from an individual with diAD caused by the Arctic *APP E693G* mutation stained with HS‐336. **G**) Images of protein deposits stained by HS‐336. **H**) Images of a tissue section co‐stained with HS‐336 (magenta) and the Aβ antibody 6E10 (green). In C, D G and H, Aβ deposits are indicated by white arrows, whereas tau aggregates are indicated by white arrowheads and autofluorescence from granular lipofuscin is indicated by blue arrowheads. Scale bars represent 1 mm (B and F) and 20 μm (C, D, G and H).

To identify ligands that detect Aβ deposits that go undetected by conventional ligands, we tested a selection of previously reported[^49^, ^51^] TVB based ligands (Supporting Information (SI), Figure S1) on brain tissue sections from an individual with the Arctic *APP* mutation (*E693G*). All these ligands had the same TVB building block, but different terminal heterocyclic moieties. When analyzing the tissue sections, different staining pattern could be observed for the respective ligand and similar patterns as displayed by two different Aβ antibodies, 6E10,^[53]^ which has an epitope mapped to residues 5–7 of the Aβ peptide, and mAb158,^[54]^ an Aβ conformation dependent monoclonal antibody, were only obtained for some ligands (SI, Figure S2). bTVBT2, which has a terminal methyl thiophene‐2‐carboxylate moiety, displayed selective staining of structures resembling tau aggregates, whereas staining of immunopositive Aβ deposits was lacking (Figure 1A–D and SI, Figure S3). In contrast, for HS‐259, the ligand with a terminal 1,2‐dimethoxybenzene moiety, intense fluorescence from the ligand was observed from immunopositive Aβ deposits, as well as tau aggregates (Figure 1E–H and SI, Figure S3). Ligands HS‐208 and HS‐258 showed only partial overlap with the anti‐Aβ antibody 6E10 and from the Aβ deposits that were labelled, the fluorescence intensity from the ligand was rather weak compared to the intensity from the tau aggregates (SI, Figure S4). Furthermore, ligands HS‐205, HS‐212, HS‐253 and HS‐332 showed a similar selectivity towards tau aggregates as bTVBT2 (Figure 2A–D and SI, Figures S5–S6). HS‐336, which has a terminal 3‐methyl‐1*H*‐indazole moiety, displayed a similar staining pattern as observed for HS‐259, since HS‐336 fluorescence was observed from the majority of 6E10 positive Aβ deposits, as well as from tau aggregates (Figure 2E–H). Thus, the chemical nature of the terminal heterocyclic moiety had a great impact on the ligands’ ability to detect Aβ aggregates in the Arctic *APP* diAD case. Interestingly, in earlier studies using brain tissue sections from individuals with sAD,[^49^, ^51^] HS‐259, HS‐208, HS‐258 and HS‐336 showed binding to tau aggregates and some Aβ deposits, such as cored plaques and CAA lesions, whereas bTVBT2, HS‐205, HS‐207, HS‐212, HS‐253 and HS‐332 were selective for tau aggregates. Hence, the ligands that showed staining of certain Aβ pathologies in brain tissue sections from individuals with sAD also displayed staining of Aβ deposits in the Arctic *APP* diAD case. Furthermore, like in sAD, these ligands displayed different emission profiles when bound to Aβ or tau aggregates (SI, Figure S7).

For the two ligands, HS‐259 and HS‐336, showing the most prevalent staining of immunopositive Aβ deposits, the fluorescence from the centre of some deposits was less intense and bright fluorescence was mainly observed from the outer rim of these deposits (Figures 1G–H and 2G–H). Previous studies[^36^, ^38^] have shown that Aβ deposits in cases with the Arctic *APP* mutation display a variable content of differently modified Aβ peptides and this biochemical variation might influence the binding modes of HS‐259 and HS‐336 to the Aβ aggregates. Thus, specific binding modes of distinct ligands might be afforded due to a particular content of Aβ peptides within the deposit and as recently shown,^[52]^ the detection of distinct Aβ deposits by a specific thiophene‐based ligand can be associated to a distinct composition of specific Aβ peptides within the aggregates.

### The Chemical Composition of the Terminal Nitrogen Containing Fused 5‐ and 6‐Membered Ring Governs the Selectivity of TVB Based Ligands Towards Aβ Deposits Associated with the Arctic *APP* Mutation

**Scheme 1:**
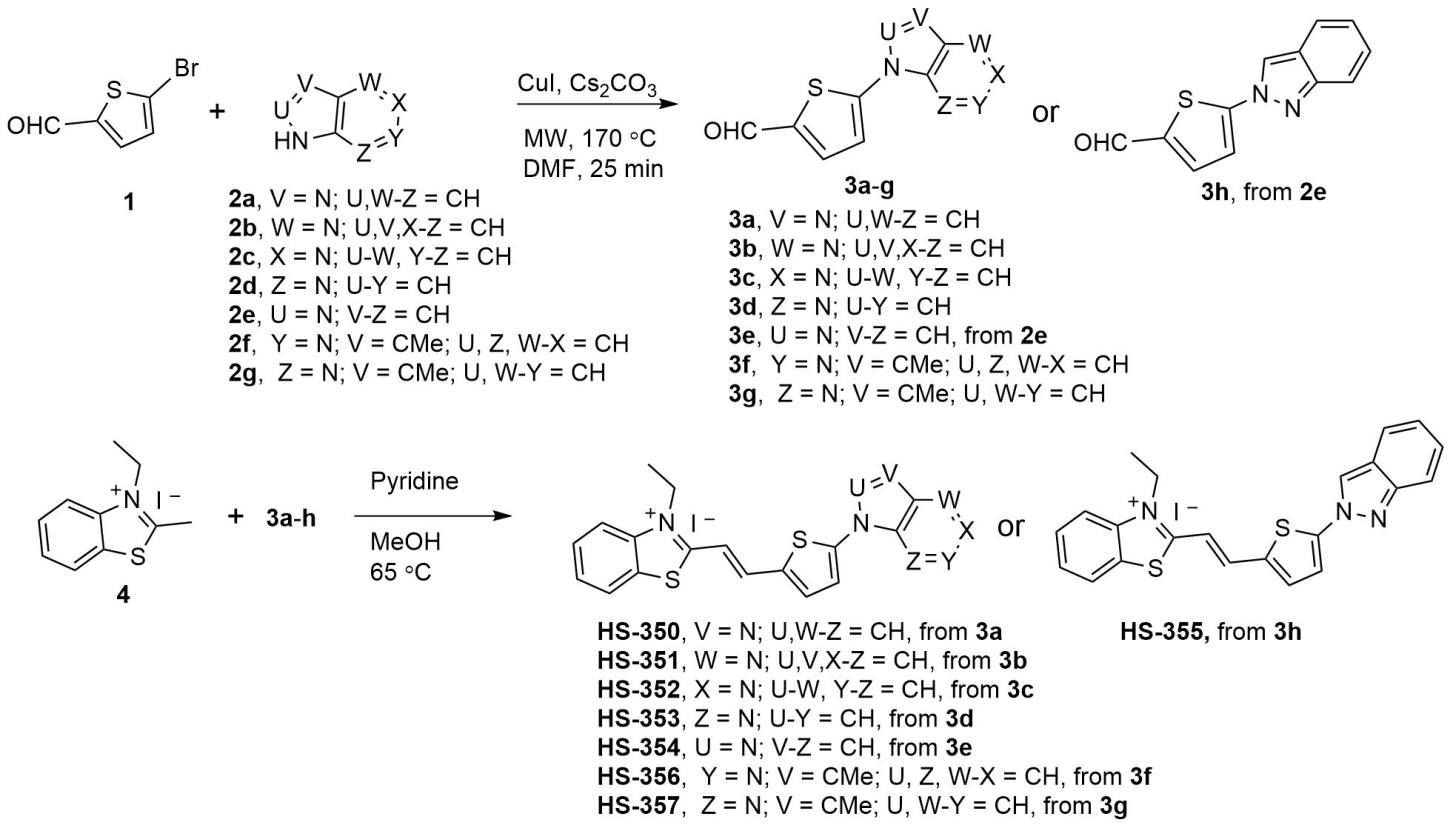
Synthesis of ligands HS‐350 to HS‐357. General procedure A for the synthesis of **3 a**–**h** with 1 and **2 a**–**g** from an N‐arylation reaction. General procedure B for the condensation synthesis of HS‐350 to HS‐357 from **4** and **3 a**–**h**.

To further pinpoint the molecular requirements for achieving a fluorescent ligand that detects Aβ deposits in brain tissue sections from an individual with the Arctic *APP* mutation, we next synthesized some additional TVB based ligands. Since the two structurally related ligands HS‐332 and HS‐336 displayed strikingly opposite results in the histological staining, it seemed like the positioning of the nitrogen in the terminal fused 5‐ and 6‐membered ring and/or the addition of a methyl substituent on this moiety are important chemical determinants for achieving a ligand that could detect these types of Aβ deposits. Therefore, additional ligands having different terminal nitrogen containing fused 5‐ and 6‐membered rings with different positioning of the nitrogen atoms, as well as an additional methyl substituent on these moieties, were synthesized. First, the building blocks **3 a**–**h** were created by *N*‐arylation with slight modifications of the Ullman reaction conditions (Scheme 1). Second, the TVB based ligands were assembled by a condensation reaction of 3‐ethyl‐2‐methylbenzothiazolium iodide **4** and the respective building blocks **3 a‐h** using pyridine as base, rendering the ligands HS‐350, HS‐351, HS‐352, HS‐353, HS‐354, HS‐355, HS‐356, and HS‐357 (Scheme 1). All ligands were isolated as the E‐isomer and when dissolved in DMSO (1.5 mM ligand), and further diluted in phosphate buffered saline (PBS) pH 7.4 to a final concentration of 30 μM, the ligands displayed absorption maximum ranging from 422 nm–462 nm, as well as emission maximum from 560 nm–600 nm (SI, Figure S8 and Table S1).

**Figure 3:**
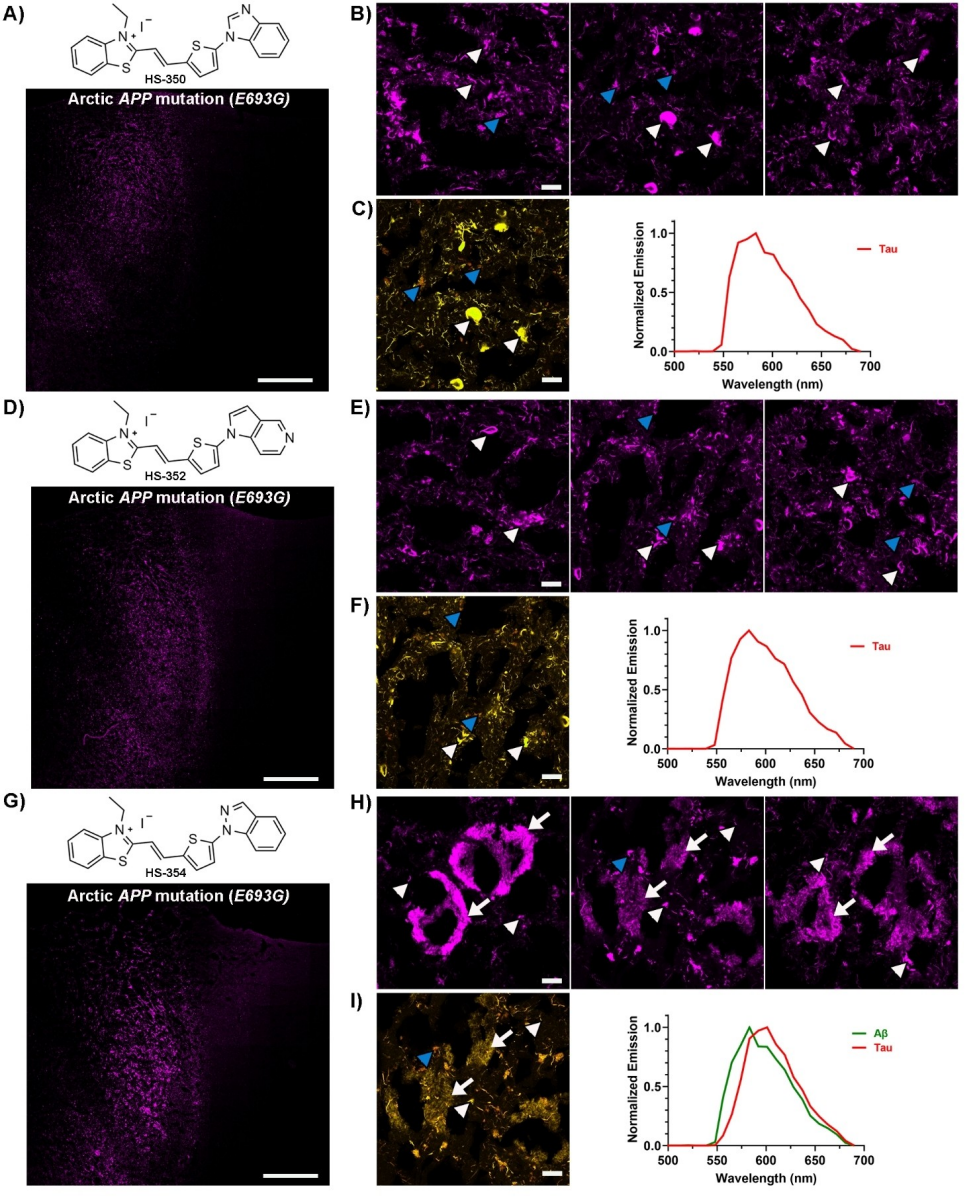
Staining of human brain tissue sections (frontal cortex) from an individual with diAD caused by the Arctic *APP E693G* mutation with HS‐350, HS‐352 or HS‐354. **A**) Chemical structure of HS‐350 and overview tile image of a brain tissue section from an individual with diAD caused by the Arctic *APP E693G* mutation stained with HS‐350. **B**) Images of protein deposits stained with HS‐350. **C**) Spectral image and the emission spectrum of HS‐350 when bound to tau aggregates (red). **D**) Chemical structure of HS‐352 and overview tile image of a brain tissue section from an individual with diAD caused by the Arctic *APP E693G* mutation stained with HS‐352. **E**) Images of protein deposits stained with HS‐352. **F**) Spectral image and the emission spectrum of HS‐352 when bound to tau aggregates (red). **G**) Chemical structure of HS‐354 and overview tile image of a brain tissue section from an individual with diAD caused by the Arctic *APP E693G* mutation stained with HS‐354. **H**) Images of protein deposits stained with HS‐354. **I**) Spectral image and the emission spectrum of HS‐354 when bound to Aβ deposits (green) or tau aggregates (red). In B, C, E, F, H and I, Aβ deposits are indicated by white arrows, whereas tau aggregates are indicated by white arrowheads and autofluorescence from granular lipofuscin is indicated by blue arrowheads. Scale bars represent 1 mm (A, D and G) and 20 βm (B, C, E, F, H and I).

**Figure 4:**
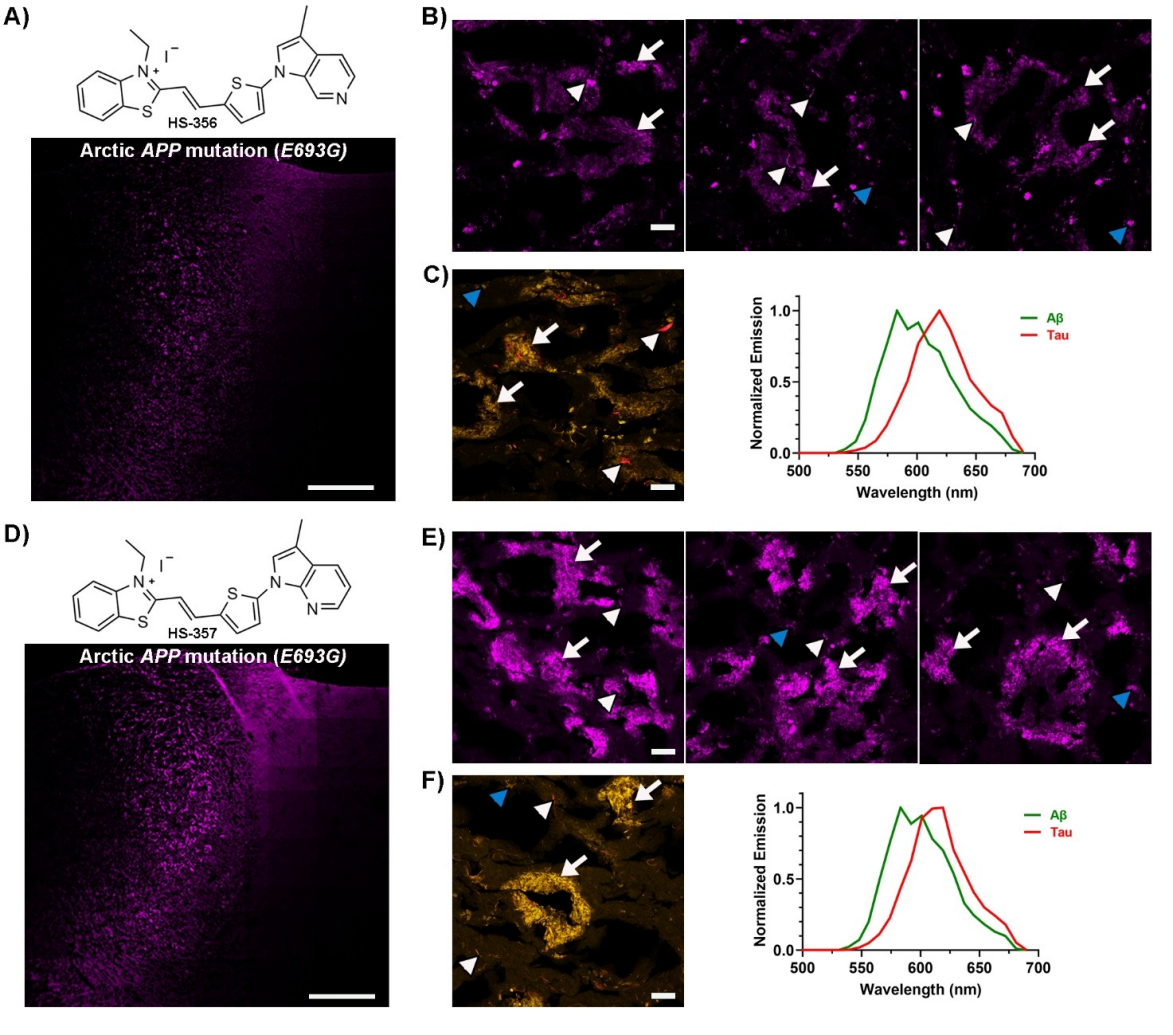
Staining of human brain tissue sections (frontal cortex) from an individual with diAD caused by the Arctic *APP E693G* mutation with HS‐356 or HS‐357. **A**) Chemical structure of HS‐356 and overview tile image of a brain tissue section from an individual with diAD caused by the Arctic *APP E693G* mutation stained with HS‐356. **B**) Images of protein deposits stained with HS‐356. **C**) Spectral image and the emission spectrum of HS‐356 when bound to Aβ deposits (green) or tau aggregates (red). **D**) Chemical structure of HS‐357 and overview tile image of a brain tissue section from an individual with diAD caused by the Arctic *APP E693G* mutation stained with HS‐357. **E**) Images of protein deposits stained with HS‐357. **F**) Spectral image and the emission spectrum of HS‐357 when bound to Aβ deposits (green) or tau aggregates (red). In B, C, E and F, Aβ deposits are indicated by white arrows, whereas tau aggregates are indicated by white arrowheads and autofluorescence from granular lipofuscin is indicated by blue arrowheads. Scale bars represent 1 mm (A and D) and 20 μm (B, C, E and F).

When tested on brain tissue sections from an individual with the Arctic *APP* mutation, all unmethylated HS‐332 and HS‐336 analogues displayed distinct staining patterns (SI, Figure S9). HS‐350, HS‐351 and HS‐352 showed a similar selectivity towards tau aggregates as HS‐332, and fluorescence from Aβ aggregates was lacking (Figure 3 and SI, Figure S10). In contrast, with the other three ligands, HS‐353, HS‐354 and HS‐355, both Aβ deposits and tau aggregates could be identified due to distinct fluorescence signatures from the ligands (Figure 3 and SI, Figure S10). Hence, the positioning of nitrogen in the terminal fused 5‐ and 6‐membered ring had a great impact on the ligands' ability to detect Aβ aggregates. A similar effect has also been observed for the tau PET tracer PI‐2620, since there was a strong correlation between the nitrogen atom position in the tricyclic core of the ligand and the ligand's ability to recognize tau aggregates.[^55^, ^56^] The most frequent staining of Aβ deposits was observed for HS‐354, the ligand having the same positioning of the two nitrogen atoms in the terminal fused 5‐ and 6‐membered ring as HS‐336, but lacking the methyl substitution in the 3‐position of the indazole ring. However, the staining was not as abundant as for HS‐336, suggesting that the methyl substituent in this position was also important for achieving superior detection of Aβ deposits associated with the Arctic *APP* mutation. This indication was verified when testing the two additional ligands with a methyl substituent, HS‐356 and HS‐357 (Figure 4). In contrast to HS‐332, the methylated analogue HS‐356 displayed fluorescence from some Aβ deposits and a distinct spectral signature from these deposits, as well as from tau aggregates, was observed (Figure 4A–C). Likewise, the methylated version of HS‐353, denoted HS‐357, showed a more abundant staining of Aβ deposits compared to its unmethylated analogue (Figure 4D–F). Hence, both the distinct positioning of the two nitrogen atoms in the terminal fused 5‐ and 6‐membered ring, as well as the specific methyl substitution, seem to be essential chemical determinants for achieving TVB ligands that are optimized for detection of Aβ deposits in the Arctic *APP* diAD case.

### Methoxy Substituents on the Terminal 6‐Membered Ring Improve the Detection of Aβ Deposits Associated with the Arctic *APP* Mutation

**Figure 5:**
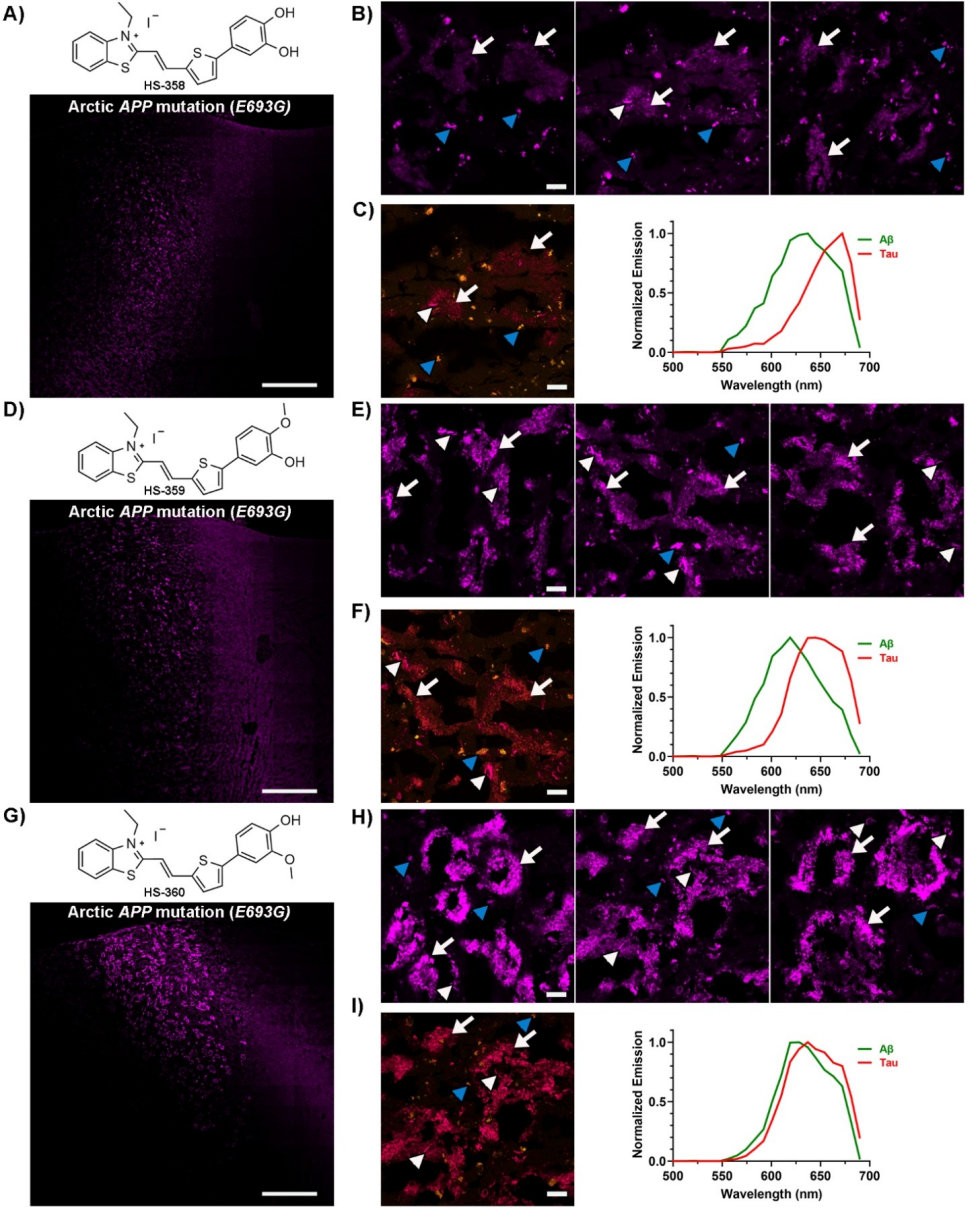
Staining of human brain tissue sections (frontal cortex) from an individual with diAD caused by the Arctic *APP E693G* mutation with HS‐358, HS‐359 or HS‐360. **A**) Chemical structure of HS‐358 and overview tile image of a brain tissue section from an individual with diAD caused by the Arctic *APP E693G* mutation stained with HS‐358. **B**) Images of protein deposits stained with HS‐358. **C**) Spectral image and the emission spectrum of HS‐358 when bound to Aβ deposits (green) or tau aggregates (red). **D**) Chemical structure of HS‐359 and overview tile image of a brain tissue section from an individual with diAD caused by the Arctic *APP E693G* mutation stained with HS‐359. **E**) Images of protein deposits stained with HS‐359. **F**) Spectral image and the emission spectrum of HS‐359 when bound to Aβ deposits (green) or tau aggregates (red). **G**) Chemical structure of HS‐360 and overview tile image of a brain tissue section from an individual with diAD caused by the Arctic *APP E693G* mutation stained with HS‐360. **H**) Images of protein deposits stained with HS‐360. **I**) Spectral image and the emission spectrum of HS‐360 when bound to Aβ deposits (green) or tau aggregates (red). In B, C, E, F, H and I, Aβ deposits are indicated by white arrows, whereas tau aggregates are indicated by white arrowheads and autofluorescence from granular lipofuscin is indicated by blue arrowheads. Scale bars represent 1 mm (A, D and G) and 20 μm (B, C, E, F, H and I).

**Scheme 2:**
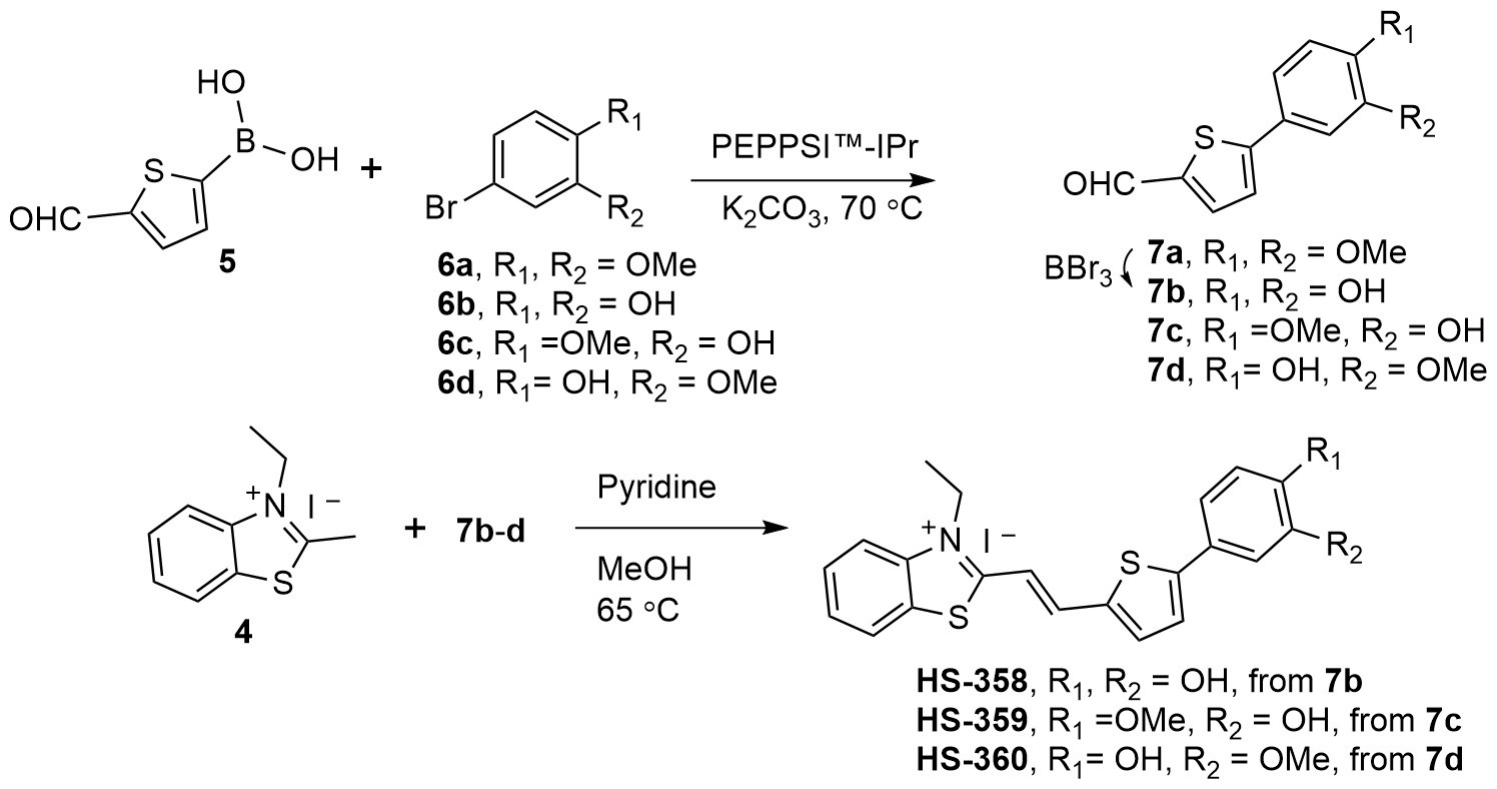
Synthesis of ligands HS‐358 to HS‐360. General procedure for the synthesis of **7 a** or **7 c**–**d** with **5** and **6 a** or **6 c**–**d** from a coupling reaction, **7 b** was achieved from **7 a** after the demethylation with BBr_3_. General procedure for the condensation synthesis of HS‐358 to HS‐360 from **4** and **7 b**–**d**.

As the methyl substitution on the terminal nitrogen containing fused 5‐ and 6‐membered ring was an important chemical moiety for detection of Aβ deposits in the Arctic *APP* diAD case, we next explored the potential role of the methoxy substituents of the terminal benzene moiety of HS‐259 for detection of these deposits. In this regard, three analogues to HS‐259, denoted HS‐358, HS‐359 and HS‐360 (Figure 5), were synthesized in a similar fashion as for the TVB based ligands described above (Scheme 2). For HS‐358, the two methoxy substituents were replaced with hydroxyl groups, whereas for HS‐359 and HS‐360 the methoxy substituent in the meta (HS‐359) or para (HS‐360) position of the benzyl ring was changed to a hydroxyl group. When dissolved in DMSO (1.5 mM ligand) and further diluted in PBS pH 7.4 to a final concentration of 30 μM, the absorption maximum of the ligands was ranging from 452 nm–465 nm, and the emission maximum from 631 nm–660 nm (SI, Figure S8 and Table S1).

When applied for histological staining of the Arctic APP diAD case, all ligands displayed staining towards Aβ deposits, as well as tau aggregates, and similar to HS‐259, all ligands displayed distinct spectral signatures from the respective aggregates (Figure 5 and SI, Figure S11). For HS‐358, the ligand with the terminal catechol moiety, the fluorescence from the Aβ deposits was rather weak compared to HS‐259 (Figure 5A–C), indicating that removal of the methyl groups influenced the detection of these deposits. A slightly more abundant staining of the Aβ aggregates was observed for HS‐359, whereas a similar distinct staining of the Aβ deposits as observed for HS‐259 was achieved with HS‐360. Thus, the methoxy group in the meta position of the terminal benzene moiety appeared to be the most crucial chemical determinant for achieving a TVB based ligand for optical detection of Aβ deposits associated with the Arctic *APP* mutation.

### TVB Based Ligands in Combination with a Conventional Ligand Allows a More Precise Assignment of Aβ Deposits

**Figure 6:**
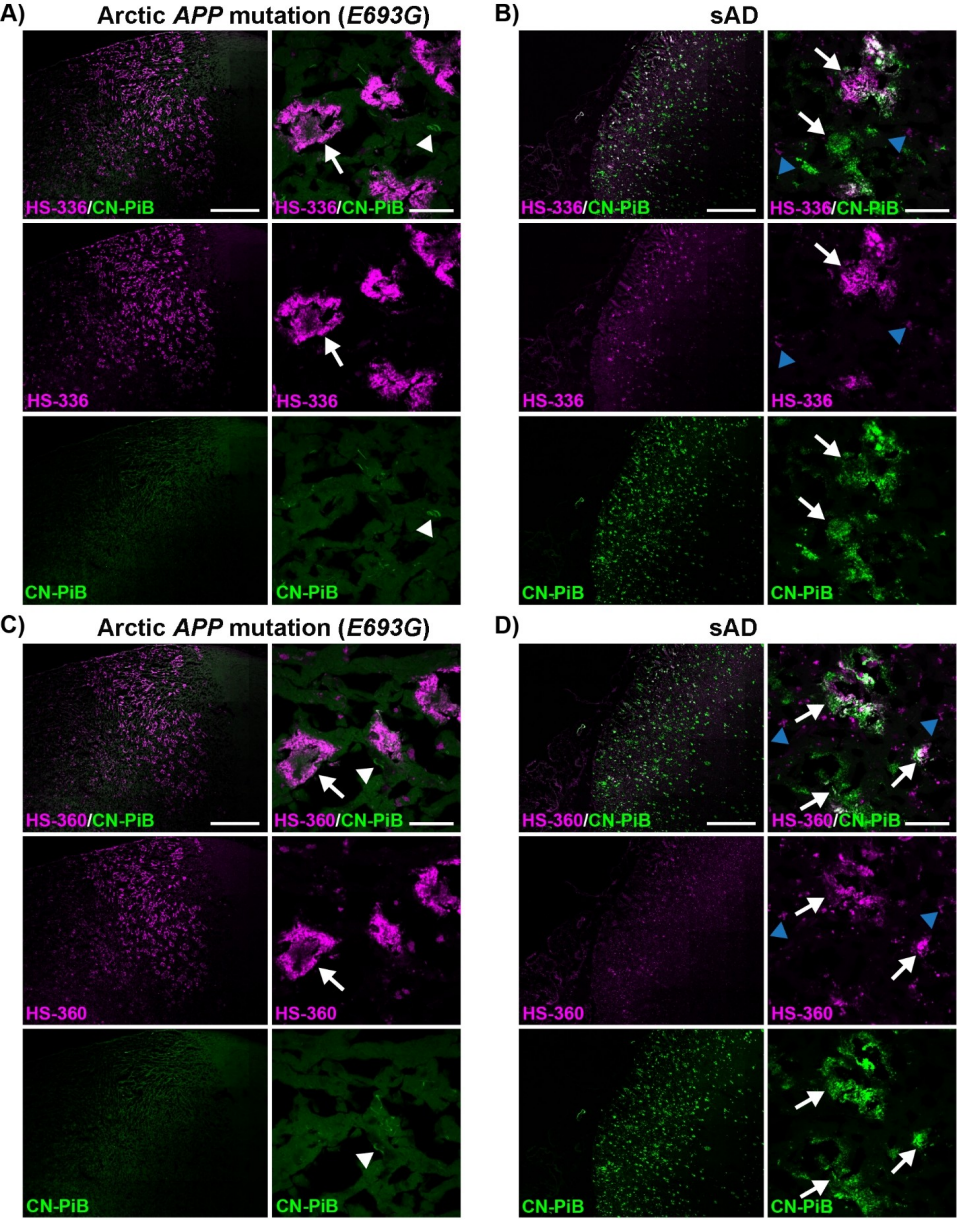
Staining of human brain tissue sections (frontal cortex) from individuals with sAD or diAD caused by the Arctic *APP E693G* mutation with HS‐336 or HS‐360 in combination with CN‐PiB. **A**–**B**) Overview tile image (left) and zoom‐in image (right) of protein deposits stained with HS‐336 (magenta) and CN‐PiB (green) in a brain tissue section from individuals diagnosed with diAD caused by the Arctic *APP E693G* mutation (A) or sAD (B). **C**–**D**) Overview tile image (left) and zoom‐in image (right) of protein deposits stained with HS‐360 (magenta) and CN‐PiB (green) in individuals diagnosed with diAD caused by the Arctic *APP E693G* mutation (C) or sAD (d). Aβ deposits are indicated by white arrows, whereas tau aggregates are indicated by white arrowheads and autofluorescence from lipofuscin granules is indicated by blue arrowheads. Scale bars represent 1 mm (left panels in A–D) and 20 μm (right panels in A–D).

To test our hypothesis that several ligands are necessary for an accurate assignment of Aβ deposits in AD, two of the ligands, HS‐336 and HS‐360, were next tested in combination with the conventional ligand CN‐PiB^[57]^ on brain tissue sections (frontal cortex) from individuals with sAD or diAD caused by the Arctic *APP* mutation. When comparing the brain tissue sections that were stained by the respective dual‐ligand staining protocol, distinct staining patterns were observed for the respective cases (Figure 6). CN‐PiB fluorescence was lacking from the Aβ deposits in the Arctic *APP* diAD case (Figure 6A and C), whereas intense CN‐PiB fluorescence was observed from Aβ deposits in the sAD tissue sections (Figure 6B and D). For HS‐336 and HS‐360, an opposite staining pattern was observed as Aβ deposits associated with the Arctic *APP* mutation were clearly visualized by the fluorescence from the respective ligand whereas only some of the CN‐PiB positive Aβ deposits in the sAD tissue sections were labelled (Figure 6). For most Aβ deposits in the sAD case, the TVB ligands only showed partial overlap with CN‐PiB, and in comparison with HS‐360, HS‐336 showed more pronounced staining. In fact, this ligand also labelled a small number of Aβ deposits in the sAD case that were negative with CN‐PiB. Overall, these dual‐ligand staining experiments verified that a combination of ligands is necessary to assign different Aβ aggregates in AD. From a clinical perspective, these findings might have several implications, since PiB is one of the most abundant PET tracers used for the diagnosis of AD^[15]^ and the preferred tracer for evaluating the effect of anti‐amyloid antibody immunotherapy.^[58]^ Thus, having additional PET tracers that detect PiB negative Aβ aggregates might result in a more accurate diagnostics of AD, as well as aid in assessing the potential of pharmaceutical inventions targeting aggregated Aβ pathologies. For the latter, further development of efficient PET tracers based on the molecular scaffold presented herein might assist in evaluating the therapeutic effect of lecanemab (BAN2401),^[58]^ a monoclonal antibody stimulating the clearance of Aβ deposits from the brain, since some of the ligands clearly recognize Aβ aggregates detected by mAb158,^[54]^ the murine version of lecanemab (BAN2401).

## Conclusions

In conclusion, we have shown that superior ligand‐based optical detection of a specific type of Aβ deposits found in the brain of patients with the Arctic *APP* mutation is highly dependent on the chemical nature of the ligand. TVB based ligands with distinct terminal heterocyclic moieties displayed staining of these Aβ deposits, and further chemical exploration of the ligands revealed that the positioning of nitrogen atoms and methyl substituents were vital chemical determinants for achieving optimal detection. In addition, dual‐staining protocols with a TVB based ligand and the conventional ligand CN‐PiB verified that the use of multiple ligands is essential for a more precise assignment of Aβ deposits in AD. Our findings show how minor chemical modifications of TVB based ligands influence their performance when it comes to detect distinct Aβ deposits, and the ligands presented herein also enable an expansion of the toolbox of fluorescent ligands that can be utilized for fluorescent assignment of protein aggregates. We foresee that the TVB ligands will aid in assigning distinct Aβ deposits, and that our findings can be relevant for the development of novel PET tracers for accurate clinical diagnostics of AD.

## Experimental Section

### Synthesis of TVB Based Ligands

The synthesis of bTVBT2, HS‐205, HS‐208, HS‐212, HS‐253, HS‐258, HS‐259, HS‐332 and HS‐336 has been presented elsewhere.[^49^, ^51^] The detailed procedure for the synthesis of HS‐350, HS‐351, HS‐352, HS‐353, HS‐354, HS‐355, HS‐356, HS‐357, HS‐358, HS‐359 and HS‐360 can be found in the supporting information.

### Optical Characterization of the Ligands

Stock solutions of ligands (1.5 mM in DMSO) were diluted to 30 μM in phosphate buffered saline (PBS, 10 mM phosphate, 140 mM NaCl, 2.7 mM KCl, pH 7.4). Absorption‐ and emission spectra of the ligands were collected using an Infinite M1000 Pro microplate reader (Tecan,).

### Ligand Staining of Brain Tissue Sections

The experiments involving human brain tissue were reviewed and approved by the Indiana University Institutional Review Board, as well as the Regional Ethical Committee in Uppsala, Sweden, and informed consent was obtained from the patient or their next of kin. The experiments carried out at Linköping University were reviewed and approved by a national ethical committee (approval number 2020–01197). Sections of frontal cortex (10 μm) from an individual with diAD associated with the Arctic *APP* mutation (SI, Table S2) were fixed in 96 % EtOH for 10 min at RT, incubated 10 min in 50 % EtOH followed by 10 min in dH_2_O at RT and then 10 min in PBS. The sections were incubated with 200 nM of the respective ligand in PBS for 30 min at RT. The sections were then washed with PBS three times and mounted with Dako mounting medium for fluorescence (Agilent). The mounting medium was allowed to solidify at least overnight before sealing the cover slips with nail polish. The sections were analyzed using an inverted Zeiss LSM 780 laser scanning confocal microscope (Zeiss) using the following the following settings:

HS‐205 and HS‐212 (Excitation: 520 nm).

bTVBT2, HS‐208, HS‐253, HS‐258, HS‐259, HS‐332, HS‐336, HS‐350, HS‐351, HS‐352, HS‐353, Hs‐354; HS‐355, HS‐356, HS‐357, HS‐358, HS‐359 and HS‐360 (Excitation: 535 nm).

For all ligands the emission was collected from 551 nm–705 nm.

### Ligand and Antibody Double Staining

Frozen sections (10 μm) of frontal cortex brain tissue from a patient diagnosed with diAD caused by the Arctic *APP* mutation (SI, Table S2) were prepared. The frozen brain tissue sections were fixed in 70 % EtOH for 3 min at 4 °C and then incubated for 2×2 min in dH_2_O and 10 min in PBS at RT. Non‐specific binding was blocked by incubating the sections in PBS with 0.1 % triton X‐100 and 5 % normal goat serum (blocking buffer) for 1 h at RT. Antibody 6E10 (Biolegend) or mAb158 (Dag Sehlin, Uppsala University) was diluted 1 : 1000 in blocking buffer and added to the sections. After incubation over night at 4 °C, the sections were washed in PBS with 0.1 % triton X‐100 (PBS−T) for 3×10 min and then incubated for 1 h at RT with goat anti‐mouse or goat‐anti‐rabbit secondary antibody conjugated with Alexa Fluor 488 or Alexa Fluor 647 (ThermoFisher). The secondary antibody was diluted 1 : 500 in blocking buffer. After washing the sections in PBS for 3×10 min, they were incubated with 200 nM of the respective ligand for 30 min at RT. The sections were then washed with PBS three times and mounted with Dako mounting medium for fluorescence (Agilent). The mounting medium was allowed to solidify at least overnight before analyzing the result using an inverted Zeiss LSM 780 laser scanning confocal microscope (Zeiss) using the following settings:

6E10 (Alexa Fluor 488): Excitation: 490 nm; Emission: 495 nm–550 nm.

Ligands bTVBT2, HS‐205, HS‐208, HS‐212, HS‐253, HS‐258, HS‐259 and HS‐332: Excitation: 565 nm; Emission: 595 nm–708 nm.

6E10 (Alexa Fluor 647): Excitation: 633 nm; Emission: 638 nm–755 nm.

Ligand HS‐336: Excitation: 520 nm; Emission: 525 nm–637 nm.

### Ligand Double Staining

Sections of frontal cortex (10 μm) from a sAD patient (SI, Table S2) or a patient diagnosed with diAD associated with the Arctic *APP* mutation (SI, Table S2) were fixed in 99.7 % EtOH, rehydrated and incubated in PBS as described above. The sections were then incubated with different combination of ligands, 200 nM HS‐336 and 200 nM CN‐PiB or 200 nM HS‐360 and 200 nM CN‐PiB, in PBS for 30 min at RT. The sections were then washed with PBS three times and mounted with Dako mounting medium for fluorescence (Agilent). The mounting medium was allowed to solidify at least overnight before sealing the cover slips with nail polish. The sections were analyzed using an inverted Zeiss LSM 780 laser scanning confocal microscope (Zeiss) using the following settings:

CN‐PiB: Excitation: 405 nm Emission: 410 nm–514 nm.

HS‐336: Excitation: 535 nm Emission: 541 nm–705 nm.

HS‐360: Excitation: 535 nm Emission: 541 nm–705 nm.

## Conflict of Interests

The authors declare no conflict of interest.

1

## Supporting information

As a service to our authors and readers, this journal provides supporting information supplied by the authors. Such materials are peer reviewed and may be re‐organized for online delivery, but are not copy‐edited or typeset. Technical support issues arising from supporting information (other than missing files) should be addressed to the authors.

Supporting Information

## Data Availability

The data that support the findings of this study are available from the corresponding author upon reasonable request.

## References

[1] V. M. Lee , M. Goedert , J. Q. Trojanowski , Annu. Rev. Neurosci. 2001, 24, 1121–1159. 11520930 10.1146/annurev.neuro.24.1.1121

[2] M. Goedert , Nat. Rev. Neurosci. 2001, 2, 492–501. 11433374 10.1038/35081564

[3] M. Jucker , L. C. Walker , Nature 2013, 501, 45–51. 24005412 10.1038/nature12481 PMC3963807

[4] D. M. Wilson 3rd , M. R. Cookson , L. Van Den Bosch , H. Zetterberg , D. M. Holtzman , I. Dewachter , Cell 2023, 186, 693–714. 36803602 10.1016/j.cell.2022.12.032

[5] P. S. Vassar , C. F. Culling , Arch. Pathol. 1959, 68, 487–498. 13841452

[6] S. D. Styren , R. L. Hamilton , G. C. Styren , W. E. Klunk , J. Histochem. Cytochem. 2000, 48, 1223–1232. 10950879 10.1177/002215540004800906

[7] W. E. Klunk , B. J. Bacskai , C. A. Mathis , S. T. Kajdasz , M. E. McLellan , M. P. Frosch , M. L. Debnath , D. P. Holt , Y. Wang , B. T. Hyman , J. Neuropathol. Exp. Neurol. 2002, 61, 797–805. 12230326 10.1093/jnen/61.9.797

[8] A. S. Crystal , B. I. Giasson , A. Crowe , M.-P. Kung , Z.-P. Zhuang , J. Q. Trojanowski , V. M. Lee , J. Neurochem. 2003, 86, 1359–1368. 12950445 10.1046/j.1471-4159.2003.01949.x

[9] M. Hintersteiner , A. Enz , P. Frey , A.-L. Jaton , W. Kinzy , R. Kneuer , U. Neumann , M. Rudin , M. Staufenbiel , M. Stoeckli , K.-H. Wiederhold , H.-U. Gremlich , Nat. Biotechnol. 2005, 23, 577–583. 15834405 10.1038/nbt1085

[10] E. E. Nesterov , J. Skoch , B. T. Hyman , W. E. Klunk , B. J. Bacskai , T. M. Swager , Angew. Chem. Int. Ed. 2005, 44, 5452–5456. 10.1002/anie.200500845 16059955

[11] W. M. Chang , M. Dakanali , C. C. Capule , C. J. Sigurdson , J. Yang , E. A. Theodorakis , ACS Chem. Neurosci. 2011, 2, 249–255. 21743829 10.1021/cn200018v PMC3129977

[12] K. Cao , M. Farahi , M. Dakanali , W. M. Chang , C. J. Sigurdson , E. A. Theodorakis , J. Yang , J. Am. Chem. Soc. 2012, 134, 17338–17341. 22866977 10.1021/ja3063698 PMC3480552

[13] M. Cui , M. Ono , H. Watanabe , H. Kimura , B. Liu , H. Saji , J. Am. Chem. Soc. 2014, 136, 3388–3394. 24555862 10.1021/ja4052922

[14] H. Fu , M. Cui , P. Tu , Z. Pan , B. Liu , Chem. Commun. 2014, 50, 11875–11878. 10.1039/c4cc04907a 25156492

[15] W. E. Klunk , H. Engler , A. Nordberg , Y. Wang , G. Blomqvist , D. P. Holt , M. Bergström , I. Savitcheva , G. F. Huang , S. Estrada , B. Ausén , M. L. Debnath , J. Barletta , J. C. Price , J. Sandell , B. J. Lopresti , A. Wall , P. Koivisto , G. Antoni , C. A. Mathis , B. Långström , Ann. Neurol. 2004, 55, 306–319. 14991808 10.1002/ana.20009

[16] M. Koole , D. M. Lewis , C. Buckley , N. Nelissen , M. Vandenbulcke , D. J. Brooks , R. Vandenberghe , K. Van Laere , J. Nucl. Med. 2009, 50, 818–822. 19372469 10.2967/jnumed.108.060756

[17] N. Nelissen , K. Van Laere , L. Thurfjell , R. Owenius , M. Vandenbulcke , M. Koole , G. Bormans , D. J. Brooks , R. Vandenberghe , J. Nucl. Med. 2009, 50, 1251–1259. 19617318 10.2967/jnumed.109.063305

[18] Z. Cselényi , M. E. Jönhagen , A. Forsberg , C. Halldin , P. Julin , M. Schou , P. Johnström , K. Varnäs , S. Svensson , L. Farde , J. Nucl. Med. 2012, 53, 415–424. 22323782 10.2967/jnumed.111.094029

[19] W. Zhang , J. Arteaga , D. K. Cashion , G. Chen , U. Gangadharmath , L. F. Gomez , D. Kasi , C. Lam , Q. Liang , C. Liu , V. P. Mocharla , F. Mu , A. Sinha , A. K. Szardenings , E. Wang , J. C. Walsh , C. Xia , C. Yu , T. Zhao , H. C. Kolb , J. Alzheimer's Dis. 2012, 31, 601–612. 22683529 10.3233/JAD-2012-120712

[20] M. Maruyama , H. Shimada , T. Suhara , H. Shinotoh , B. Ji , J. Maeda , M. R. Zhang , J. Q. Trojanowski , V. M. Lee , M. Ono , K. Masamoto , H. Takano , N. Sahara , N. Iwata , N. Okamura , S. Furumoto , Y. Kudo , Q. Chang , T. C. Saido , A. Takashima , J. Lewis , M. K. Jang , I. Aoki , H. Ito , M. Higuchi , Neuron 2013, 79, 1094–1108. 24050400 10.1016/j.neuron.2013.07.037 PMC3809845

[21] A. M. Walji , E. D. Hostetler , H. Selnick , Z. Zeng , P. Miller , I. Bennacef , C. Salinas , B. Connolly , L. Gantert , M. Holahan , S. O'Malley , M. Purcell , K. Riffel , J. Li , J. Balsells , J. A. OBrien , S. Melquist , A. Soriano , X. Zhang , A. Ogawa , S. Xu , E. Joshi , J. Della Rocca , F. J. Hess , J. Schachter , D. Hesk , D. Schenk , A. Struyk , K. Babaoglu , T. G. Lohith , Y. Wang , K. Yang , J. Fu , J. L. Evelhoch , P. J. Coleman , J. Med. Chem. 2016, 59, 4778–4789. 27088900 10.1021/acs.jmedchem.6b00166

[22] C. Aguero , M. Dhaynaut , M. D. Normandin , A. C. Amaral , N. J. Guehl , R. Neelamegam , M. Marquie , K. A. Johnson , G. El Fakhri , M. P. Frosch , T. Gómez-Isla , Acta Neuropathol. 2019, 7, 37. 10.1186/s40478-019-0686-6 PMC6410510 30857558

[23] M. Habashi , S. Vutla , K. Tripathi , S. Senapati , P. S. Chauhan , A. Haviv-Chesner , M. Richman , S. A. Mohand , V. Dumulon-Perreault , R. Mulamreddy , E. Okun , J. H. Chill , B. Guérin , W. D. Lubell , S. Rahimipour Proc. Natl. Acad. Sci. USA 2022, 119, e2210766119. 36442093 10.1073/pnas.2210766119 PMC9894226

[24] A. T. Petkova , R. D. Leapman , Z. Guo , W.-M. Yau , M. P. Mattson , R. Tycko , Science 2005, 307, 262–265. 15653506 10.1126/science.1105850

[25] A. K. Paravastu , R. D. Leapman , W.-M. Yau , R. Tycko Proc. Natl. Acad. Sci. USA 2008, 105, 18349–18354. 19015532 10.1073/pnas.0806270105 PMC2587602

[26] C. Sachse , M. Fändrich , N. Grigorieff Proc. Natl. Acad. Sci. USA 2008, 105, 7462–7466. 18483195 10.1073/pnas.0712290105 PMC2396686

[27] J.-X. Lu , W. Qiang , W.-M. Yau , C. D. Schwieters , S. C. Meredith , R. Tycko , Cell 2013, 154, 1257–1268. 24034249 10.1016/j.cell.2013.08.035 PMC3814033

[28] M. Schmidt , A. Rohou , K. Lasker , J. K. Yadav , C. Schiene-Fischer , M. Fändrich , N. Grigorieff , Proc. Natl. Acad. Sci. USA 2015, 112, 11858–11863. 26351699 10.1073/pnas.1503455112 PMC4586870

[29] W. Qiang , W.-M. Yau , J.-X. Lu , J. Collinge , R. Tycko , Nature 2017, 541, 217–221. 28052060 10.1038/nature20814 PMC5233555

[30] L. Gremer , D. Schölzel , C. Schenk , E. Reinartz , J. Labahn , R. B. G. Ravelli , M. Tusche , C. Lopez-Iglesias , W. Hoyer , H. Heise , D. Willbold , G. F. Schröder , Science 2017, 358, 116–119. 28882996 10.1126/science.aao2825 PMC6080689

[31] Y. Yang , D. Arseni , W. Zhang , M. Huang , S. Lövestam , M. Schweighauser , A. Kotecha , A. G. Murzin , S. Y. Peak-Chew , J. Macdonald , I. Lavenir , H. J. Garringer , E. Gelpi , K. L. Newell , G. G. Kovacs , R. Vidal , B. Ghetti , B. Ryskeldi-Falcon , S. H. W. Scheres , M. Goedert , Science 2022, 375, 167–172. 35025654 10.1126/science.abm7285 PMC7612234

[32] Y. Yang , W. Zhang , A. G. Murzin , M. Schweighauser , M. Huang , S. Lövestam , S. Y. Peak-Chew , T. Saito , T. C. Saido , J. Macdonald , I. Lavenir , B. Ghetti , C. Graff , A. Kumar , A. Nordberg , M. Goedert , S. H. W. Scheres , Acta Neuropathol. 2023, 145, 325–333. 36611124 10.1007/s00401-022-02533-1 PMC9925504

[33] Y. Yang , A. G. Murzin , S. Peak-Chew , C. Franco , H. J. Garringer , K. L. Newell , B. Ghetti , M. Goedert , S. H. W. Scheres , Acta Neuropathol. Commun. 2023, 11, 191. 38049918 10.1186/s40478-023-01694-8 PMC10694933

[34] M. Zielinski , F. S. Peralta Reyes , L. Gremer , S. Schemmert , B. Frieg , L. U. Schäfer , A. Willuweit , L. Donner , M. Elvers , L. N. G. Nilsson , S. Syvänen , D. Sehlin , M. Ingelsson , D. Willbold , G. F. Schröder , Nat. Neurosci. 2023, 26, 2073–2080. 37973869 10.1038/s41593-023-01484-4 PMC10689242

[35] S. H. W. Scheres , B. Ryskeldi-Falcon , M. Goedert , Nature 2023, 621, 701–710. 37758888 10.1038/s41586-023-06437-2

[36] O. Philipson , A. Lord , M. Lalowski , R. Soliymani , M. Baumann , J. Thyberg , N. Bogdanovic , T. Olofsson , L. O. Tjernberg , M. Ingelsson , L. Lannfelt , H. Kalimo , L. N. G. Nilsson , Neurobiol. Aging 2012, 33, 1010.e1–13. 10.1016/j.neurobiolaging.2011.10.022 22118948

[37] M. Schöll , A. Wall , S. Thordardottir , D. Ferreira , N. Bogdanovic , B. Långström , O. Almkvist , C. Graff , A. Nordberg , Neurology 2012, 79, 229–236. 22700814 10.1212/WNL.0b013e31825fdf18

[38] H. Kalimo , M. Lalowski , N. Bogdanovic , O. Philipson , T. D. Bird , D. Nochlin , G. D. Schellenberg , R. Brundin , T. Olofsson , R. Soliymani , M. Baumann , O. Wirths , T. A. Bayer , L. N. O. Nilsson , H. Basun , L. Lannfelt , M. Ingelsson , Acta. Neuropathol. Commun. 2013, 1, 60. 24252272 10.1186/2051-5960-1-60 PMC4226306

[39] M. D. Ikonomovic , W. E. Klunk , E. E. Abrahamson , C. A. Mathis , J. C. Price , N. D. Tsopelas , B. J. Lopresti , S. Ziolko , W. Bi , W. R. Paljug , M. L. Debnath , C. E. Hope , B. A. Isanski , R. L. Hamilton , S. T. DeKosky , Brain 2008, 131, 1630–1645. 18339640 10.1093/brain/awn016 PMC2408940

[40] R. F. Rosen , B. J. Ciliax , T. S. Wingo , M. Gearing , J. Dooyema , J. J. Lah , J. A. Ghiso , H. Le Vinerd , L. C. Walker , Acta Neuropathol. 2010, 119, 221–233. 19690877 10.1007/s00401-009-0583-3 PMC3045810

[41] E. E. Abrahamson , J. K. Kofler , C. R. Becker , J. C. Price , K. L. Newell , B. Ghetti , J. R. Murrell , C. A. McLean , O. L. Lopez , C. A. Mathis , W. E. Klunk , V. L. Villemagne , M. D. Ikonomovic , Brain 2021, 145, 2161–2176. 10.1093/brain/awab434 PMC9630719 34918018

[42] A. Åslund , C. J. Sigurdson , T. Klingstedt , S. Grathwohl , T. Bolmont , D. L. Dickstein , E. Glimsdal , S. Prokop , M. Lindgren , P. Konradsson , D. M. Holtzman , P. R. Hof , F. L. Heppner , S. Gandy , M. Jucker , A. Aguzzi , P. Hammarström , K. P. R. Nilsson , ACS Chem. Biol. 2009, 4, 673–684. 19624097 10.1021/cb900112v PMC2886514

[43] T. Klingstedt , H. Shirani , K. O. A. Åslund , N. J. Cairns , C. J. Sigurdson , M. Goedert , K. P. R. Nilsson , Chem. Eur. J. 2013, 19, 10179–10192. 23780508 10.1002/chem.201301463 PMC3884759

[44] T. Klingstedt , H. Shirani , J. Mahler , B. M. Wegenast-Braun , S. Nyström , M. Goedert , M. Jucker , K. P. R. Nilsson , Chemistry 2015, 21, 9072–9082. 26013403 10.1002/chem.201500556 PMC4517144

[45] H. Shirani , M. Linares , C. J. Sigurdson , M. Lindgren , P. Norman , K. P. R. Nilsson , Chemistry 2015, 21, 15133–15137. 26388448 10.1002/chem.201502999 PMC4641461

[46] L. Björk , M. Bäck , L. Lantz , B. Ghetti , R. Vidal , T. Klingstedt , K. P. R. Nilsson , Chemistry 2022, 28, e202201557. 35950816 10.1002/chem.202201557 PMC9643645

[47] L. Lantz , H. Shirani , B. Ghetti , R. Vidal , T. Klingstedt , K. P. R. Nilsson , Chem. Eur. J. 2023, 29, e202203568. 36645413 10.1002/chem.202203568 PMC10101888

[48] L. Björk , T. Klingstedt , K. P. R. Nilsson , ChemBioChem 2023, 24, e202300044. 36891883 10.1002/cbic.202300044 PMC10404026

[49] H. Shirani , H. Appelqvist , M. Bäck , T. Klingstedt , N. J. Cairns , K. P. R. Nilsson , Chemistry 2017, 23, 17127–17135. 28926133 10.1002/chem.201703846 PMC5928317

[50] T. Klingstedt , H. Shirani , B. Ghetti , R. Vidal , K. P. R. Nilsson , ChemBioChem 2021, 22, 2568–2581. 34101954 10.1002/cbic.202100199 PMC8409278

[51] L. Björk , H. Shirani , Y. Todarwal , M. Linares , R. Vidal , B. Ghetti , P. Norman , T. Klingstedt , K. P. R. Nilsson , Eur. J. Org. Chem. 2023, 26, e202300583. 10.1002/ejoc.202300583 PMC10997339 38585413

[52] T. Klingstedt , L. Lantz , H. Shirani , J. Ge , J. Hanrieder , R. Vidal , B. Ghetti , K. P. R. Nilsson , ACS Chem. Neurosci. 2024, 15, 1581–1595. 38523263 10.1021/acschemneuro.4c00021 PMC10995944

[53] I. Baghallab , J. M. Reyes-Ruiz , K. Abulnaja , E. Huwait , C. Glabe , J. Alzheimer′s Dis. 2018, 66, 1234–1244. 10.3233/JAD-180582 PMC6294585 30412489

[54] H. Englund , D. Sehlin , A. S. Johansson , L. N. Nilsson , P. Gellerfors , S. Paulie , L. Lannfelt , F. E. Pettersson , J. Neurochem. 2007, 103, 334–345. 17623042 10.1111/j.1471-4159.2007.04759.x

[55] H. Kroth , F. Oden , J. Molette , H. Schieferstein , F. Capotosti , A. Mueller , M. Berndt , H. Schmitt-Willich , V. Darmency , E. Gabellieri , C. Boudou , T. Juergens , Y. Varisco , E. Vokali , D. T. Hickman , G. Tamagnan , A. Pfeifer , L. Dinkelborg , A. Muhs , A. Stephens , Eur. J. Nucl. Med. Mol. Imaging 2019, 46, 2178–2189. 31264169 10.1007/s00259-019-04397-2 PMC6667408

[56] H. Kroth , F. Oden , A. M. Serra , J. Molette , A. Mueller , M. Berndt , F. Capotosti , E. Gabellieri , H. Schmitt-Willich , D. Hickman , A. Pfeifer , L. Dinkelborg , A. Stephens , Bioorg. Med. Chem. 2021, 52, 116528. 34839158 10.1016/j.bmc.2021.116528

[57] C. A. Mathis , Y. Wang , D. P. Holt , G.-F. Huang , M. L. Debnath , W. E. Klunk , J. Med. Chem. 2003, 46, 2740–2754. 12801237 10.1021/jm030026b

[58] C. H. van Dyck , C. J. Swanson , P. Aisen , R. J. Bateman , C. Chen , M. Gee , M. Kanekiyo , D. Li , L. Reyderman , S. Cohen , L. Froelich , S. Katayama , M. Sabbagh , B. Vellas , D. Watson , S. Dhadda , M. Irizarry , L. D. Kramer , T. Iwatsubo , N. Engl. J. Med. 2023, 388, 9–21. 36449413 10.1056/NEJMoa2212948

