# Supplementary material for: Distinct Chemical Determinants are Essential for Achieving Ligands for Superior Optical Detection of Specific Amyloid‐β Deposits in Alzheimer's Disease
Source: ChemistryOpen. 2024 Nov 7;13(12):e202400186. doi: 10.1002/open.202400186 (PMC11625938; doi:10.1002/open.202400186)
Supplement: Supplementary file 1 — Supporting Information [file OPEN-13-e202400186-s001.pdf]

ChemistryOpen

Supporting Information

# Distinct Chemical Determinants are Essential for Achieving Ligands for Superior Optical Detection of Specific Amyloid-β Deposits in Alzheimer's Disease

Xiongyu Wu, Hamid Shirani, Ruben Vidal, Bernardino Ghetti, Martin Ingelsson, Therése Klingstedt, and K. Peter R. Nilsson*

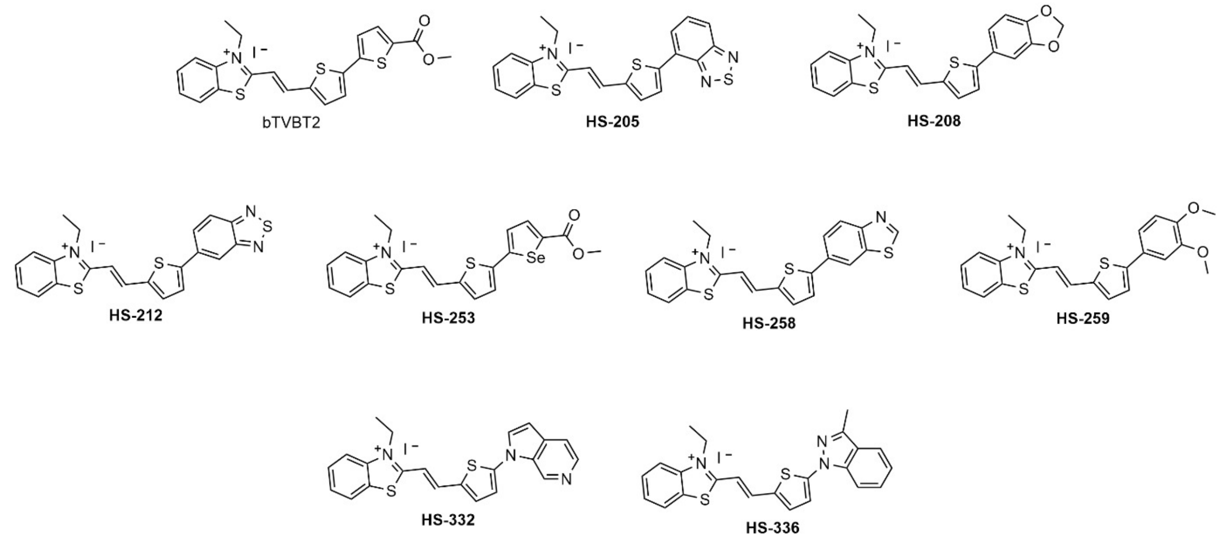


**Figure S1.** Chemical structure of TVB based ligands with different terminal heterocyclic moieties.

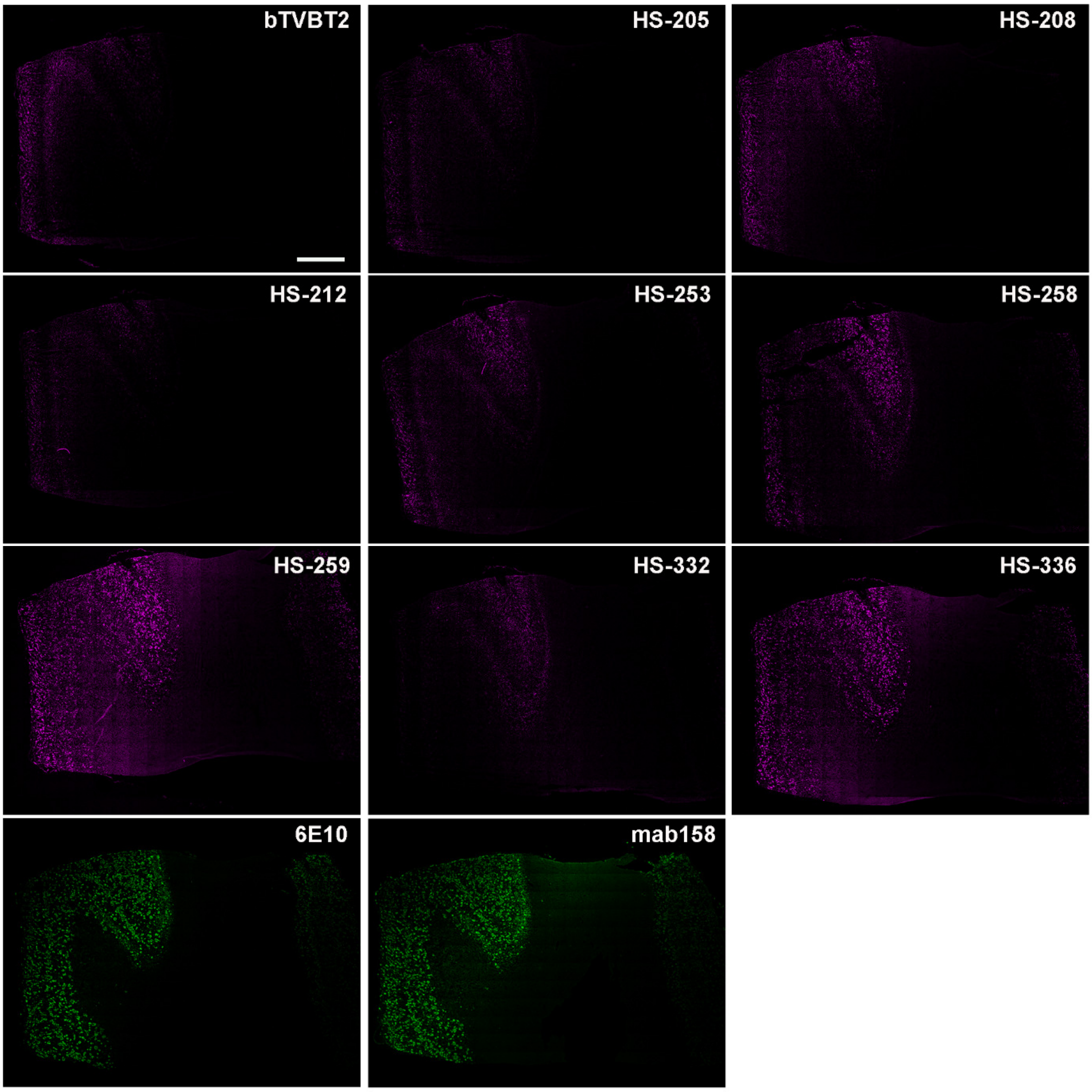


**Figure S2.** Overview tile fluorescence images of brain tissue sections from a case of diAD associated with the Arctic *APP E693G* mutation stained with the ligands bTVBT, HS-205, HS-208, HS-212, HS-253, HS-258, HS-259, HS-332 or HS-336, as well as the antibodies 6E10 or mab158. The fluorescence from the respective ligand is shown in magenta, whereas antibody staining is shown in green. Scale bar represents 2 mm.

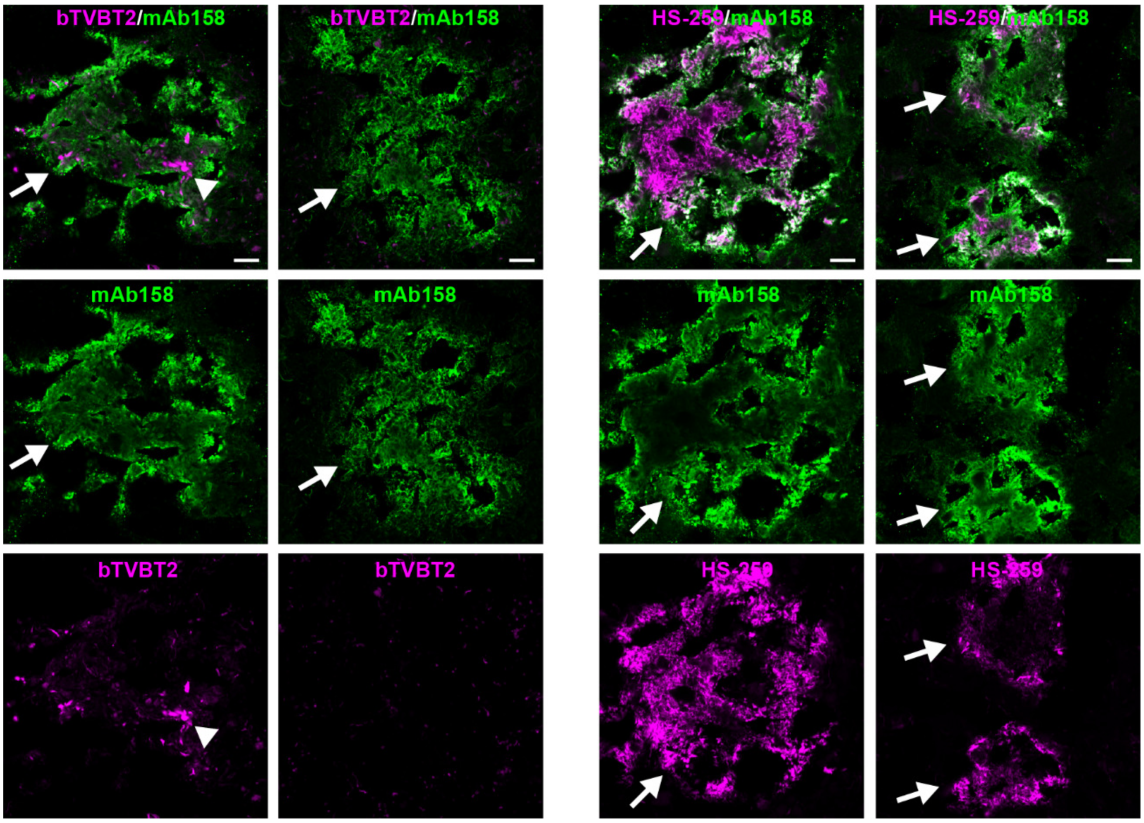


**Figure S3.** Images of a tissue sections co-stained with bTVBT2 (magenta, left panel) or HS-259 (magenta, right panel) and the antibody mab158. Aβ deposits are indicated by white arrows, whereas tau aggregates are indicated by white arrow heads Scale bar represents 20 μm.

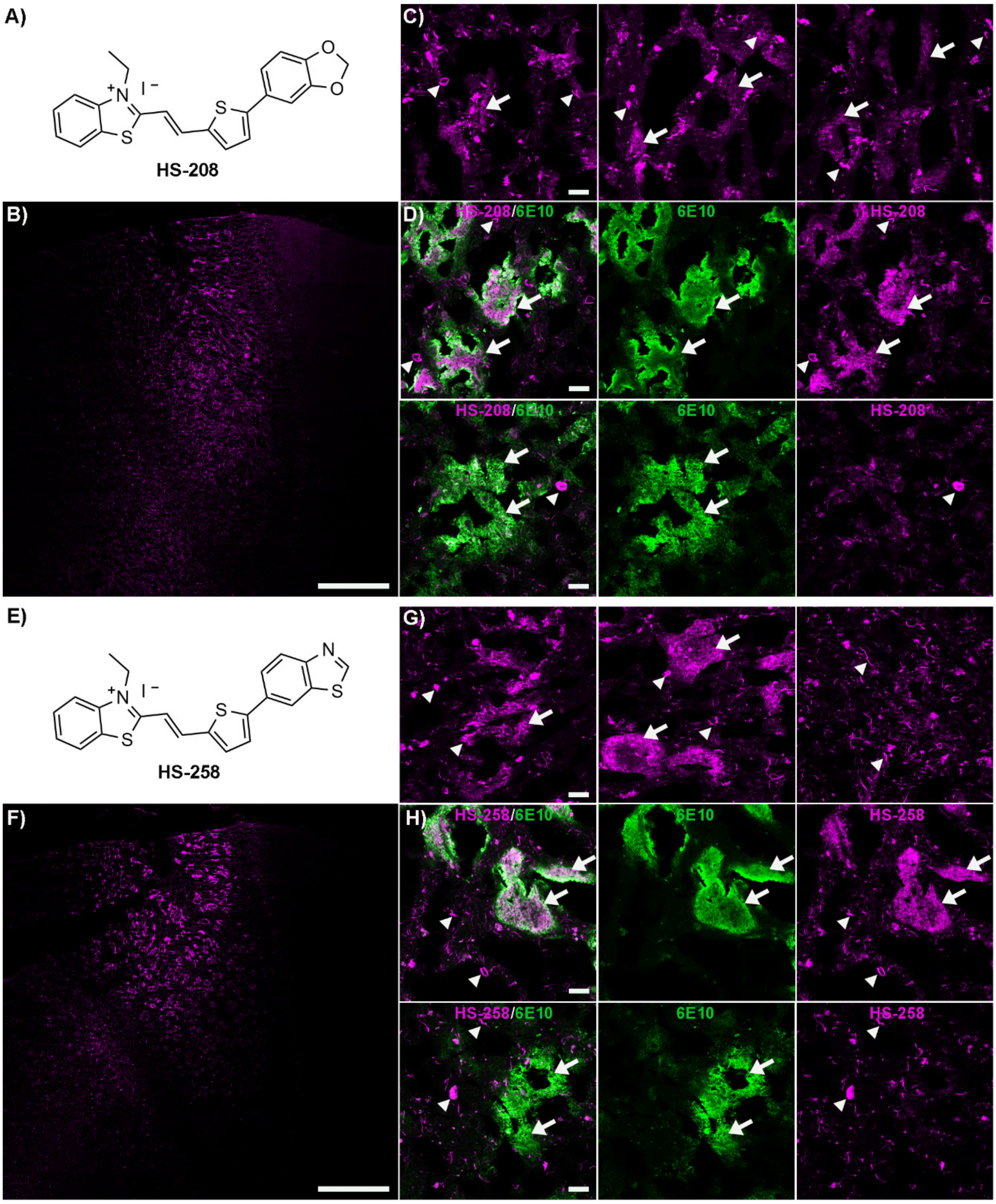


**Figure S4. Staining of human brain tissue sections (frontal cortex) from an individual with diAD caused by the Arctic *APP E693G* mutation with HS-208 or HS-258. A)** Chemical structure of HS-208**. B)** Overview tile image of a brain tissue section from an individual with diAD caused by the Arctic *APP E693G* mutation stained with HS-208. **C)** Images of protein deposits stained with HS-208. **D)** Images of a tissue section co-stained with HS-208 (magenta) and the Aβ antibody 6E10 (green). **E)** Chemical structure of HS-258. F) Overview tile image of a brain tissue section from an individual with diAD caused by the Arctic *APP E693G* mutation stained with HS-258. **G)** Images of different protein deposits stained with HS-258. H) Images of a tissue section co-stained with HS-258 (magenta) and the Aβ antibody 6E10 (green). In C, D, G and H, Aβ-deposits are indicated by white arrows, whereas tau aggregates are indicated by white arrow heads. Scale bars represent 1 mm (B and F) and 20 µm (C, D, G and H).

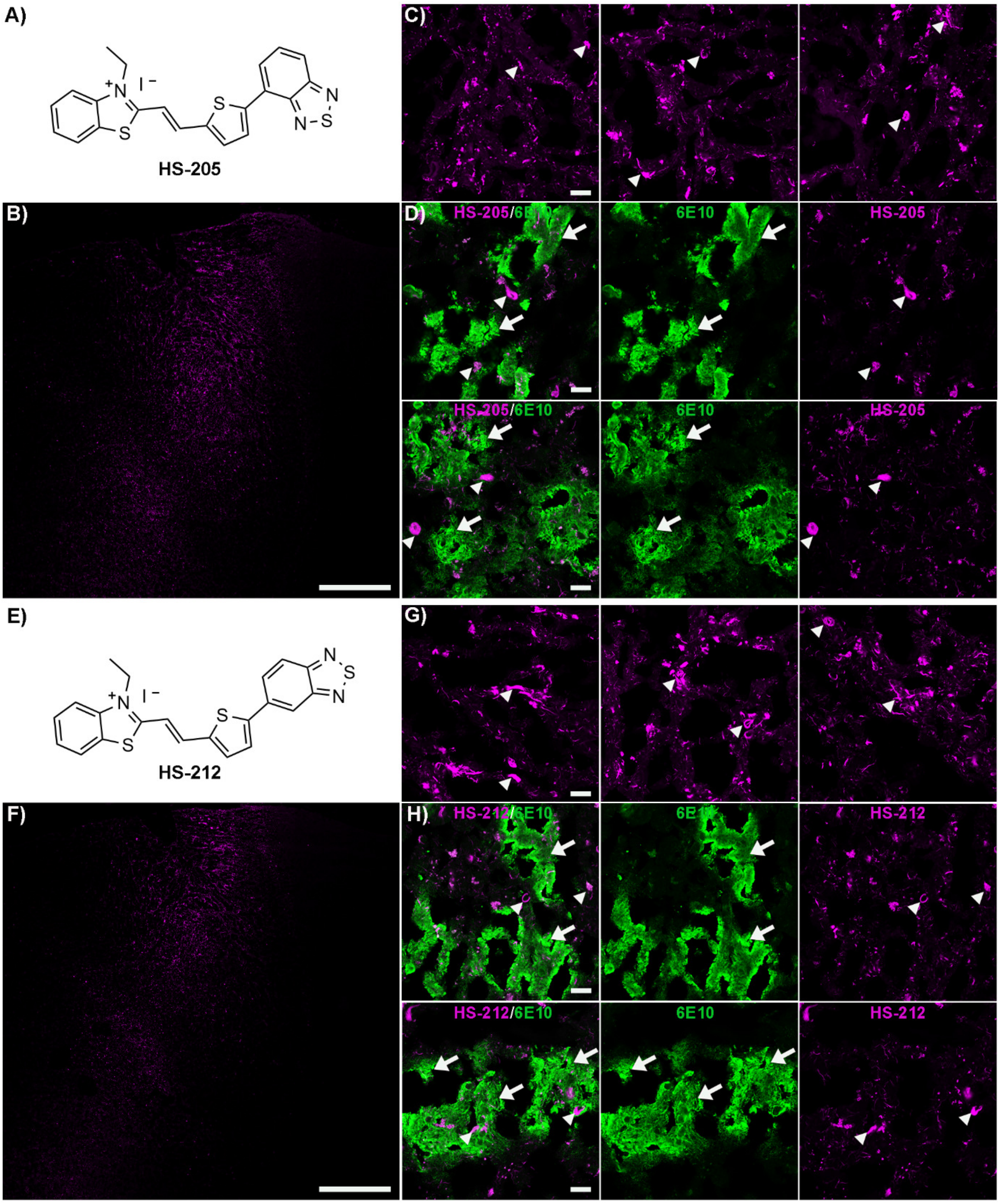


**Figure S5. Staining of human brain tissue sections (frontal cortex) from an individual with diAD caused by the Arctic *APP E693G* mutation with HS-205 or HS-212. A)** Chemical structure of HS-205**. B)** Overview tile image of a brain tissue section from an individual with diAD caused by the Arctic *APP E693G* mutation stained with HS-205. **C)** Images of protein deposits stained with HS-205. **D)** Images of a tissue section co-stained with HS-205 (magenta) and the Aβ antibody 6E10 (green). **E)** Chemical structure of HS-212. F) Overview tile image of a brain tissue section from an individual with diAD caused by the Arctic *APP E693G* mutation stained with HS-212. **G)** Images of different protein deposits stained with HS-212. H) Images of a tissue section co-stained with HS-212 (magenta) and the Aβ antibody 6E10 (green). In C, D, G and H, Aβ-deposits are indicated by white arrows, whereas tau aggregates are indicated by white arrow heads. Scale bars represent 1 mm (B and F) and 20 µm (C, D, G and H).

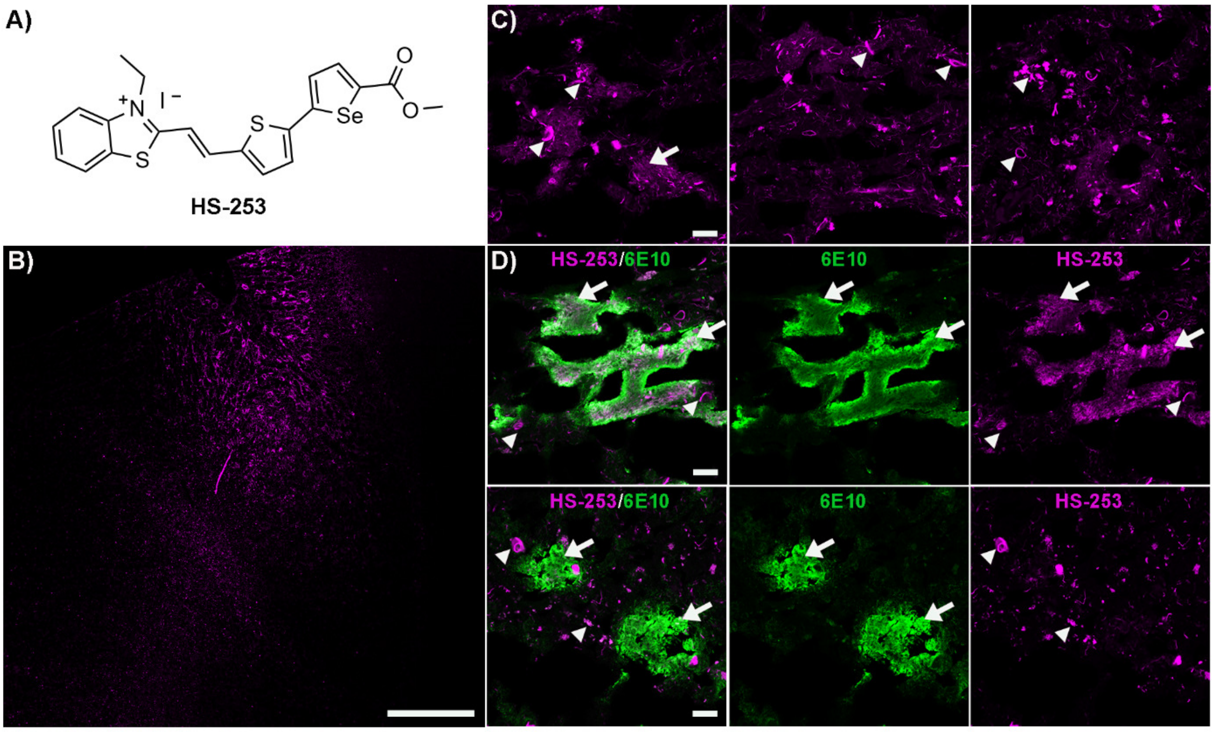


**Figure S6. Staining of human brain tissue sections (frontal cortex) from an individual with diAD caused by the Arctic *APP E693G* mutation with HS-253. A)** Chemical structure of HS-253**. B)** Overview tile image of a brain tissue section from an individual with diAD caused by the Arctic *APP E693G* mutation stained with HS-253. **C)** Images of protein deposits stained with HS-253. **D)** Images of a tissue section co-stained by HS-253 (magenta) and the Aβ antibody 6E10 (green). In C and D, Aβ-deposits are indicated by white arrows, whereas tau aggregates are indicated by white arrow heads. Scale bars represent 1 mm (B and F) and 20 μm (C, D, G and H).

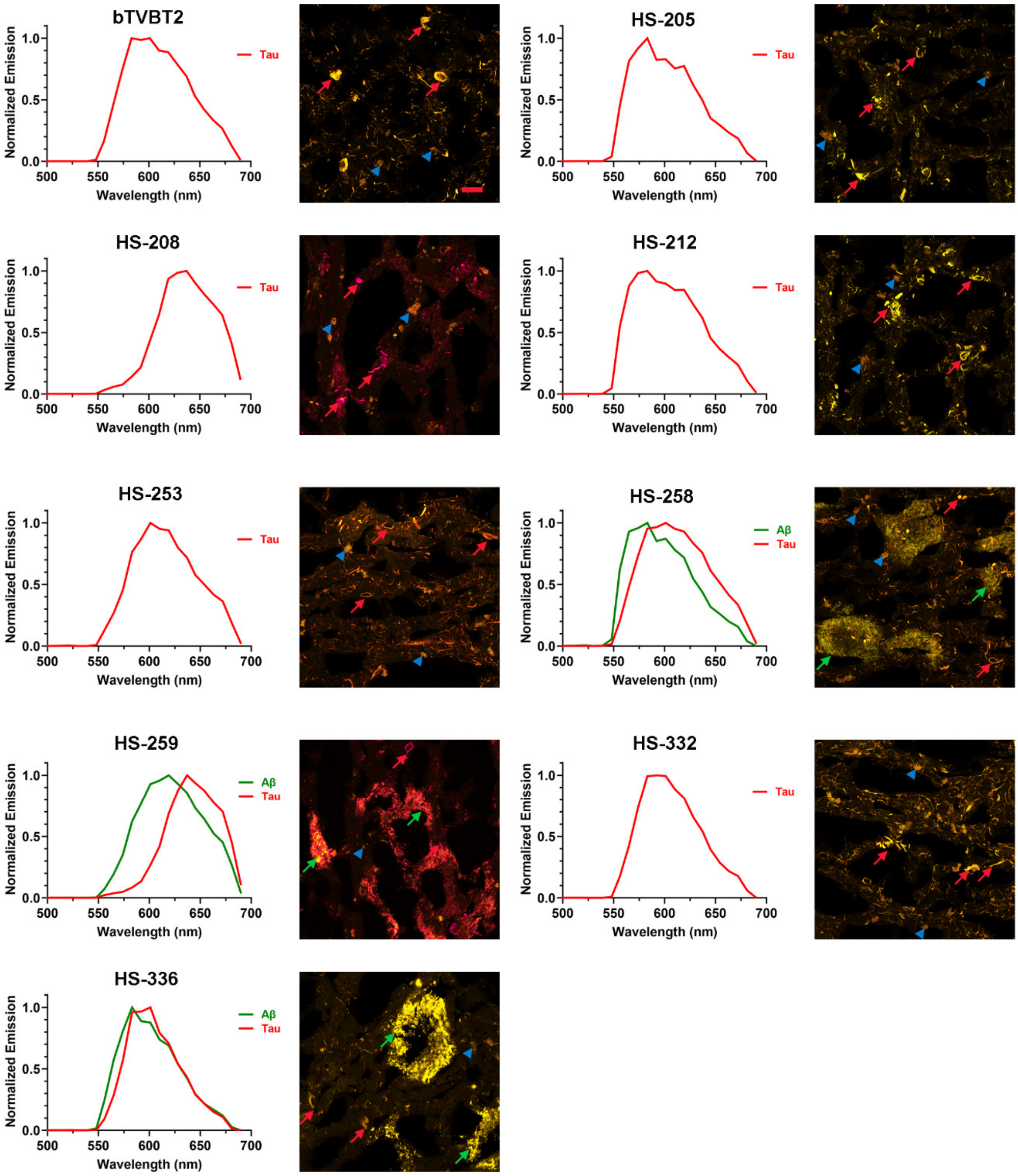


**Figure S7. Spectral assignment of Aβ and tau deposits in a brain tissue section from an individual with diAD associated with the Artic *APP E693G* mutation stained with TVB based ligands.** The emission spectrum (left) of the respective ligand when bound to Aβ deposits (green) or tau aggregates (red) and spectral image (right) of protein aggregates when stained with the respective ligand. Aβ aggregates are indicated by green arrows, whereas tau aggregates are highlighted by red arrows. Granular auto-fluorescent lipofuscin is indicated with blue arrow heads. Scale bar represents 20 µm.

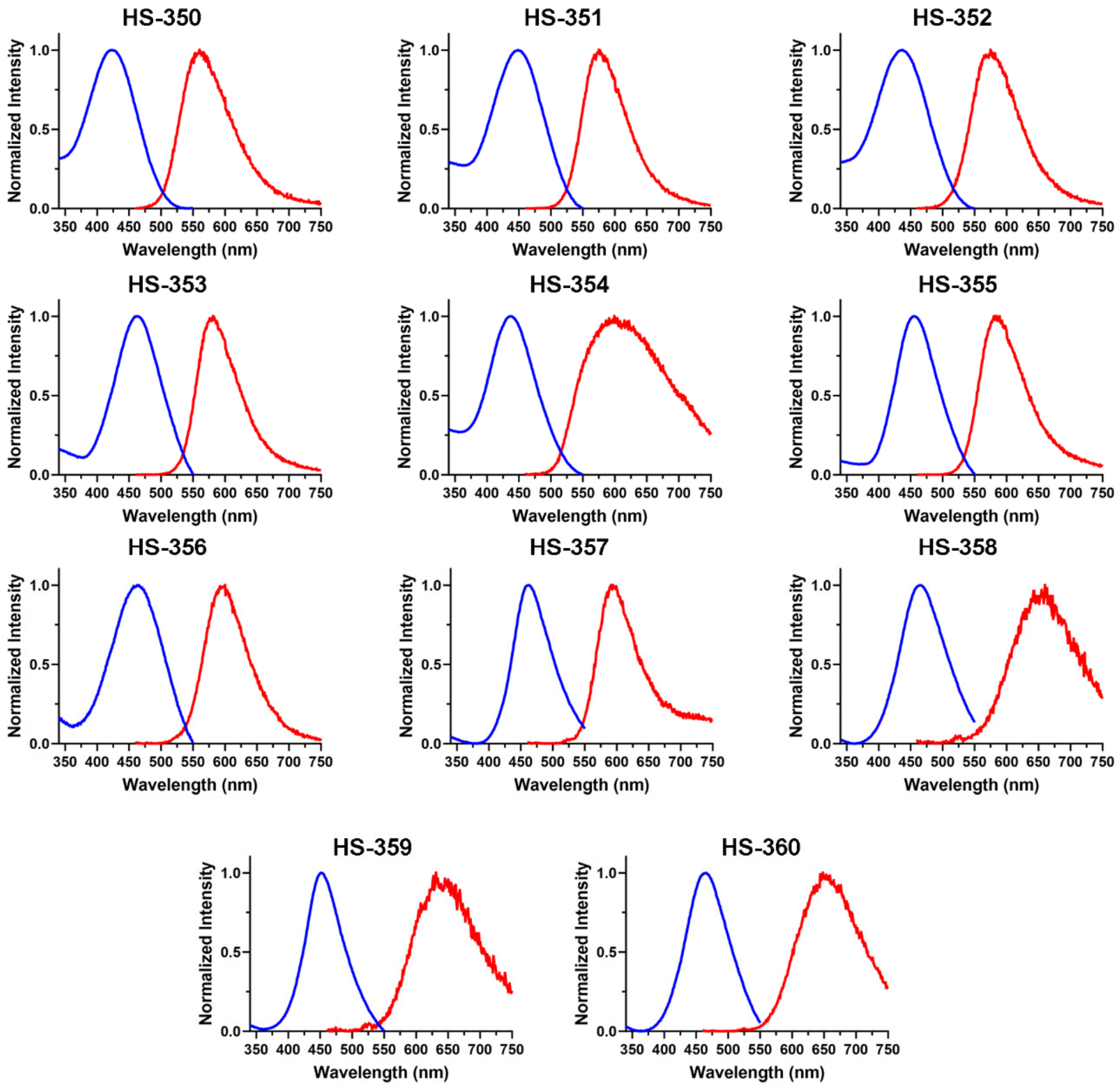


**Figure S8. Optical characteristics of TVB based ligands.** The absorption- (blue) and emission (red) spectrum from the respective ligand (30 μM) in phosphate buffered saline (PBS) pH 7.4.

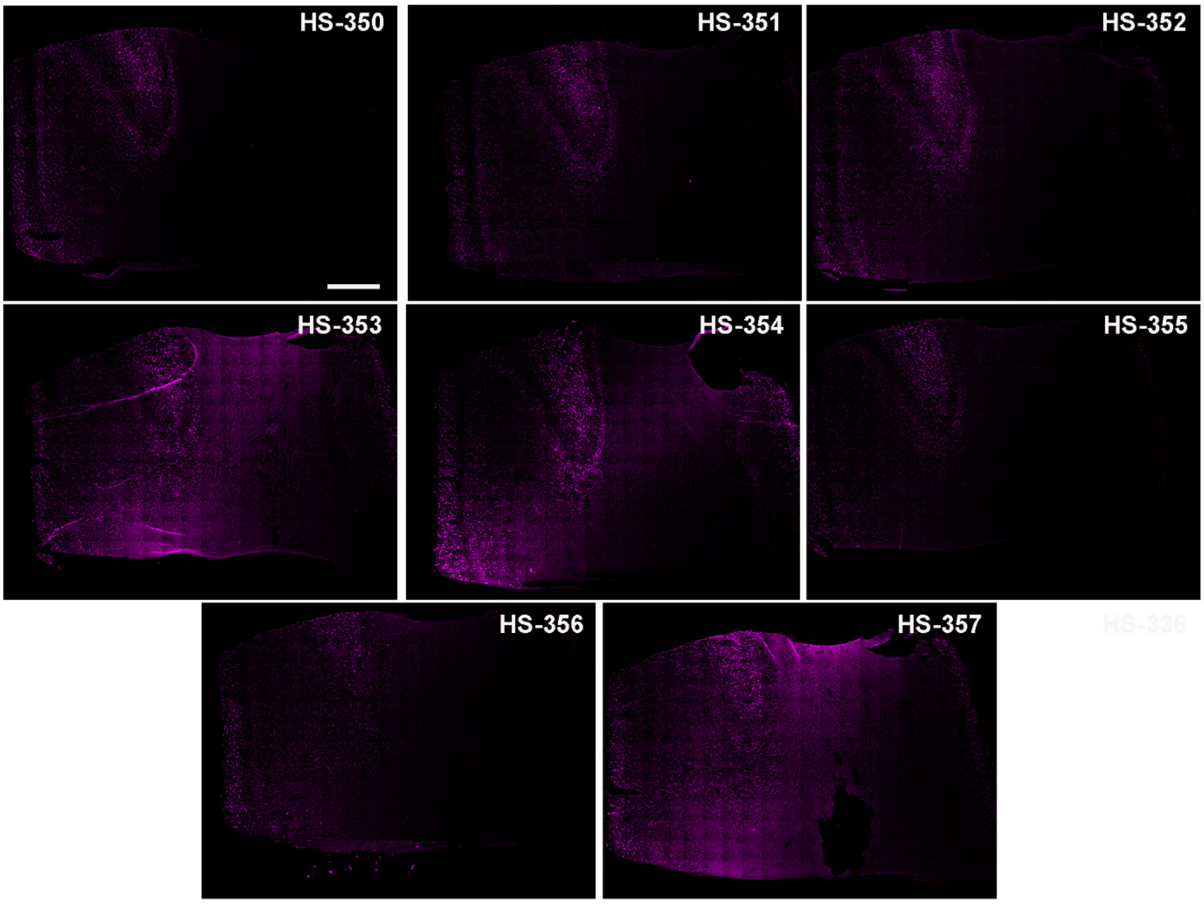


**Figure S9.** Overview tile fluorescence images of brain tissue sections from a case of diAD associated with the Arctic *APP E693G* mutation stained with the ligands HS-350, HS-351, HS-352, HS-353, HS-354, HS-355, HS-356 or HS-357. The fluorescence from the ligand is shown in magenta. Scale bar represents 2 mm.

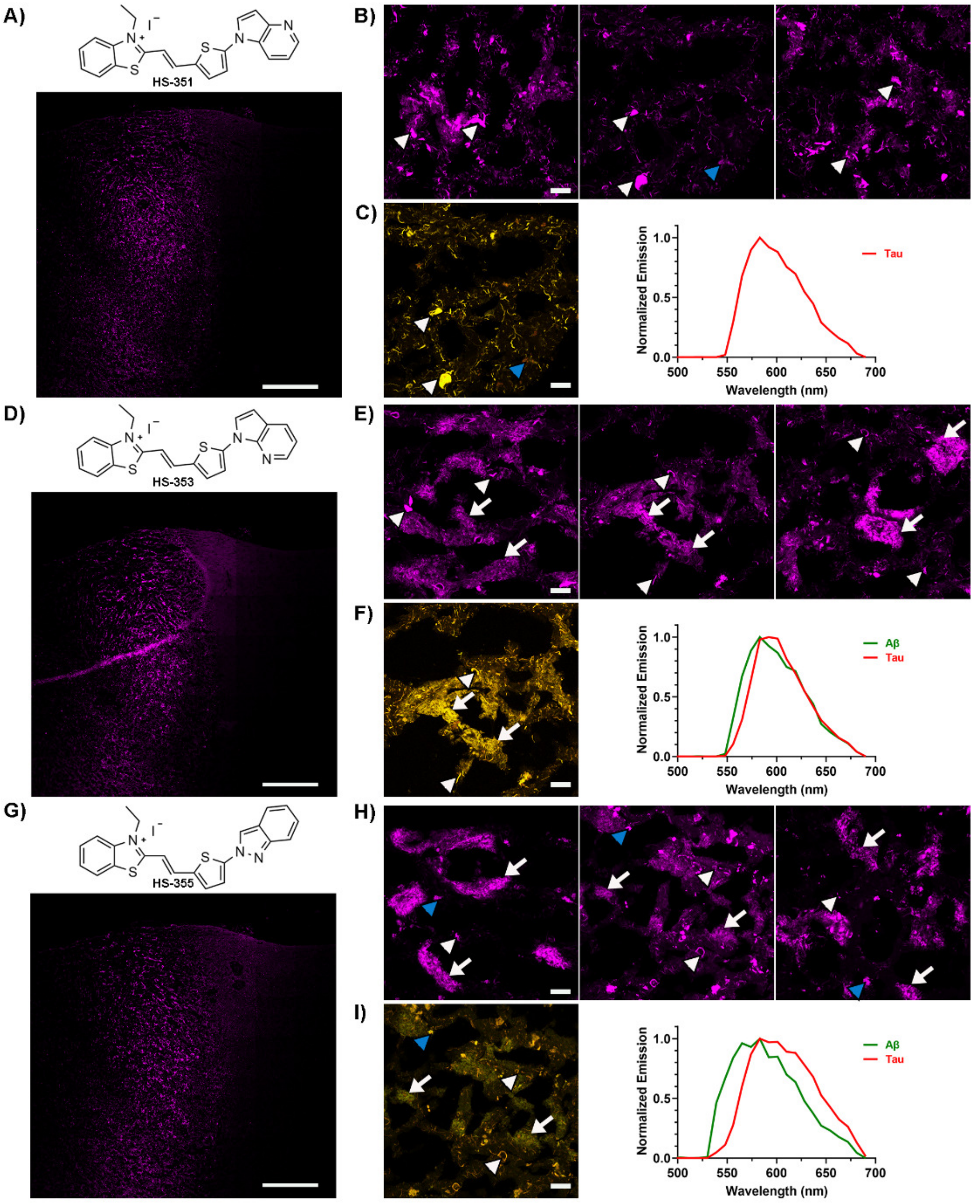


**Figure S10. Staining of human brain tissue sections (frontal cortex) from an individual with diAD caused by the Arctic *APP E693G* mutation with HS-351, HS-353 or HS-355. A)** Chemical structure of HS-351 and overview tile image of a brain tissue section from an individual with diAD caused by the Arctic *APP E693G* mutation stained by HS-351. **B)** Images of protein deposits stained with HS-351. **C)** Spectral image and the emission spectrum from HS-351 when bound to tau aggregates (red). **D)** Chemical structure of HS-353 and overview tile image of a brain tissue section from an individual with diAD caused by the Arctic *APP E693G* mutation stained by HS-353. **E)** Images of protein deposits stained with HS-353. **F)** Spectral image and the emission spectrum from HS-353 when bound to Aβ deposits (green) or tau aggregates (red). **G)** Chemical structure of HS-355 and overview tile image of a brain tissue section from an individual with diAD caused by the Arctic *APP E693G* mutation stained with HS-355. **H)** Images of protein deposits stained with HS-355. **I)** Spectral image and the emission spectrum from HS-355 bound to Aβ deposits (green) or tau aggregates (red). In B, C, E, F, H and I, Aβ-deposits are indicated by white arrows, whereas tau aggregates are indicated by white arrow heads and autofluorescence from granular lipofuscin is indicated by blue arrow heads. Scale bars represent 1 mm (A, D and G) and 20 μm (B, C, E, F, H and I).

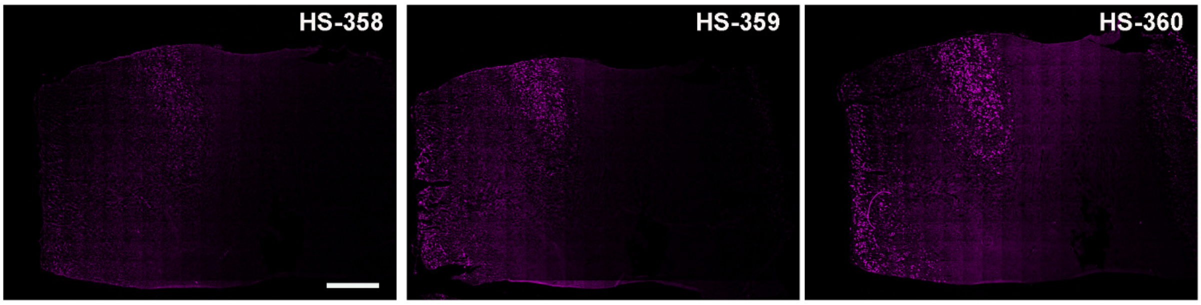


**Figure S11.** Overview tile fluorescence images of brain tissue sections from a case of diAD associated with the Arctic *APP E693G* mutation stained with the ligands HS-358, HS-359 or HS-360. The fluorescence from the ligand is shown in magenta. Scale bar represents 2 mm.

**Table S1. Absorption- and emission maximum for TVB based ligands in PBS.**

| Ligand | $Abs_{max}$ (nm) | $Em_{max}$ (nm) |
|---|---|---|
| HS-350 | 422 | 560 |
| HS-351 | 448 | 575 |
| HS-352 | 437 | 575 |
| HS-353 | 462 | 581 |
| HS-354 | 438 | 599 |
| HS-355 | 455 | 583 |
| HS-356 | 465 | 600 |
| HS-357 | 462 | 594 |
| HS-358 | 465 | 660 |
| HS-359 | 452 | 631 |
| HS-360 | 465 | 648 |

**Table S2. List of the human brain tissue sections used in the study.**

| Case no | Pathology | Brain area | Sex | Age | APOE |
|---|---|---|---|---|---|
| 1 | sAD | Frontal ctx | F | 82 | 4/4 |
| 2 | *APP E693E* | Frontal ctx | Male | 64 | 3/3 |

## Experimental details

*General information:*

TLC was performed using 0.25 mm precoated silica-gel plates (Merck 60 F254), detection by UV-abs at 254 nm. $^{1}H$ (500 MHz) and $^{13}C$-NMR (126 MHz) spectra were recorded on a Bruker NMR instrument (25 °C in $CDCl_3$ or DMSO-d6 or acetone-d6). HPLC-MS was performed on a Waters system (Column: XSELECT Phenyl-Hexyl, 5 µm, 250 x 19 mm and Waters X-Bridge C-18 3.5 µm, 50 x 4.6 mm for preparative and analytical experiments respectively; Mobile phase: organic phase: acetonitrile:water 90:10, with 10 mM $NH_4OAc$; water phase: acetonitrile:water 5:95, with 10 mM $NH_4OAc$. Flash chromatography was performed using the following silica gel: High purity grade (Merck Grade 9385), pore size 60 Å, 230-240 mesh particle size. Some synthesized starting materials of **3a**-**h** or **7b**-**d** contained trace amount of solvent or other impurities which were not needed for further purification. High resolution MS was measured using Agilent Technologies 6550 i Funnel LC-QTOF-MS.

*Compound synthesis:*

General procedure A: Synthesis of **3a**-**h**.

The mixture of **1** (2 mmol), **2a**-**g** (2 mmol), CuI (0.4 mmol), $Cs_2CO_3$ (4 mmol) and DMF (10.0 mL) in a 10-20 mL microwave vial was flushed with $N_2$ gas for about 1 minute, then the vial was sealed and irradiated under microwave for 25 minutes at 170 °C. The reaction mixture was purified using silica gel column chromatography with ethyl acetate-n-heptane (EtOAc-n-heptane) or preparative HPLC with B:A to give the product **3a**-**h**.

General procedure B: Synthesis of **HS-350** to **HS-360**.

The mixture of **3a**-**h** or **7b**-**d** (0.0438-0.132 mmol), **4** (1.0 equivalent) and pyridine (6.0 equivalents) in dry MeOH (1 mL) was stirred at 65 °C overnight. The reaction mixture was filtered and the solid was washed with a few drops of MeOH, acetonitrile and DCM to give **HS-350** to **HS-360** respectively.

General procedure C: Synthesis of **7a** and **7c**-**d**.

The mixture of boronic acid **5** (2.0 equivalents), **6a** or **6c**-**d** (0.4 mmol), PEPPSI$^{TM}$-IPr (0.05 equivalent) and $K_2CO_3$ (4.0 equivalents) in dioxan/MeOH (3:2, 5mL) was flushed with $N_2$ gas for about 30 seconds. The vial was sealed, and the reaction mixture was heated at 70 °C for about 5 hours. The reaction mixture was carefully transferred to a round bottom flask and concentrated to give the crude product. The crude **7a** was purified using silica gel column chromatography with EtOAc-n-heptane (25:75 to 60:40) to give the product **7a** (95 mg, 96% yield). The crude **7c** or **7d** was mixed with MeOH (about 3-4 mL), filtered, and washed with water (10 mL) and a few drops of methanol to give a pure product, which was used for the condensation reaction without necessity for further purification.

Synthesis of **3a**

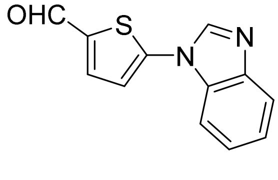


The compound was synthesized according to the general procedure A in 34% yield (155 mg), which was purified using EtOAc-n-heptane (50:50 to 70:30). $^{1}H$-NMR ($CDCl_3$, 500 MHz): δ 9.93 (s, 1H), 8.16 (s, 1H), 7.87 (dd, $J$ = 7.0, 1.5 Hz, 1H), 7.79 (d, $J$ = 4.0 Hz, 1H), 7.71 (dd, $J$ = 7.5,

1.5 Hz, 1H), 7.44-7.37 (m, 2H), 7.28 (d, *J* = 4.0 Hz, 1H). $^{13}$C-NMR ($CDCl_3$, 126 MHz): δ 182.8, 146.4, 144.2, 142.1, 139.6, 136.1, 133.3, 125.0, 124.1, 121.2, 120.0, 110.8. LCMS (ESI$^+$): m/z calcd for $C_{12}H_9N_2OS$ $(M+H)^+$: 229.0. Found: 229.5.

Synthesis of **3b**

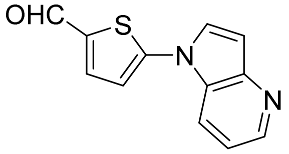


The compound was synthesized according to the general procedure A in 17% yield (78 mg), which was purified using EtOAc-n-heptane (30:70 to 70:30). $^1$H-NMR ($CDCl_3$, 500 MHz): δ 9.85 (s, 1H), 8.54 (dd, *J* = 4.5, 1.0 Hz, 1H), 8.03 (d, *J* = 8.0 Hz, 1H), 7.73 (d, *J* = 4.0 Hz, 1H), 7.59 (d, *J* = 3.5 Hz, 1H), 7.22 (dd, *J* = 8.5, 4.5 Hz, 1H), 7.14 (d, *J* = 4.0 Hz, 1H), 6.92 (d, *J* = 3.5 Hz, 1H). $^{13}$C-NMR ($CDCl_3$, 126 MHz): δ 182.6, 150.2, 148.2, 145.2, 137.7, 136.5, 131.0, 129.0, 118.4, 118.1, 107.7. LCMS (ESI$^+$): m/z calcd for $C_{12}H_9N_2OS$ $(M+H)^+$: 229.0. Found: 229.3.

Synthesis of **3c**

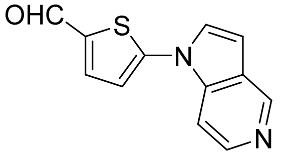


The compound was synthesized according to the general procedure A in 4% yield (42 mg), which was purified using EtOAc-n-heptane (50:50 to 80:20). $^1$H-NMR ($CDCl_3$, 500 MHz): δ 9.89 (s, 1H), 8.98 (s, 1H), 8.47 (d, *J* = 6.0 Hz, 1H), 7.76 (d, *J* = 4.0 Hz, 1H), 7.65 (d, *J* = 6.0 Hz, 1H), 7.40 (d, *J* = 3.5 Hz, 1H), 7.20 (d, *J* = 4.0 Hz, 1H), 6.84 (dd, *J* = 3.5, 0.5 Hz, 1H).$^{13}$C-NMR ($CDCl_3$, 126 MHz): δ 182.7, 149.6, 144.6, 143.3, 139.7, 138.4, 136.4, 129.0, 126.4, 119.0, 106.2, 105.8. LCMS (ESI$^+$): m/z calcd for $C_{12}H_9N_2OS$ $(M+H)^+$: 229.0. Found: 229.4.

Synthesis of **3d**

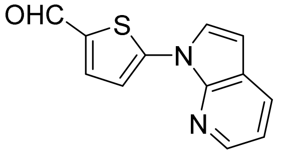


The compound was synthesized according to the general procedure A in 4% yield (19 mg), which was purified using EtOAc-n-heptane (50:50 to 80:20). $^1$H-NMR ($CDCl_3$, 500 MHz): δ 9.86 (s, 1H), 8.45 (dd, *J* = 4.5, 1.5 Hz, 1H), 7.95 (dd, *J* = 8.0, 1.0 Hz, 1H), 7.70 (d, *J* = 4.0 Hz, 1H), 7.59 (d, *J* = 3.5 Hz, 1H), 7.50 (d, *J* = 4.0 Hz, 1H), 7.20 (dd, *J* = 8.0, 5.0 Hz, 1H), 6.69 (d, *J* = 4.0 Hz, 1H). $^{13}$C-NMR ($CDCl_3$, 126 MHz): δ 183.0, 149.3, 147.1, 144.3, 136.4, 136.3, 129.7, 126.3, 122.1, 118.2, 115.2, 104.9. LCMS (ESI$^+$): m/z calcd for $C_{12}H_9N_2OS$ $(M+H)^+$: 229.0. Found: 229.3.

Synthesis of **3e** and **3h**

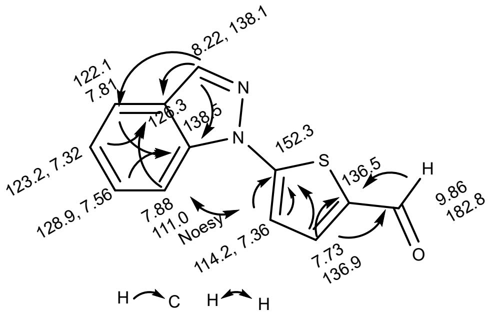


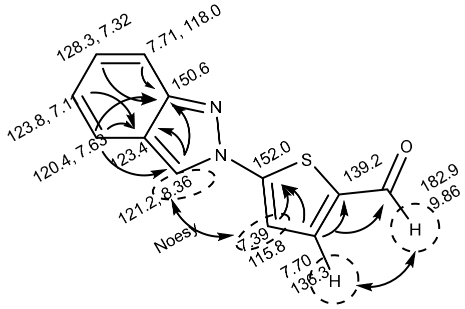


Compound **3e** was synthesized according to the general procedure A in 15% yield (70 mg), which was purified using EtOAc-n-heptane (40:60 to 50:50) and further with preparative liquid chromatography using B:A (20:80 to 100:0). Compound **3h** was also achieved in 6% yield (30 mg) after the purification with preparative LC. The compounds were assigned using 2D-NMR. **3e**: $^{1}$H-NMR ($CDCl_3$, 500 MHz): δ 9.86 (s, 1H), 8.22 (s, 1H), 7.88 (d, $J$ = 9.0, 1H), 7.81 (d, $J$ = 8.0 Hz, 1H), 7.73 (d, $J$ = 4.5 Hz, 1H), 7.56 (dd, $J$ = 8.5, 7.0 Hz, 1H), 7.36 (d, $J$ = 4.5 Hz, 1H), 7.32 (t, $J$ = 7.5 Hz, 1H). $^{13}$C-NMR ($CDCl_3$, 126 MHz): δ 182.8, 152.3, 138.5, 138.1, 136.9, 136.5, 128.9, 126.3, 123.2, 122.1, 114.2, 111.0. LCMS (ESI$^+$): m/z calcd for $C_{12}H_9N_2OS$ $(M+H)^+$: 229.0. Found: 229.3.

**3h**: $^{1}$H-NMR ($CDCl_3$, 500 MHz): δ 9.86 (s, 1H), 8.36 (s, 1H), 7.71 (d, $J$ = 9.0, 1H), 7.70 (d, $J$ = 4.0 Hz, 1H), 7.63 (d, $J$ = 8.5 Hz, 1H), 7.39 (d, $J$ = 4.0 Hz, 1H), 7.32 (t, $J$ = 8.0 Hz, 1H), 7.11 (t, $J$ = 7.5 Hz, 1H). $^{13}$C-NMR ($CDCl_3$, 126 MHz): δ 182.9, 152.0, 150.5, 139.2, 136.3, 128.3, 123.8, 123.4, 121.2, 120.4, 118.0, 115.8. LCMS (ESI$^+$): m/z calcd for $C_{12}H_9N_2OS$ $(M+H)^+$: 229.0. Found: 229.5.

Synthesis of **3f**:

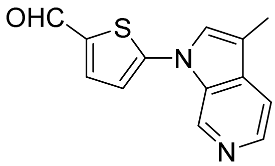


Compound **3f** was synthesized on a 1.89 mmol scale according to the general procedure A in 12% yield (56 mg), which was purified using EtOAc-n-heptane (50:50 to 80:20) and further with preparative liquid chromatography using B:A (20:80 to 100:0). $^{1}$H-NMR ($CDCl_3$, 500 MHz): δ 9.85 (s, 1H), 9.11 (br s, 1H), 8.40 (d, $J$ = 5.0 Hz, 1H), 7.73 (d, $J$ = 4.0 Hz, 1H), 7.51 (d, $J$ = 5.5 Hz, 1H), 7.27 (br d, $J$ = 1.0 Hz, 1H), 7.17 (t, $J$ = 4.5 Hz, 1H), 2.34 (d, $J$ = 1.0 Hz, 3H). $^{13}$C-NMR ($CDCl_3$, 126 MHz): δ 182.6, 150.3, 140.8, 137.2, 136.8, 135.9, 133.7, 133.0, 128.6, 117.5, 115.7, 114.2, 9.3. LCMS (ESI$^+$): m/z calcd for $C_{13}H_{11}N_2OS$ $(M+H)^+$: 243.1. Found: 243.4.

Synthesis of **3g**:

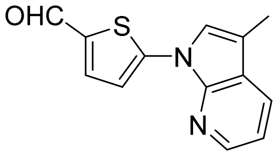


Compound **3g** was synthesized in 15% yield (73 mg) according to the general procedure A, which was purified using EtOAc-n-heptane (50:50 to 80:20) and further with preparative liquid chromatography using B:A (20:80 to 100:0). $^{1}$H-NMR ($CDCl_3$, 500 MHz): δ 9.83 (s, 1H), 8.42 (dd, *J* = 5.0, 1.5 Hz, 1H), 7.87 (dd, *J* = 7.5, 1.5 Hz, 1H), 7.67 (d, *J* = 4.0 Hz, 1H), 7.44 (d, *J* = 4.5 Hz, 1H), 7.34 (br d, *J* = 1.0 Hz, 1H), 7.18 (dd, *J* = 7.5, 5.0 Hz, 1H), 2.32 (d, *J* = 1.0 Hz, 3H). $^{13}$C-NMR ($CDCl_3$, 126 MHz): δ 182.9, 149.6, 147.3, 144.1, 136.6, 135.5, 129.6, 127.9, 125.7, 123.4, 123.1, 117.6, 114.7, 114.2, 9.8. LCMS (ESI$^+$): m/z calcd for $C_{13}H_{11}N_2OS$ $(M+H)^+$: 243.1. Found: 243.2.

Synthesis of **7b**:

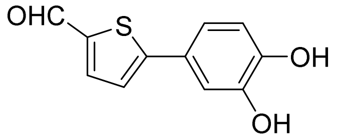


To the solution of **7a** (95 mg, 0.383 mmol) in dry DCM (8 mL) was added 1.0 M BBr3 in hexane (2.3 mL, 2.30 mmol). The resulting mixture was stirred at rt for 3.5 hours. The reaction mixture was quenched with water/acetonitrile (5mL/5mL), another 15 mL water was added, and the mixture was extracted with EtOAc (15 mL x 3). The organic layers were combined, dried over $MgSO_4$, filtered, and concentrated to give the crude **7b**. DCM (1 mL x 2) was added to rinse the crude product, giving **7b** (35.1 mg, 42%), which was used without further purification for the next step. $^{1}$H-NMR (acetone-d6, 500 MHz): δ 9.88 (s, 1H), 7.88 (d, *J* = 4.0 Hz, 1H), 7.44 (d, *J* = 4.0 Hz, 1H), 7.25 (d, *J* = 2.0 Hz, 1H), 7.17 (dd, *J* = 8.0, 2.0 Hz, 1H), 6.91 (d, *J* = 8.5 Hz, 1H). $^{13}$C-NMR (acetone-d6, 126 MHz): δ 183.5, 155.1, 147.9, 146.6, 142.2, 139.3, 126.0, 124.0, 119.4, 116.8, 114.1. LCMS (ESI$^+$): m/z calcd for $C_{11}H_9O_3S$ $(M+H)^+$: 221.0. Found: 221.3.

Synthesis of **7c**:

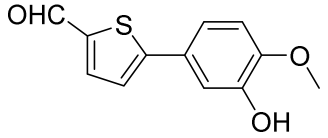


Compound **7c** was synthesized according to the general procedure C in 41% yield (39 mg). $^{1}$H-NMR (DMSO-d6, 500 MHz): δ 9.86 (s, 1H), 7.98 (d, *J* = 3.5 Hz, 1H), 7.55 (d, *J* = 4.0 Hz, 1H), 7.23 (dd, *J* = 8.5, 2.0 Hz, 1H), 7.22 (d, *J* = 2.5 Hz, 1H), 7.01 (d, *J* = 8.5 Hz, 1H), 3.82 (s, 3H). $^{13}$C-NMR (DMSO-d6, 126 MHz): δ 183.7, 153.4, 149.3, 147.2, 140.8, 139.5, 125.3, 123.9, 117.6, 113.2, 112.6, 55.7. LCMS (ESI$^+$): m/z calcd for $C_{12}H_{11}O_3S$ $(M+H)^+$: 235.0. Found: 235.4.

Synthesis of **7d**:

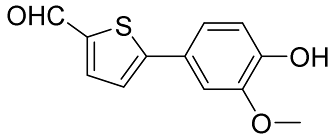


Compound **7d** was synthesized according to the general procedure C in 85% yield (80 mg). $^{1}$H-NMR (DMSO-d6, 500 MHz): δ 9.67 (s, 1H), 7.81 (d, *J* = 4.0 Hz, 1H), 7.25 (d, *J* = 4.0 Hz, 1H), 7.08 (dd, *J* = 8.5, 1.5 Hz, 1H), 6.97 (s, 1H), 6.32 (d, *J* = 8.5 Hz, 1H), 3.70 (s, 3H). $^{13}$C-NMR (DMSO-d6, 126 MHz): δ 181.7, 157.7, 150.6, 140.1, 136.2, 121.7, 119.4, 117.9, 110.0, 55.4. LCMS (ESI$^{+}$): m/z calcd for $C_{12}H_{11}O_3S$ $(M+H)^{+}$: 235.0. Found: 235.3.

Synthesis of **HS-350**:

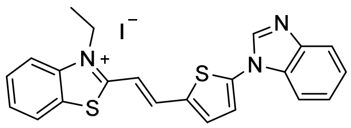


Following the general procedure B, ligand **HS-350** was synthesized in 71% yield (39 mg) on 0.107 mmol scale. $^{1}$H-NMR (DMSO-d6, 500 MHz): δ 8.80 (s, 1H), 8.54 (d, *J* = 15.0 Hz, 1H), 8.45 (d, *J* = 8.0 Hz, 1H), 8.29 (d, *J* = 8.5 Hz, 1H), 8.06 (d, *J* = 4.5 Hz, 1H), 7.91 (d, *J* = 8.0 Hz, 1H), 7.88 (t, *J* = 8.0 Hz, 1H), 7.85 (d, *J* = 8.0 Hz, 1H), 7.79 (t, *J* = 7.5 Hz, 1H), 7.78 (d, *J* = 15.0 Hz, 1H), 7.72 (d, *J* = 4.0 Hz, 1H), 7.49 (t, *J* = 8.0 Hz, 1H), 7.42 (t, *J* = 7.5 Hz, 1H), 4.95 (q, *J* = 7.0 Hz, 2H), 1.48 (t, *J* = 7.0 Hz, 3H). $^{13}$C-NMR (DMSO-d6, 126 MHz): δ 170.8, 143.7, 143.5, 143.4, 141.3, 140.9, 135.5, 134.6, 132.6, 129.6, 128.4, 128.2, 124.6, 123.5, 123.7, 121.2, 120.5, 116.6,111.2, 111.0, 44.4, 14.2. HRMS (ESI$^{+}$): m/z calcd for $C_{22}H_{18}N_3S_2^{+}$ $(M\text{-}I)^{+}$: 388.0942. Found: 388.0946.

Synthesis of **HS-351**:

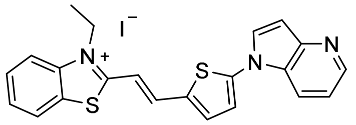


Following the general procedure B, ligand **HS-351** was synthesized in 73% yield (46.2 mg) on 0.123 mmol scale. $^{1}$H-NMR (DMSO-d6, 500 MHz): δ 8.55 (dd, *J* = 5.0, 1.0 Hz, 1H), 8.52 (d, *J* = 15.5 Hz, 1H), 8.43 (d, *J* = 8.0 Hz, 1H), 8.31 (d, *J* = 8.5 Hz, 1H), 8.27 (d, *J* = 8.5 Hz, 1H), 8.20 (d, *J* = 8.5 Hz, 1H), 8.04 (d, *J* = 4.0 Hz, 1H), 7.87 (dt, *J* = 8.0, 0.5 Hz, 1H), 7.78 (t, *J* = 8.0 Hz, 1H), 7.70 (d, *J* = 15.0 Hz, 1H), 7.63 (d, *J* = 4.5 Hz, 1H), 7.40 (dd, *J* = 8.5, 4.5 Hz, 1H), 7.01 (d, *J* = 3.5 Hz, 1H), 4.93 (q, *J* = 7.0 Hz, 2H), 1.47 (t, *J* = 7.0 Hz, 3H). $^{13}$C-NMR (DMSO-d6, 126 MHz): δ 170.8, 147.7, 147.3, 144.9, 141.6, 140.9, 136.3, 133.1, 132.1, 129.5, 128.2, 128.16, 128.1, 124.4, 119.4, 118.6, 118.4, 116.4, 110.2, 107.0, 44.3, 14.2. HRMS (ESI$^{+}$): m/z calcd for $C_{22}H_{18}N_3S_2^{+}$ $(M\text{-}I)^{+}$: 388.0942. Found: 388.0941.

Synthesis of **HS-352**:

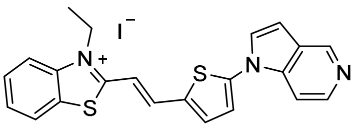

Following the general procedure B, ligand **HS-352** was synthesized in 59% yield (13.3 mg) on 0.0438 mmol scale. $^{1}$H-NMR (DMSO-d6, 500 MHz): δ 9.02 (br s, 1H), 8.52 (d, *J* = 15.5 Hz, 1H), 8.48 (br d, *J* = 5.5 Hz, 1H), 8.43 (d, *J* = 8.0 Hz, 1H), 8.28 (d, *J* = 8.5 Hz, 1H), 8.04 (d, *J* = 4.0 Hz, 1H), 8.00 (d, *J* =3.5 Hz, 1H), 7.91 (d, *J* = 5.5 Hz, 1H), 7.87 (t, *J* = 7.5 Hz, 1H), 7.78 (t, *J* = 7.5 Hz, 1H), 7.73 (d, *J* = 15.5 Hz, 1H), 7.65 (d, *J* = 4.5 Hz, 1H), 7.01 (d, *J* = 3.0 Hz, 1H), 4.94 (q, *J* = 7.0 Hz, 2H), 1.48 (t, *J* = 7.0 Hz, 3H). $^{13}$C-NMR (DMSO-d6, 126 MHz): δ 170.8, 146.5, 143.9, 142.6, 141.5, 140.9, 138.8, 136.0, 133.7, 130.1, 129.5, 128.3, 128.1, 126.0, 124.4, 120.2, 116.5, 110.5, 106.5, 106.5, 105.6, 44.4, 14.2. HRMS (ESI$^{+}$): m/z calcd for $C_{22}H_{18}N_3S_2^{+}$ (M-I)$^{+}$: 388.0942. Found: 388.0941.

Synthesis of **HS-353**:

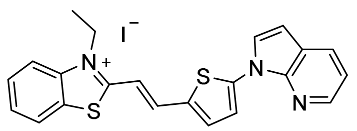

Following the general procedure B, ligand **HS-353** was synthesized in 73% yield (18.2 mg) on 0.0482 mmol scale. $^{1}$H-NMR (DMSO-d6, 500 MHz): δ 8.50 (dd, *J* = 5.0, 1.0 Hz, 1H), 8.49 (d, *J* = 15.5 Hz, 1H), 8.39 (d, *J* = 8.0 Hz, 1H), 8.35 (d, *J* = 4.0 Hz, 1H), 8.23 (d, *J* = 8.5 Hz, 1H), 8.19 (dd, *J* = 8.0, 1.5 Hz, 1H), 8.02 (d, *J* = 4.0 Hz, 1H), 7.85 (dt, *J* = 8.0, 1.0 Hz, 1H), 7.72-7.72 (m, 2H), 7.69 (d, *J* = 15.5 Hz, 1H), 7.38 (dd, *J* = 8.0, 5.0 Hz, 1H), 6.95 (d, *J* = 4.0 Hz, 1H), 4.93 (q, *J* = 7.0 Hz, 2H), 1.48 (t, *J* = 7.0 Hz, 3H). $^{13}$C-NMR (DMSO-d6, 126 MHz): δ 170.9, 146.5, 145.8, 143.7, 142.7, 140.9, 135.9, 132.5, 130.3, 129.3, 128.0, 127.8, 127.3, 124.2, 121.8, 118.4, 116.2, 115.8, 108.5, 105.2, 44.0, 14.1. HRMS (ESI$^{+}$): m/z calcd for $C_{22}H_{18}N_3S_2^{+}$ (M-I)$^{+}$: 388.0942. Found: 388.0941.

Synthesis of **HS-354**:

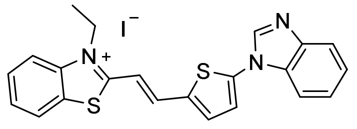

Following the general procedure B, ligand **HS-354** was synthesized in 52% yield (27 mg) on 0.0876 mmol scale. $^{1}$H-NMR (DMSO-d6, 500 MHz): δ 8.57 (s, 1H), 8.48 (d, *J* = 15.5 Hz, 1H), 8.40 (d, *J* = 8.0 Hz, 1H), 8.25 (d, *J* = 8.5 Hz, 1H), 8.20 (d, *J* = 8.5 Hz, 1H), 8.01 (d, *J* = 4.5 Hz, 1H), 7.98 (d, *J* = 8.0 Hz, 1H), 7.85 (dt, *J* = 8.0, 0.5 Hz, 1H), 7.76 (t, *J* = 7.5 Hz, 1H), 7.74 (d, *J* = 4.0 Hz, 1H), 7.69 (dt, *J* = 8.0, 0.5 Hz, 1H), 7.66 (d, *J* = 15.5 Hz, 1H), 7.42 (t, *J* = 7.5 Hz, 1H), 4.92 (q, *J* = 7.0 Hz, 2H), 1.47 (t, *J* = 7.0 Hz, 3H). $^{13}$C-NMR (DMSO-d6, 126 MHz): δ 170.7, 149.8, 142.0, 140.9, 138.8, 137.8, 137.5, 132.1, 129.4, 129.2, 128.1, 128.0, 126.0, 124.3, 123.4, 122.4, 116.3, 114.5, 111.5, 109.6, 44.2, 14.1. HRMS (ESI$^{+}$): m/z calcd for $C_{22}H_{18}N_3S_2^{+}$ (M-I)$^{+}$: 388.0942. Found: 388.0938.

Synthesis of **HS-355**:

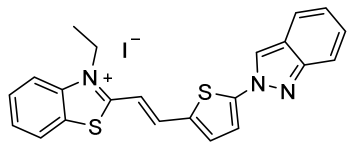

Following the general procedure B, ligand **HS-355** was synthesized in 65% yield (25 mg) on 0.0745 mmol scale. $^{1}$H-NMR (DMSO-d6, 500 MHz): δ 9.23 (s, 1H), 8.49 (d, *J* = 15.5 Hz, 1H), 8.44 (d, *J* = 8.0 Hz, 1H), 8.29 (d, *J* = 8.5 Hz, 1H), 7.98 (d, *J* = 4.0 Hz, 1H), 7.91 (d, *J* = 4.0 Hz, 1H), 7.88 (t, *J* = 7.5 Hz, 1H), 7.83-7.75 (m, 3H), 7.68 (d, *J* = 8.5 Hz, 1H), 7.39 (t, *J* = 7.5 Hz, 1H), 7.17 (t, *J* = 7.5 Hz, 1H), 4.95 (q, *J* = 7.0 Hz, 2H), 1.49 (t, *J* = 7.0 Hz, 3H). $^{13}$C-NMR (DMSO-d6, 126 MHz): δ 170.7, 149.4, 149.0, 141.5, 140.9, 136.1, 135.2, 129.5, 128.3, 128.23, 128.19, 124.4, 123.2, 129.0, 122.7, 121.0, 117.1, 117.0, 116.5, 110.1, 44.4, 14.1. HRMS (ESI$^{+}$): m/z calcd for $C_{22}H_{18}N_3S_2^{+}$ (M-I)$^{+}$: 388.0942. Found: 388.0938.

Synthesis of **HS-356**:

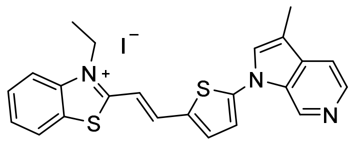

Following the general procedure B, ligand **HS-356** was synthesized in 68% yield (34 mg) on 0.0949 mmol scale. $^{1}$H-NMR (DMSO-d6, 500 MHz): δ 9.28 (br s, 1H), 8.51 (d, *J* = 15.0 Hz, 1H), 8.44-8.38 (m, 2H), 8.26 (d, *J* = 8.5 Hz, 1H), 8.01 (d, *J* = 4.0 Hz, 1H), 7.94 (s, 1H), 7.86 (t, *J* = 7.5 Hz, 1H), 7.77 (t, *J* = 7.5 Hz, 1H), 7.72 (d, *J* = 5.5 Hz, 1H), 7.67 (d, *J* = 15.0 Hz, 1H), 7.62 (d, *J* = 4.0 Hz, 1H), 4.93 (q, *J* = 7.0 Hz, 2H), 2.35 (s, 3H), 1.48 (t, *J* = 7.0 Hz, 3H). $^{13}$C-NMR (DMSO-d6, 126 MHz): δ 170.7, 147.6, 141.7, 140.9, 140.8, 136.6, 135.2, 133.5, 132.4, 132.1, 129.4, 129.1, 128.2, 128.0, 124.4, 118.5, 116.4, 115.1, 114.3, 109.7, 44.2, 14.1, 8.9. HRMS (ESI$^{+}$): m/z calcd for $C_{23}H_{20}N_3S_2^{+}$ (M-I)$^{+}$: 402.1099. Found: 402.1099.

Synthesis of **HS-357**:

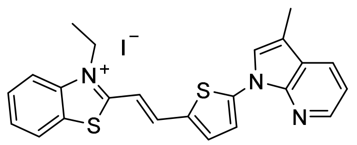

Following the general procedure B, ligand **HS-357** was synthesized in 68% yield (34 mg) on 0.0949 mmol scale. $^{1}$H-NMR (DMSO-d6, 500 MHz): δ 8.51-8.44 (m, 2H), 8.37 (d, *J* = 7.5 Hz, 1H), 8.21 (d, *J* = 8.5 Hz, 1H), 8.16 (dd, *J* = 8.0, 1.5 Hz, 1H), 8.13 (d, *J* = 0.5 Hz, 1H), 7.99 (d, *J* = 4.5 Hz, 1H), 7.82 (dt, *J* = 8.0, 1.0 Hz, 1H), 7.73 (t, *J* = 7.5 Hz, 1H), 7.64 (d, *J* = 4.5 Hz, 1H), 7.62 (d, *J* = 15.0 Hz, 1H), 7.37 (dd, *J* = 7.5, 5.0 Hz, 1H), 4.91 (q, *J* = 7.0 Hz, 2H), 2.36 (s, 3H), 1.47 (t, *J* = 7.0 Hz, 3H). $^{13}$C-NMR (DMSO-d6, 126 MHz): δ 170.8, 146.9, 145.9, 143.8, 142.9, 140.9, 136.4, 132.0, 129.3, 128.6, 127.9, 127.7, 124.2, 124.16, 122.6, 118.0, 116.1, 115.0, 114.6, 107.9, 44.0, 14.1, 9.5. HRMS (ESI$^{+}$): m/z calcd for $C_{23}H_{20}N_3S_2^{+}$ (M-I)$^{+}$: 402.1099. Found: 402.1095.

Synthesis of **HS-358**:

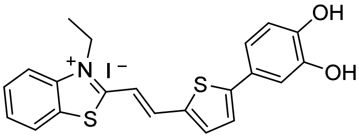


Following the general procedure B, ligand **HS-358** was synthesized in 63% yield (42 mg) on 0.132 mmol scale. $^{1}$H-NMR (DMSO-d6, 500 MHz): δ 9.48 (br s, 2H), 8.43 (d, *J* = 15.5 Hz, 1H), 8.40 (d, *J* = 8.0 Hz, 1H), 8.24 (d, *J* = 8.5 Hz, 1H), 7.94 (d, *J* = 4.0 Hz, 1H), 7.85 (t, *J* = 8.5 Hz, 1H), 7.76 (t, *J* = 8.5 Hz, 1H), 7.59 (d, *J* = 15.5 Hz, 1H), 7.53 (d, *J* = 4.0 Hz, 1H), 7.16 (d, *J* = 2.0 Hz, 1H), 7.14 (dd, *J* = 8.0, 2.0 Hz, 1H), 6.85 (d, *J* = 8.0 Hz, 1H), 4.90 (q, *J* = 7.0 Hz, 2H), 1.46 (t, *J* = 7.0 Hz, 3H). $^{13}$C-NMR (DMSO-d6, 126 MHz): δ 170.7, 153.1, 147.6, 146.0, 141.7, 140.9, 137.9, 136.7, 129.4, 128.1, 127.9, 124.3, 124.2, 124.1, 118.0, 116.3, 113.2, 109.6, 44.1, 14.1. HRMS (ESI$^{+}$): m/z calcd for $C_{21}H_{18}NO_2S_2^{+}$ (M-I)$^{+}$: 380.0779. Found: 380.0775.

Synthesis of **HS-359**:

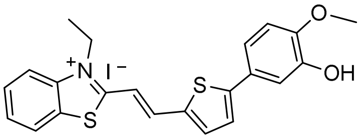


Following the general procedure B, ligand **HS-359** was synthesized in 82% yield (53 mg) on 0.124 mmol scale. $^{1}$H-NMR (DMSO-d6, 500 MHz): δ 9.40 (s, 1H), 8.44 (d, *J* = 15.0 Hz, 1H), 8.40 (d, *J* = 8.0 Hz, 1H), 8.25 (d, *J* = 8.5 Hz, 1H), 7.94 (d, *J* = 4.0 Hz, 1H), 7.85 (t, *J* = 7.5 Hz, 1H), 7.76 (t, *J* = 8.0 Hz, 1H), 7.62 (d, *J* = 15.5 Hz, 1H), 7.60 (d, *J* = 4.0 Hz, 1H), 7.25 (dd, *J* = 8.5, 2.0 Hz, 1H), 7.20 (d, *J* = 2.0 Hz, 1H), 7.04 (d, *J* = 8.5 Hz, 1H), 4.90 (q, *J* = 7.0 Hz, 2H), 3.84 (s, 3H), 1.46 (t, *J* = 7.0 Hz, 3H). $^{13}$C-NMR (DMSO-d6, 126 MHz): δ 170.7, 152.3, 149.3, 147.1, 141.6, 140.9, 137.7, 137.2, 129.4, 128.2, 128.0, 125.5, 124.8, 124.3, 117.6, 116.4, 112.9, 112.7, 110.0, 55.8, 44.2, 14.1. HRMS (ESI$^{+}$): m/z calcd for $C_{22}H_{20}NO_2S_2^{+}$ (M-I)$^{+}$: 394.0935. Found: 394.0934.

Synthesis of **HS-360**:

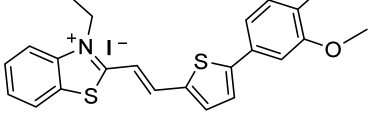


Following the general procedure B, ligand **HS-360** was synthesized in 15% yield (10 mg) on 0.124 mmol scale. $^{1}$H-NMR (DMSO-d6, 500 MHz): δ 9.68 (br s, 1H), 8.44 (d, *J* = 15.5 Hz, 1H), 8.39 (d, *J* = 8.0 Hz, 1H), 8.24 (d, *J* = 8.5 Hz, 1H), 7.95 (d, *J* = 4.0 Hz, 1H), 7.85 (t, *J* = 7.5 Hz, 1H), 7.76 (t, *J* = 8.0 Hz, 1H), 7.67 (d, *J* = 4.0 Hz, 1H), 7.61 (d, *J* = 15.0 Hz, 1H), 7.31 (d, *J* = 1.5 Hz, 1H), 7.26 (dd, *J* = 8.5, 2.0 Hz, 1H), 6.89 (d, *J* = 8.0 Hz, 1H), 4.90 (q, *J* = 7.0 Hz, 2H), 3.87 (s, 3H), 1.46 (t, *J* = 7.0 Hz, 3H). $^{13}$C-NMR (DMSO-d6, 126 MHz): δ 170.7, 153.0, 148.6, 148.2, 141.7, 140.9, 137.9, 136.9, 129.4, 128.1, 128.0, 124.7, 124.3, 124.1, 119.5, 116.3, 116.2, 109.9, 109.7, 55.8, 44.1, 14.1. HRMS (ESI$^{+}$): m/z calcd for $C_{22}H_{20}NO_2S_2^{+}$ (M-I)$^{+}$: 394.0935. Found: 394.0933.

$^{1}H$ and $^{13}C$-NMR of **3a**:

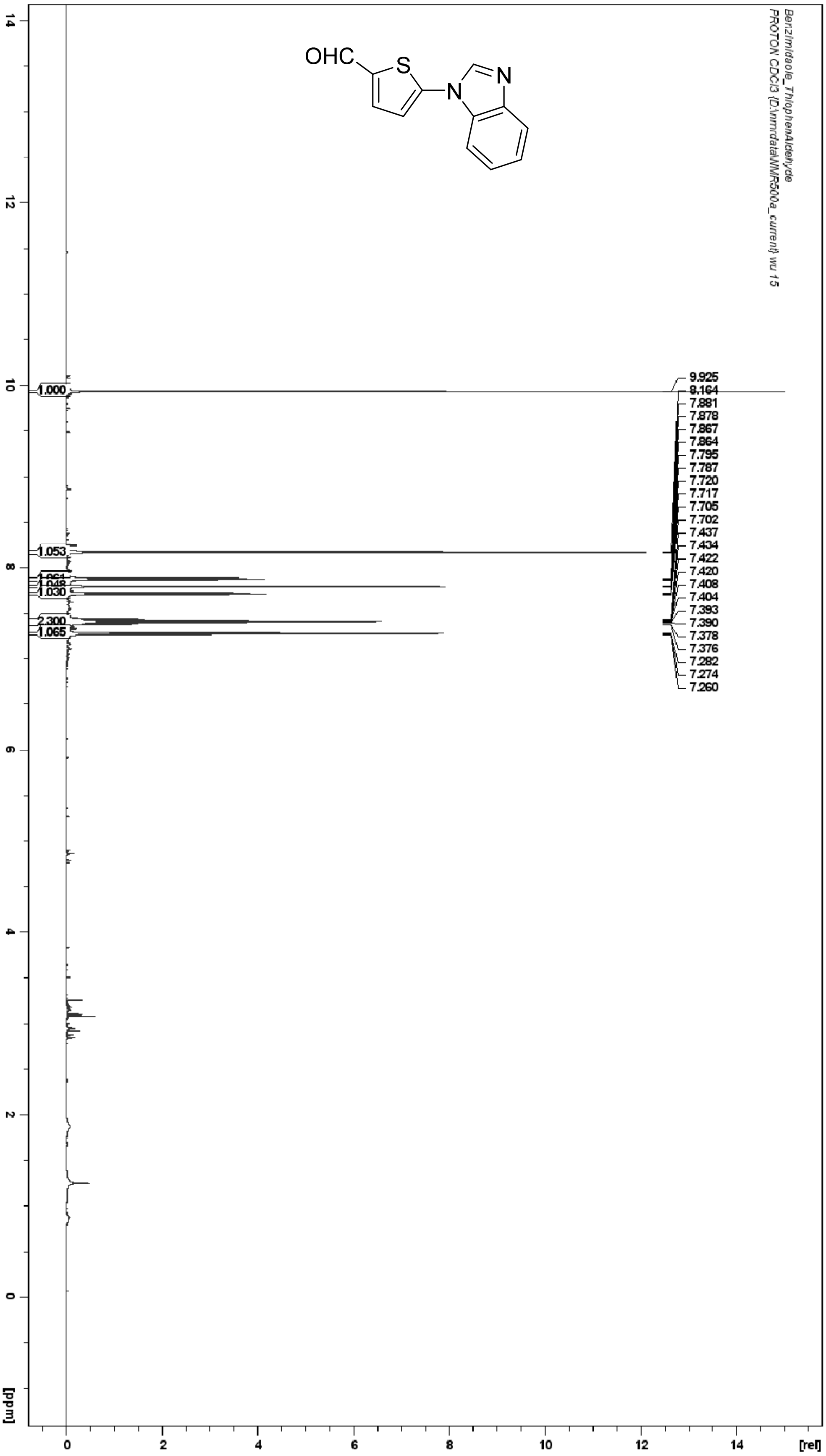

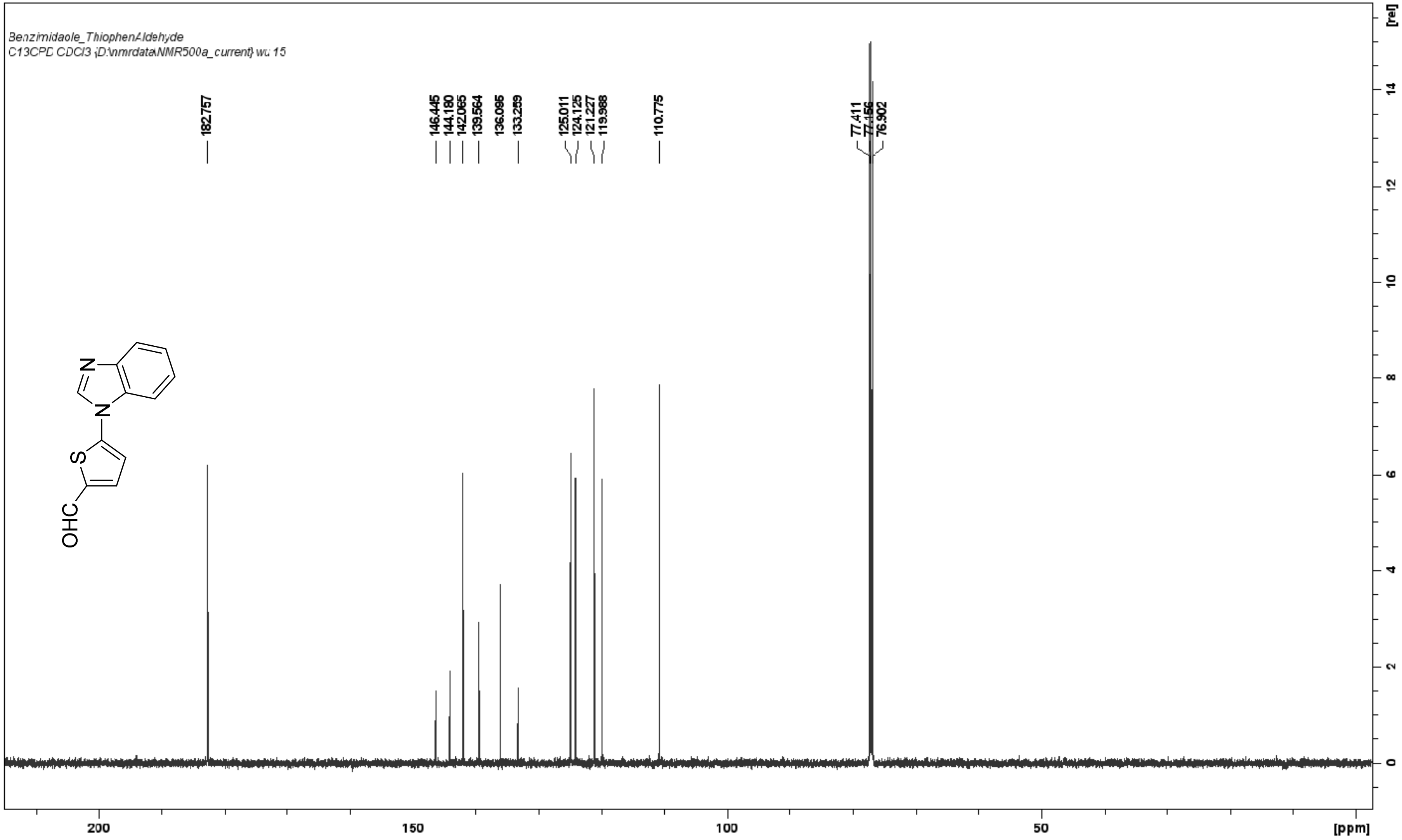
Benzimidaole_ThiophenAldehyde
C13CPD CDCl3 {D:\nmrdata\NMR500a_current} wu 15
182.757
146.445
144.180
142.065
139.564
136.095
133.259
125.011
124.125
121.227
119.988
110.775
77.411
77.156
76.902
OHC
S
N
N
200
150
100
50
[ppm]
[rel]
0
2
4
6
8
10
12
14

$^1H$ and $^{13}C$-NMR of **3b**:

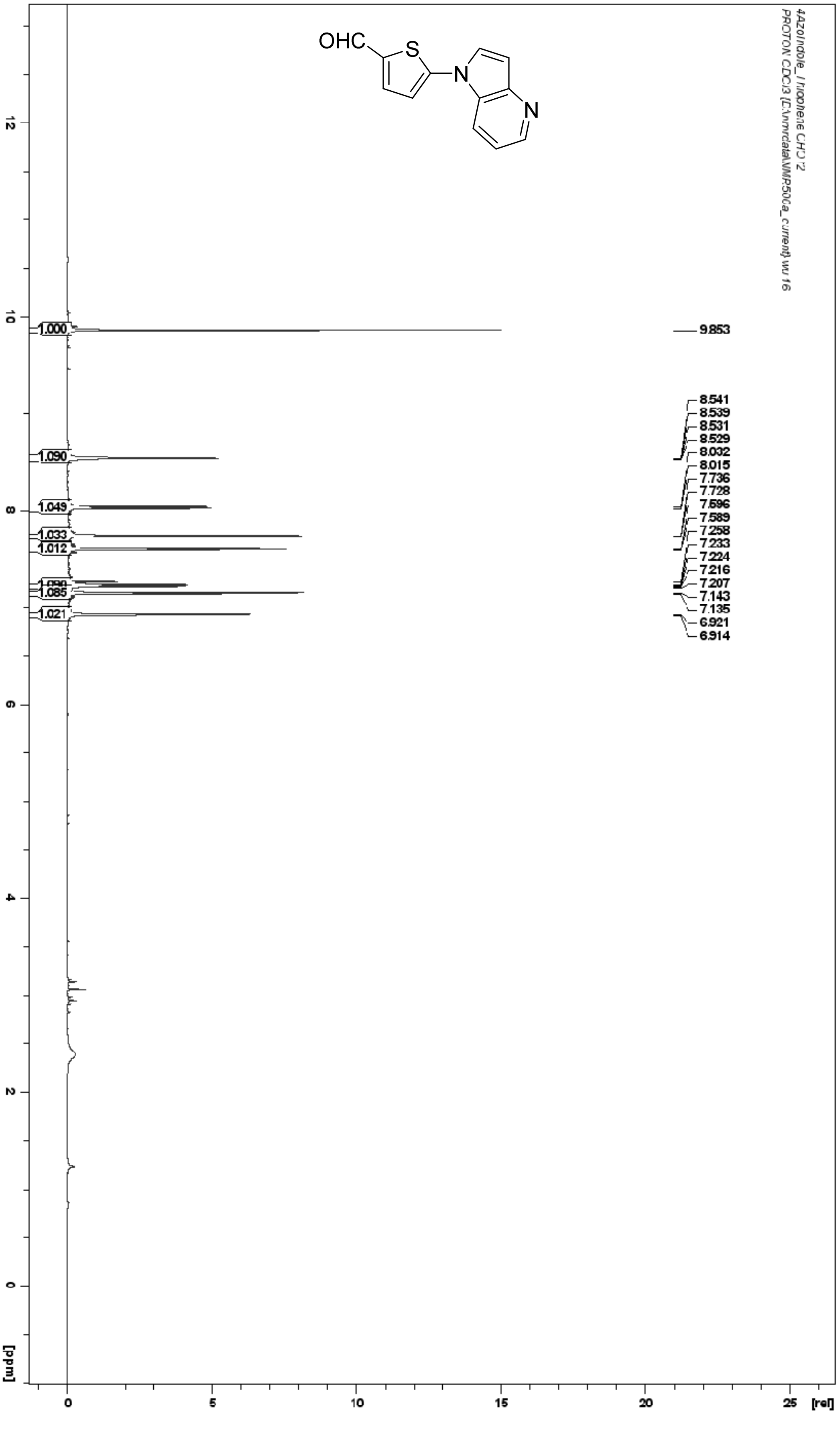

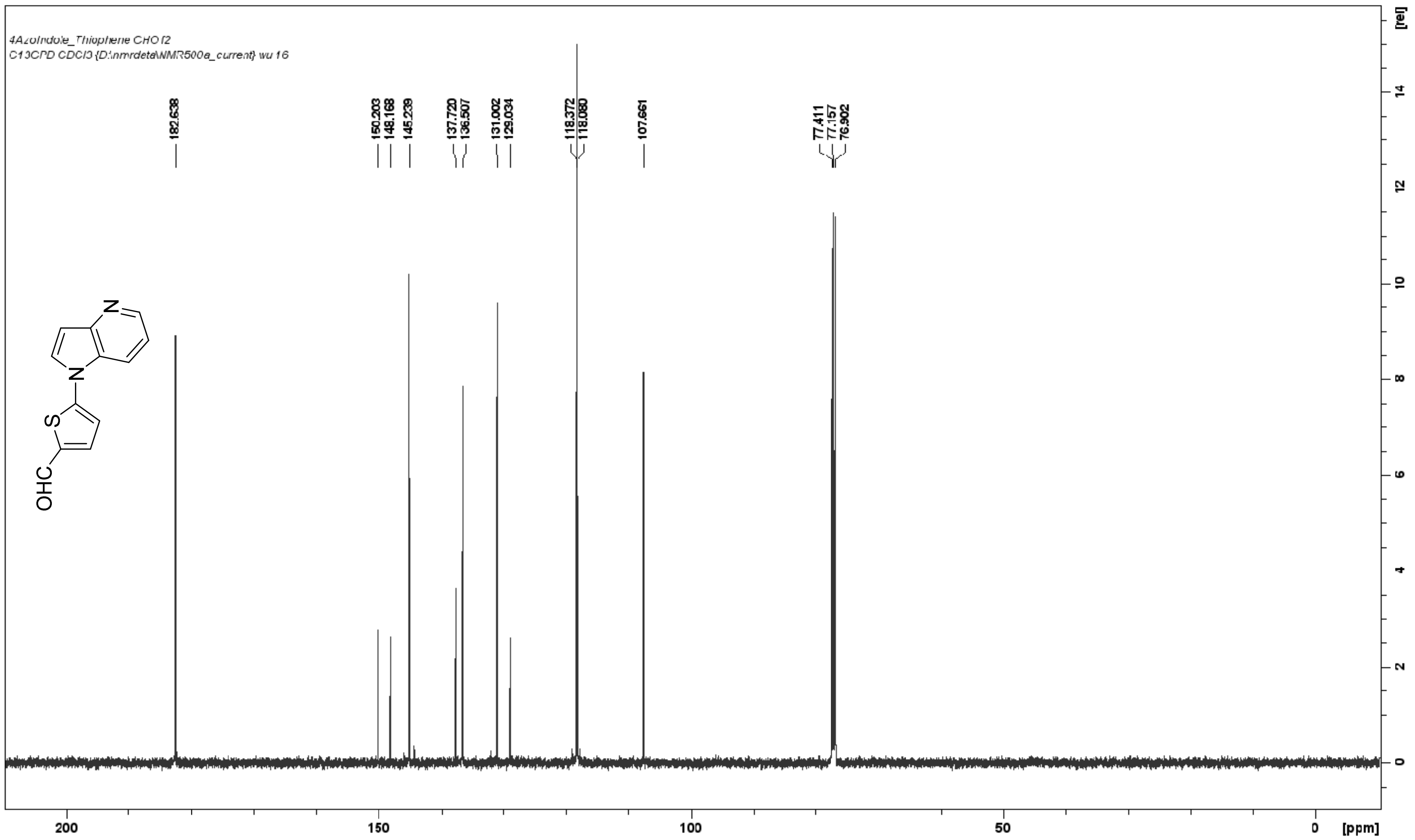
4AzaIndole_Thiophene CHO f2
C13CPD CDCl3 {D:\nmrdata\NMR500a_current} wu 16
182.638
150.203
148.168
145.239
137.720
136.507
131.002
129.034
118.372
118.080
107.661
77.411
77.157
76.902
OHC
S
N
N
200
150
100
50
0
[ppm]
[rel]

$^{1}H$ and $^{13}C$-NMR of **3c**:

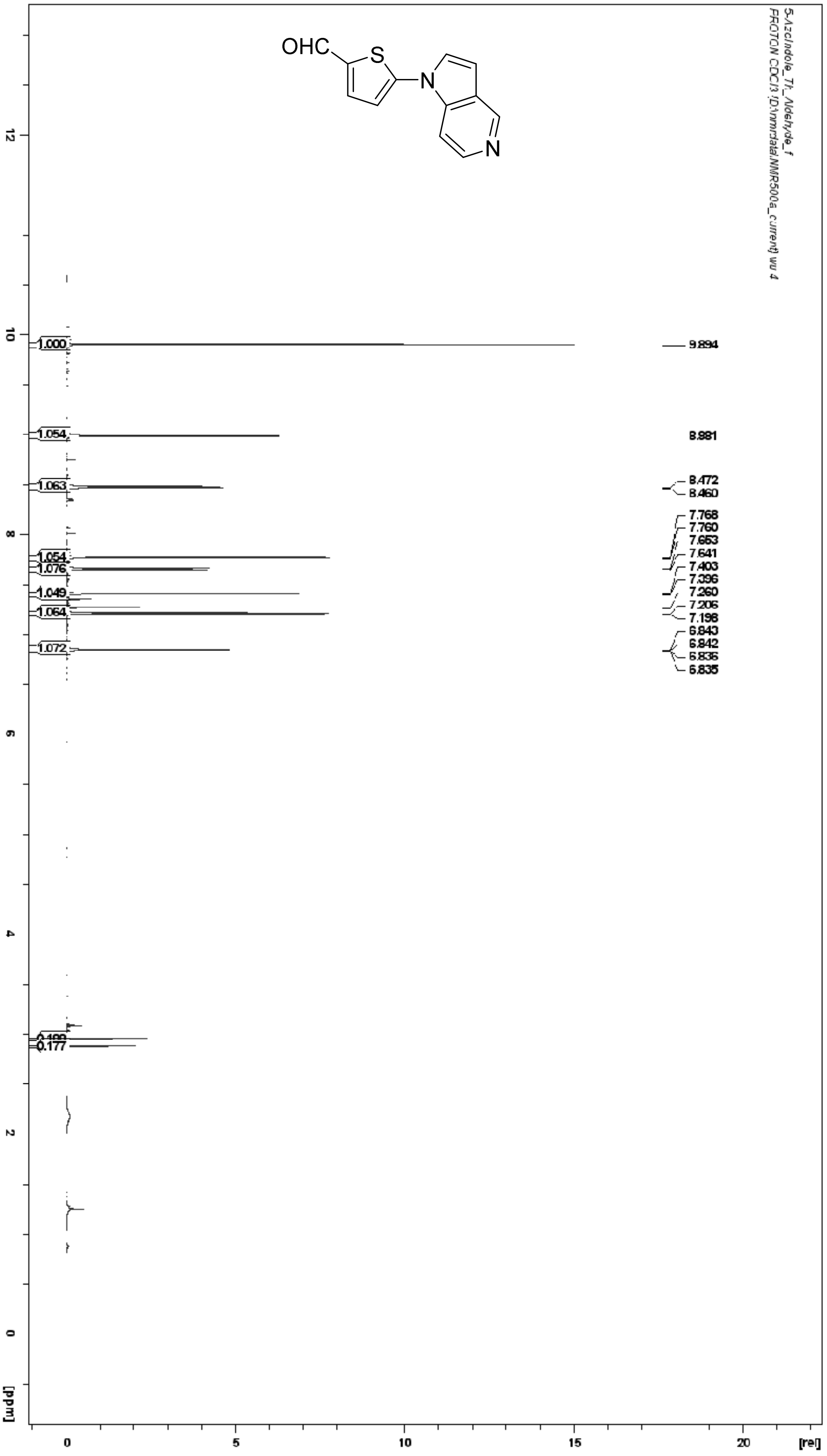

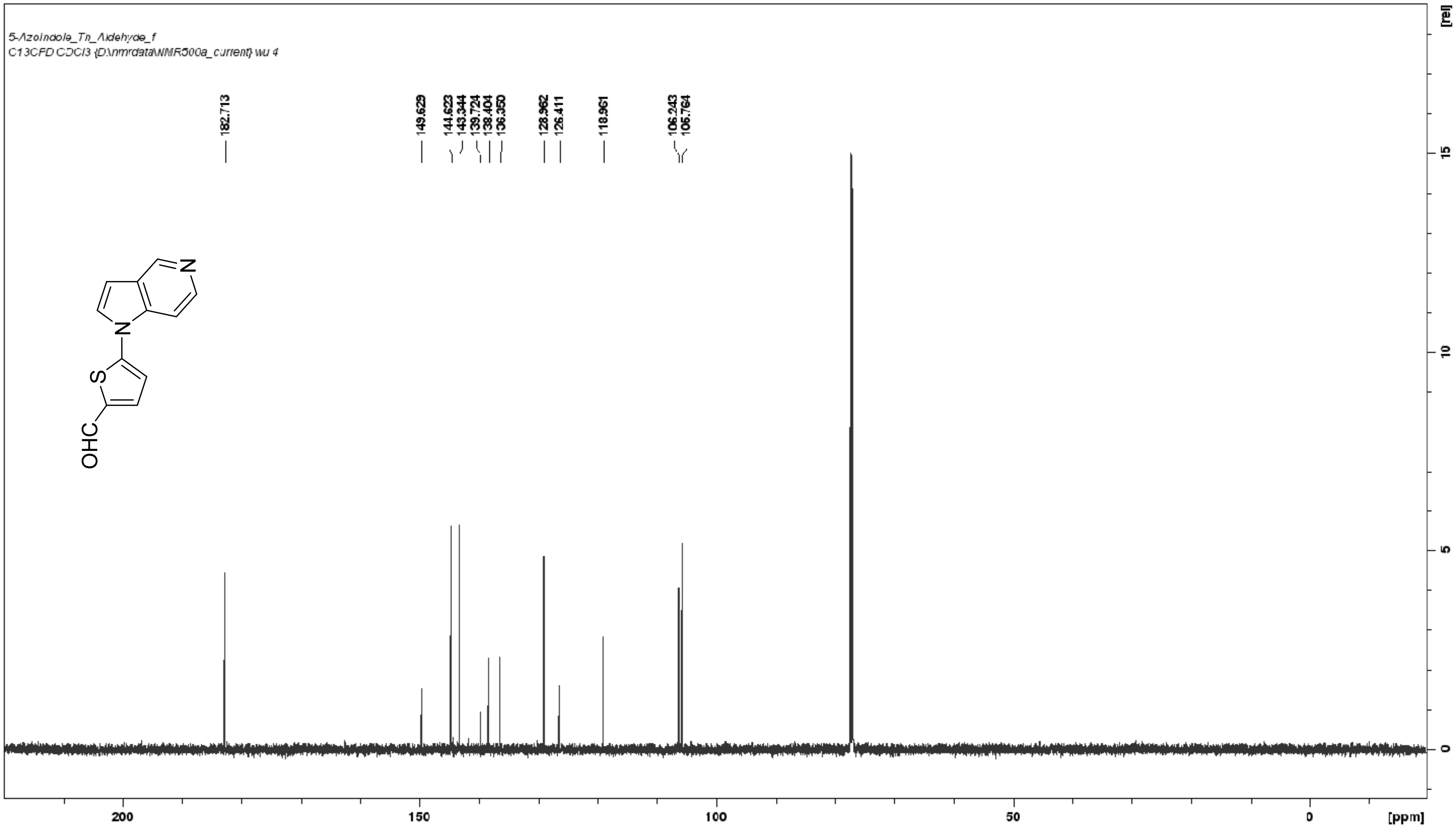
5-Azaindole_Th_Aldehyde_f
C13CPD CDCl3 {D:\nmrdata\NMR500a_current} wu 4
OHC
S
N
N
182.713
149.629
144.623
143.344
139.724
138.404
136.350
128.962
126.411
118.961
106.243
105.764
200
150
100
50
0
[ppm]
0
5
10
15
[rel]

$^{1}$H and $^{13}$C-NMR of **3d**:

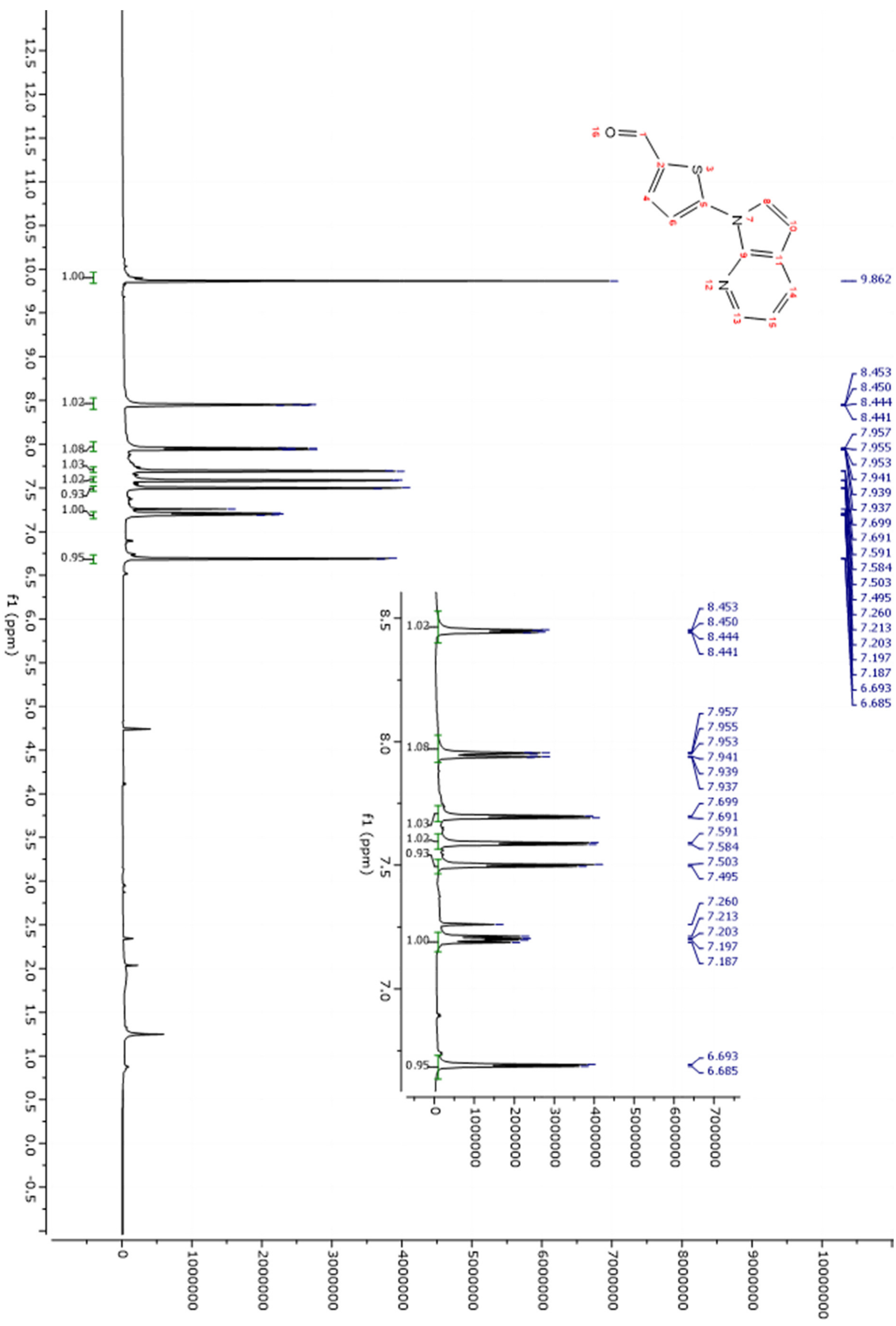

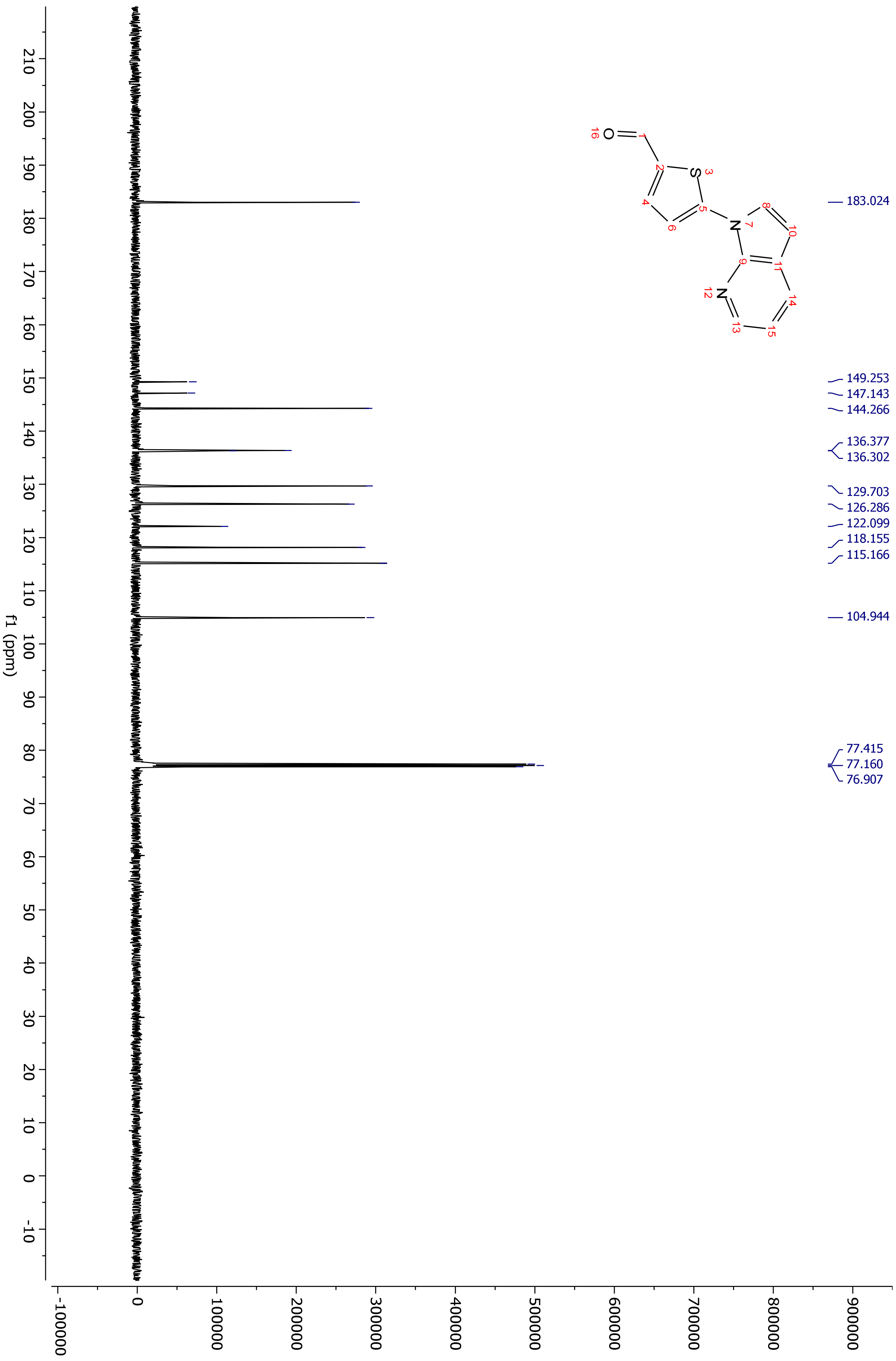

183.024
149.253
147.143
144.266
136.377
136.302
129.703
126.286
122.099
118.155
115.166
104.944
77.415
77.160
76.907
f1 (ppm)

$^{1}$H and $^{13}$C-NMR of **3e**:

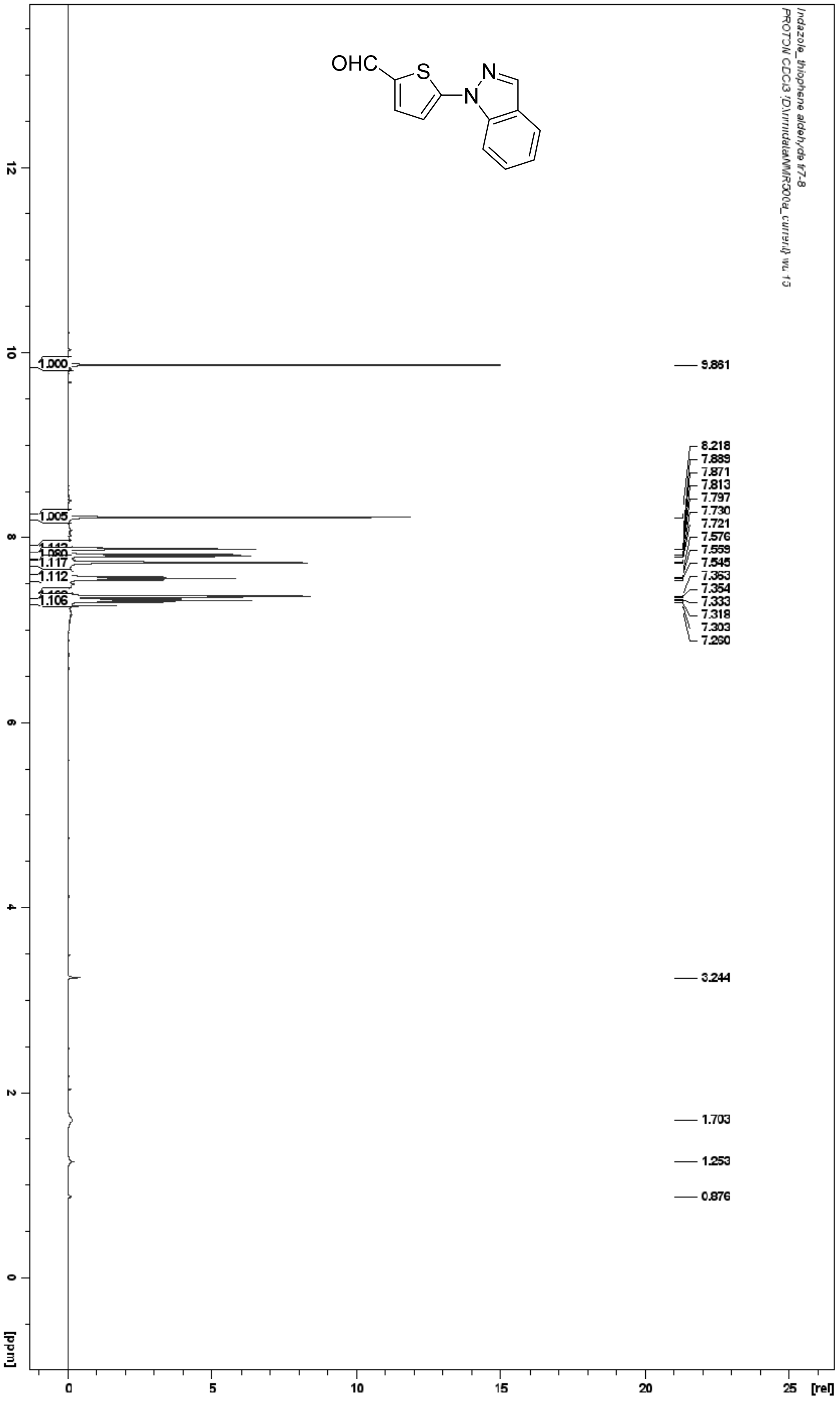

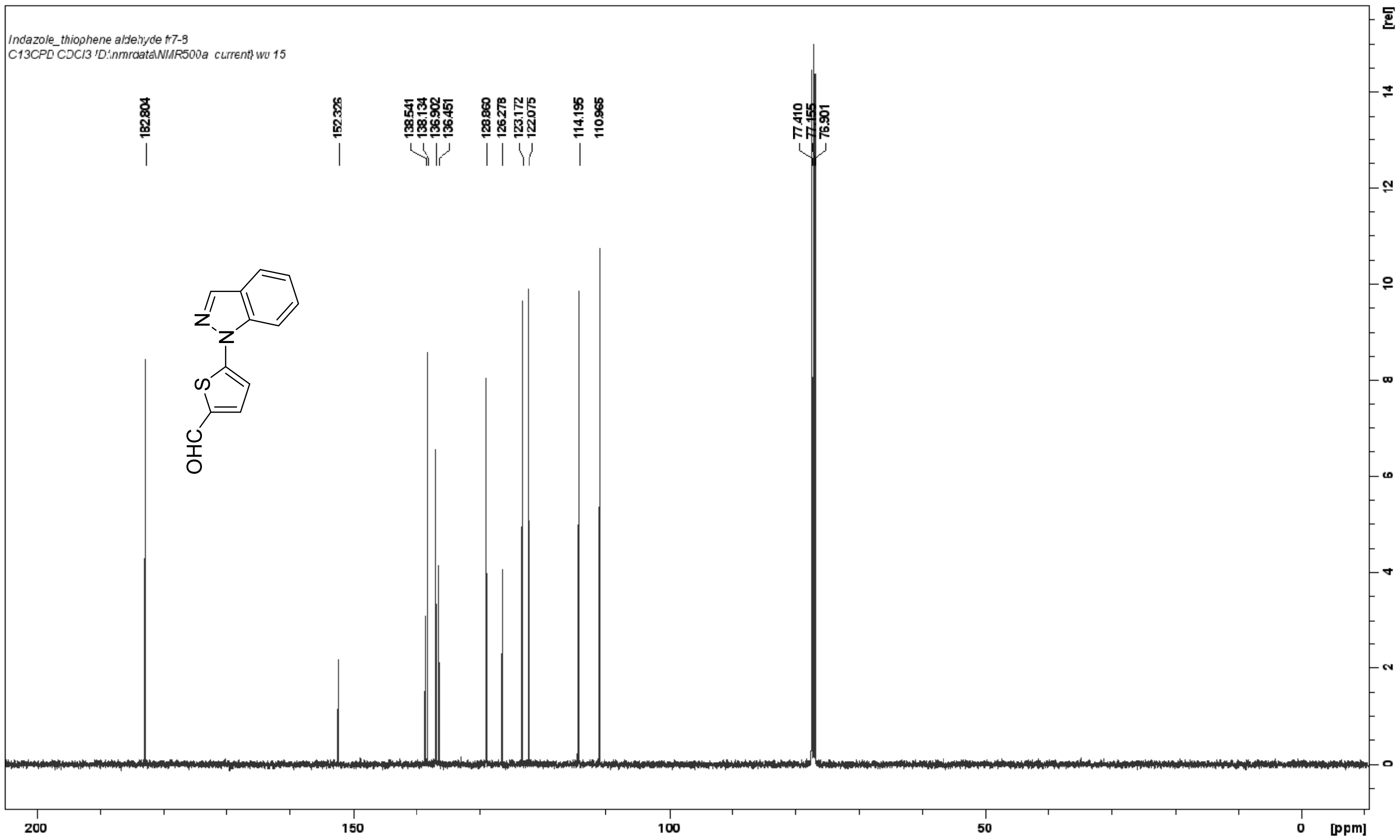

Indazole_thiophene aldehyde fr7-8
C13CPD CDCl3 {D:\nmrdata\NMR500a current} wu 15
182.804
152.326
138.541
138.134
136.902
136.451
128.860
126.278
123.172
122.075
114.195
110.965
77.410
77.155
76.901
OHC
S
N
N
200
150
100
50
0
[ppm]
0
2
4
6
8
10
12
14
[rel]

$^{1}$H and $^{13}$C-NMR of **3f**:

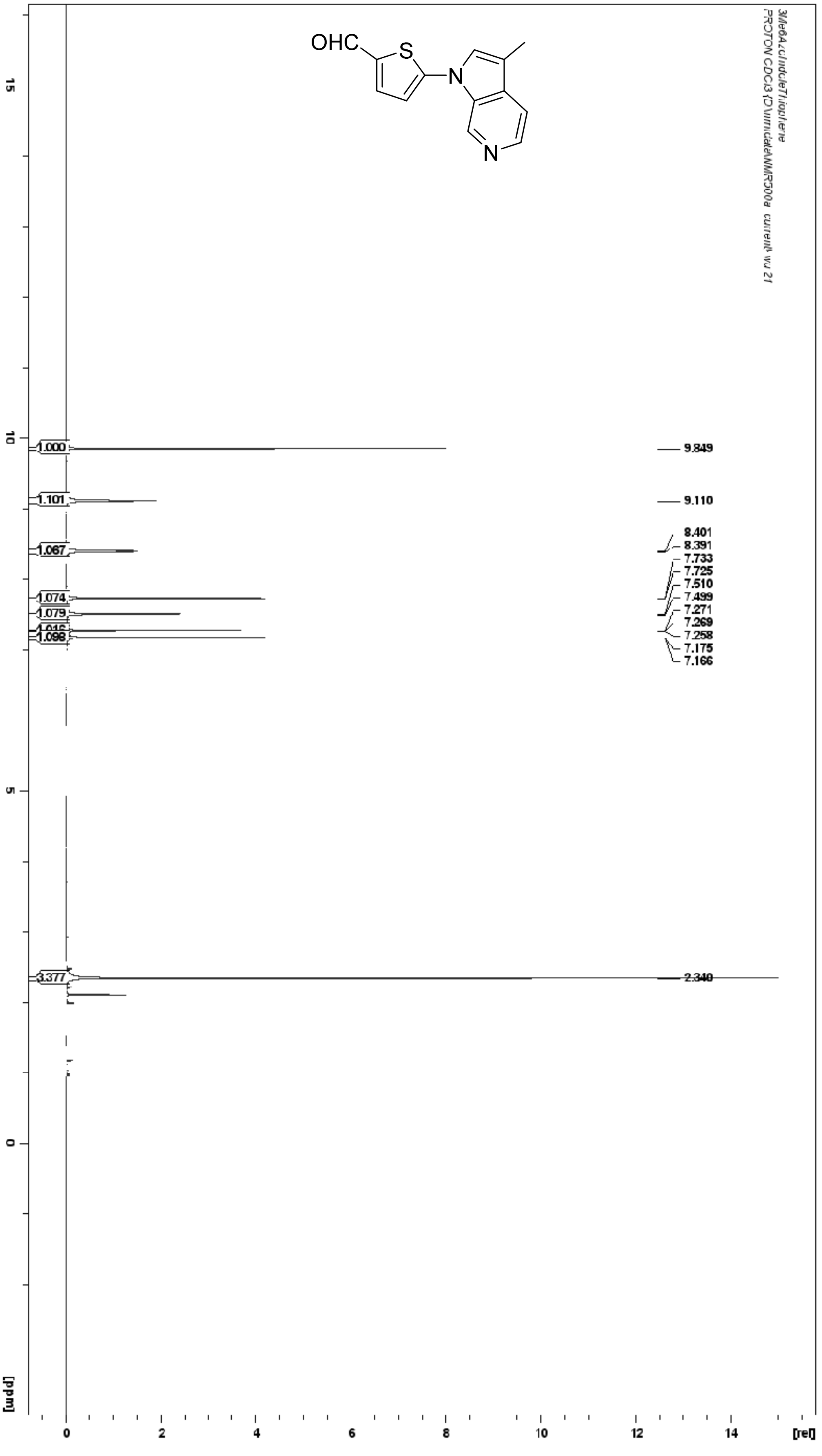

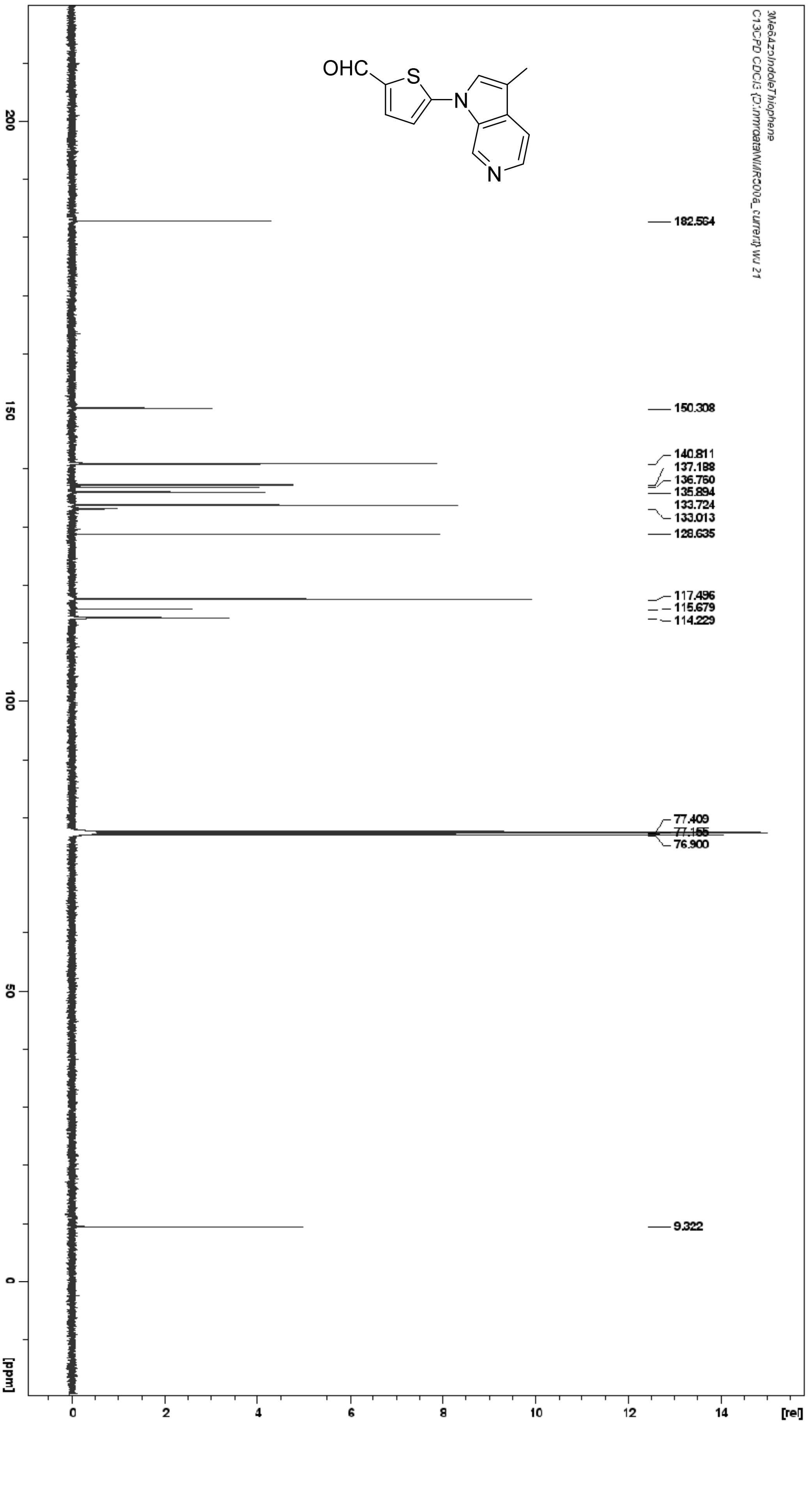

3Me6AzaIndoleThiophene
C13CPD CDCl3 {C:\nmrdata\NMR500a_current} wu 21
OHC
S
N
N
182.564
150.308
140.811
137.188
136.760
135.894
133.724
133.013
128.635
117.496
115.679
114.229
77.409
77.155
76.900
9.322
200
150
100
50
0
[ppm]
0
2
4
6
8
10
12
14
[rel]

$^{1}$H and $^{13}$C-NMR of **3g**:

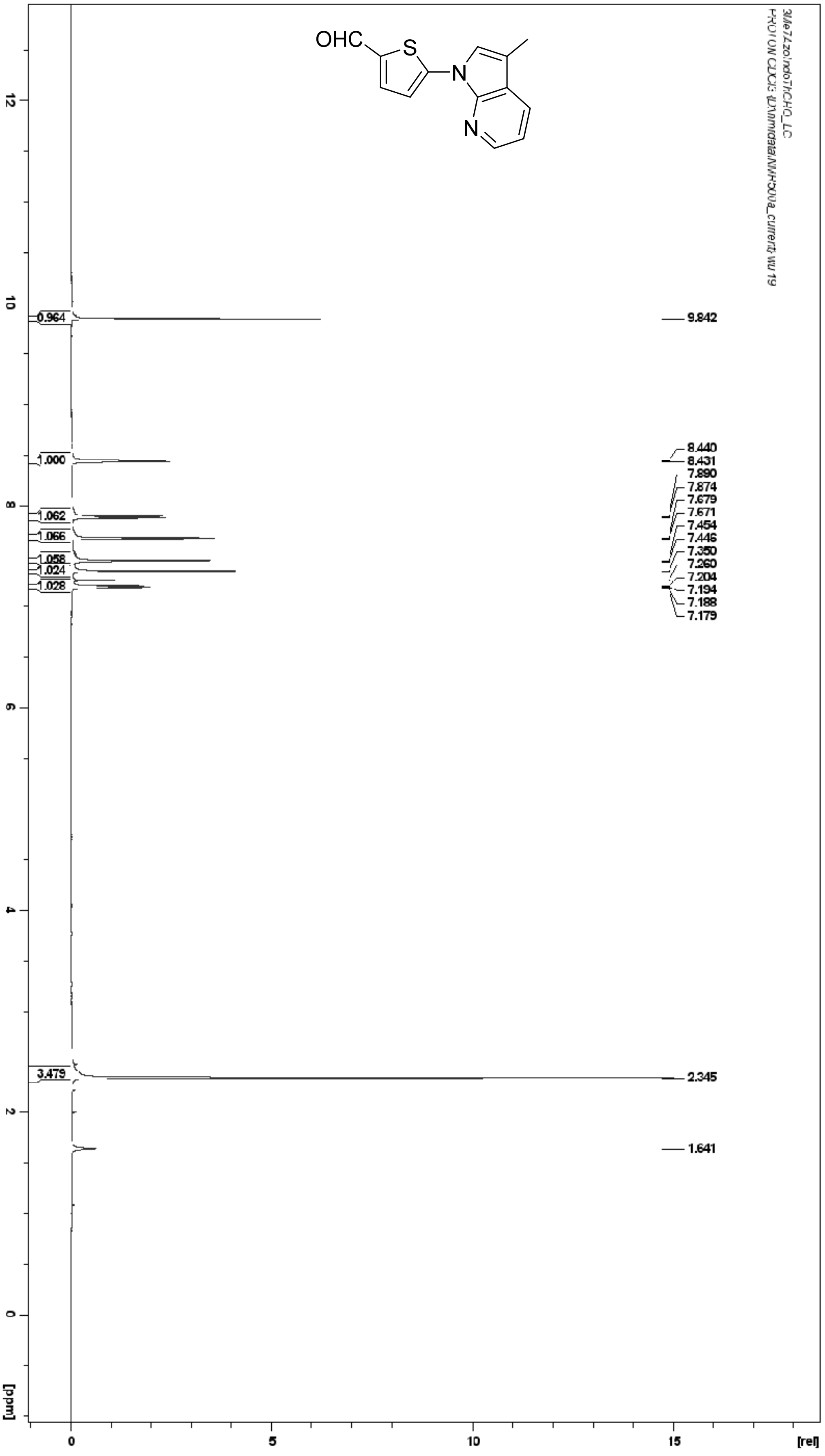

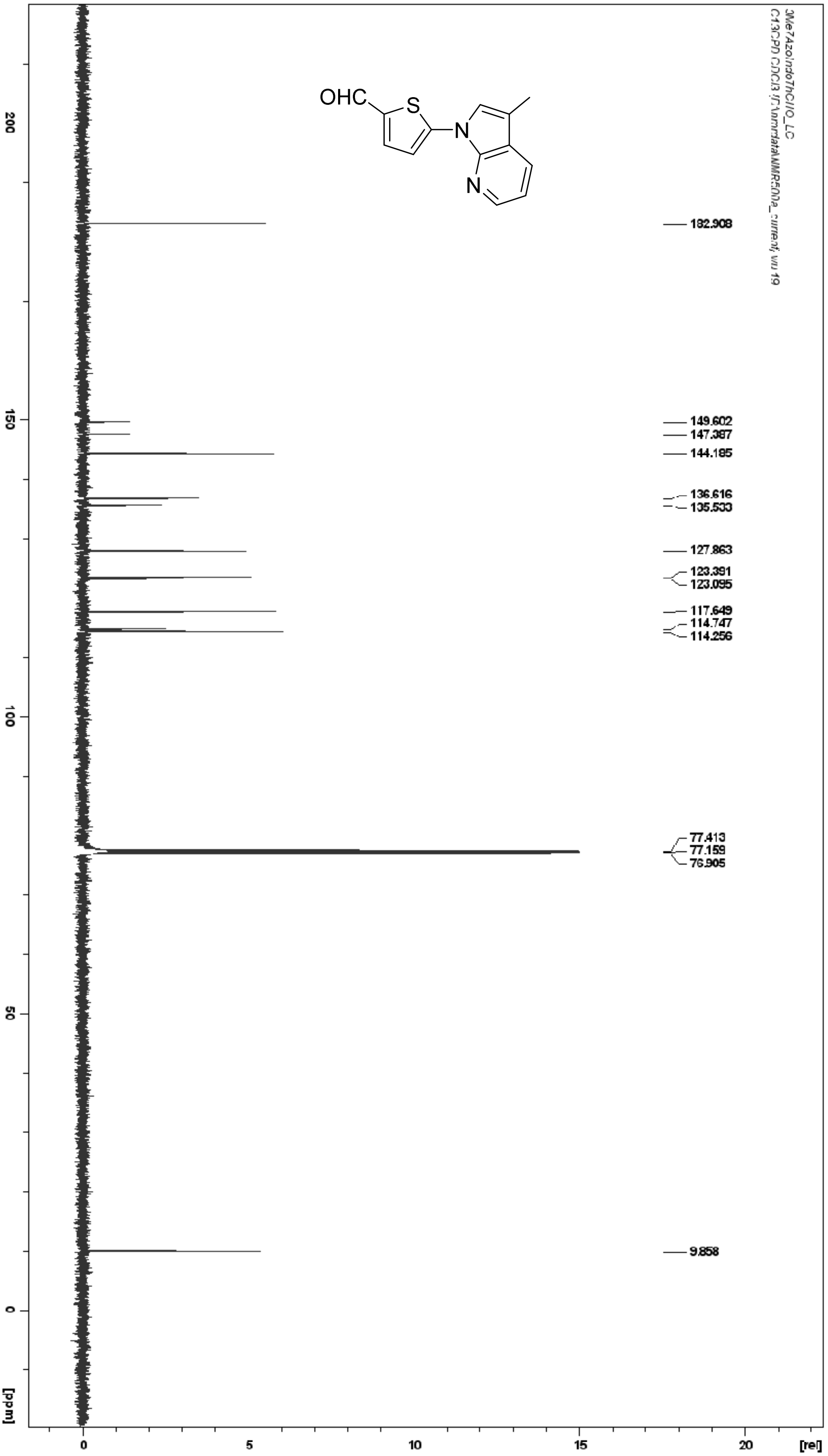

3Me7AzoIndoThCHO_LC
C13CPD CDCl3
OHC
S
N
N
182.908
149.602
147.387
144.185
136.616
135.533
127.863
123.391
123.095
117.649
114.747
114.256
77.413
77.159
76.905
9.858
200
150
100
50
0
[ppm]
0
5
10
15
20
[rel]

$^{1}H$ and $^{13}C$-NMR of **3h**:

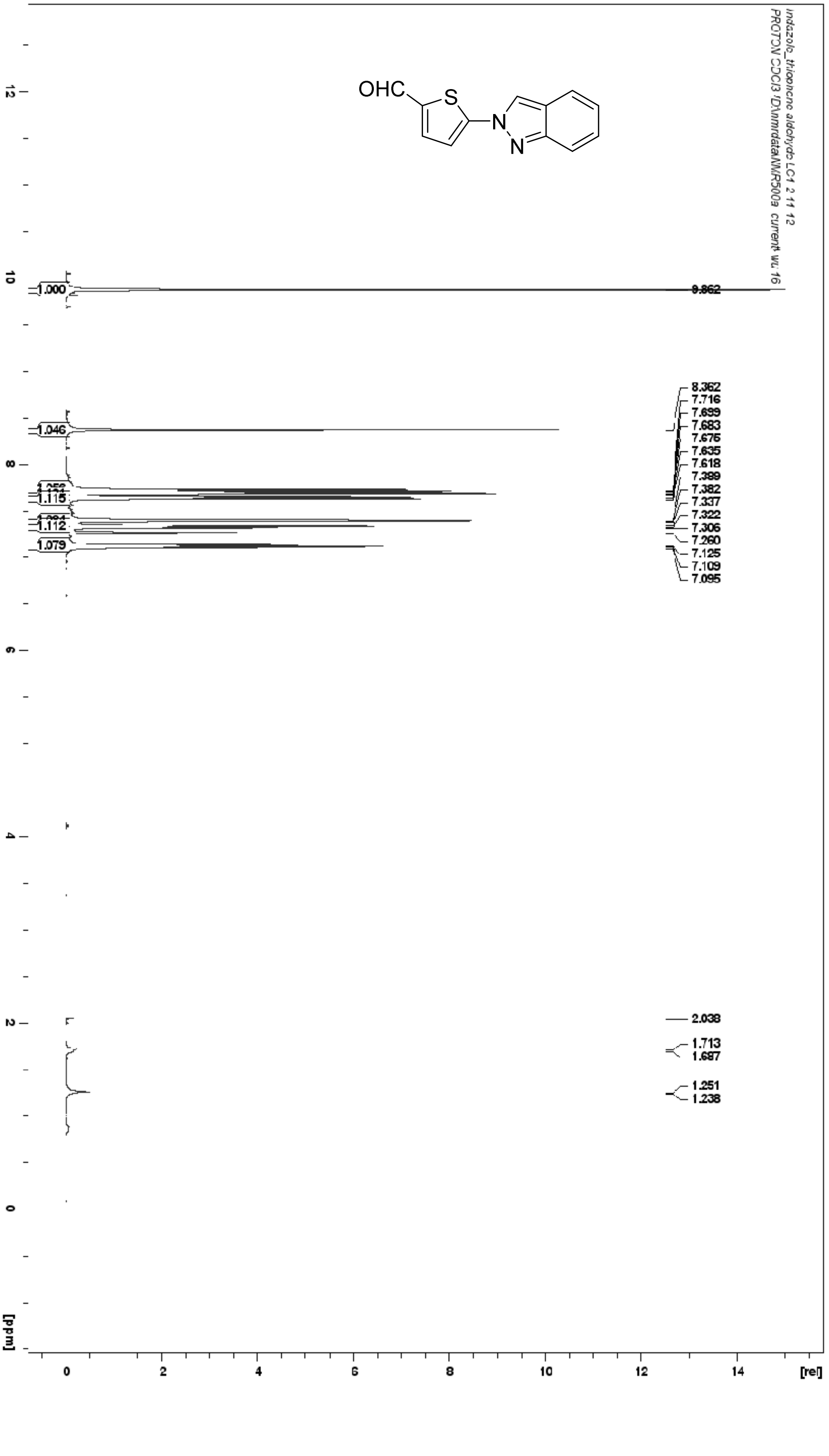

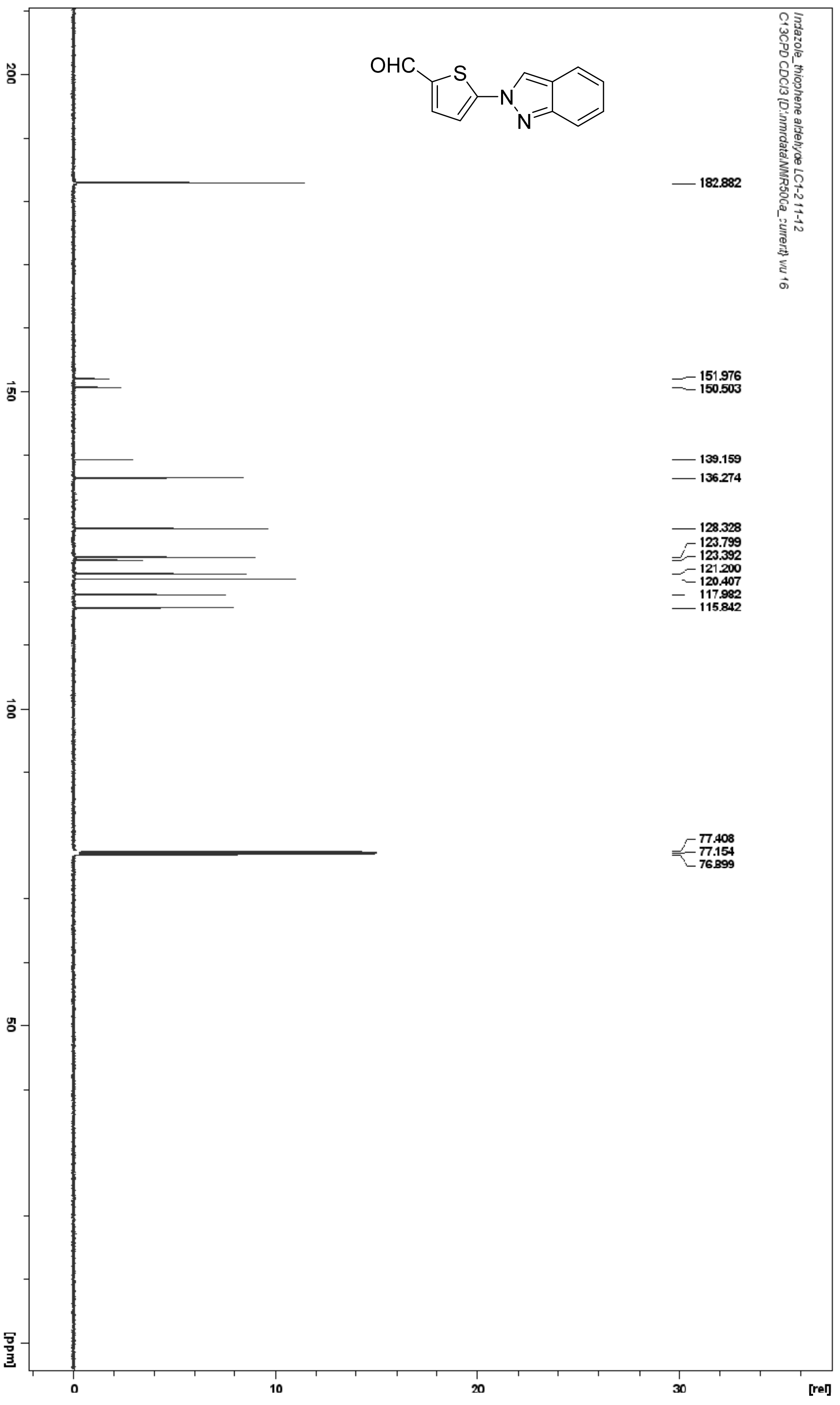

Indazole_thiophene aldehyde LC1-2 11-12
C13CPD CDCl3 [D:\nmrdata\NMR500a_current] vu 16
OHC
S
N
N
182.882
151.976
150.503
139.159
136.274
128.328
123.799
123.392
121.200
120.407
117.982
115.842
77.408
77.154
76.899
200
150
100
50
[ppm]
0
10
20
30
[rel]

$^{1}$H and $^{13}$C-NMR of **7b**:

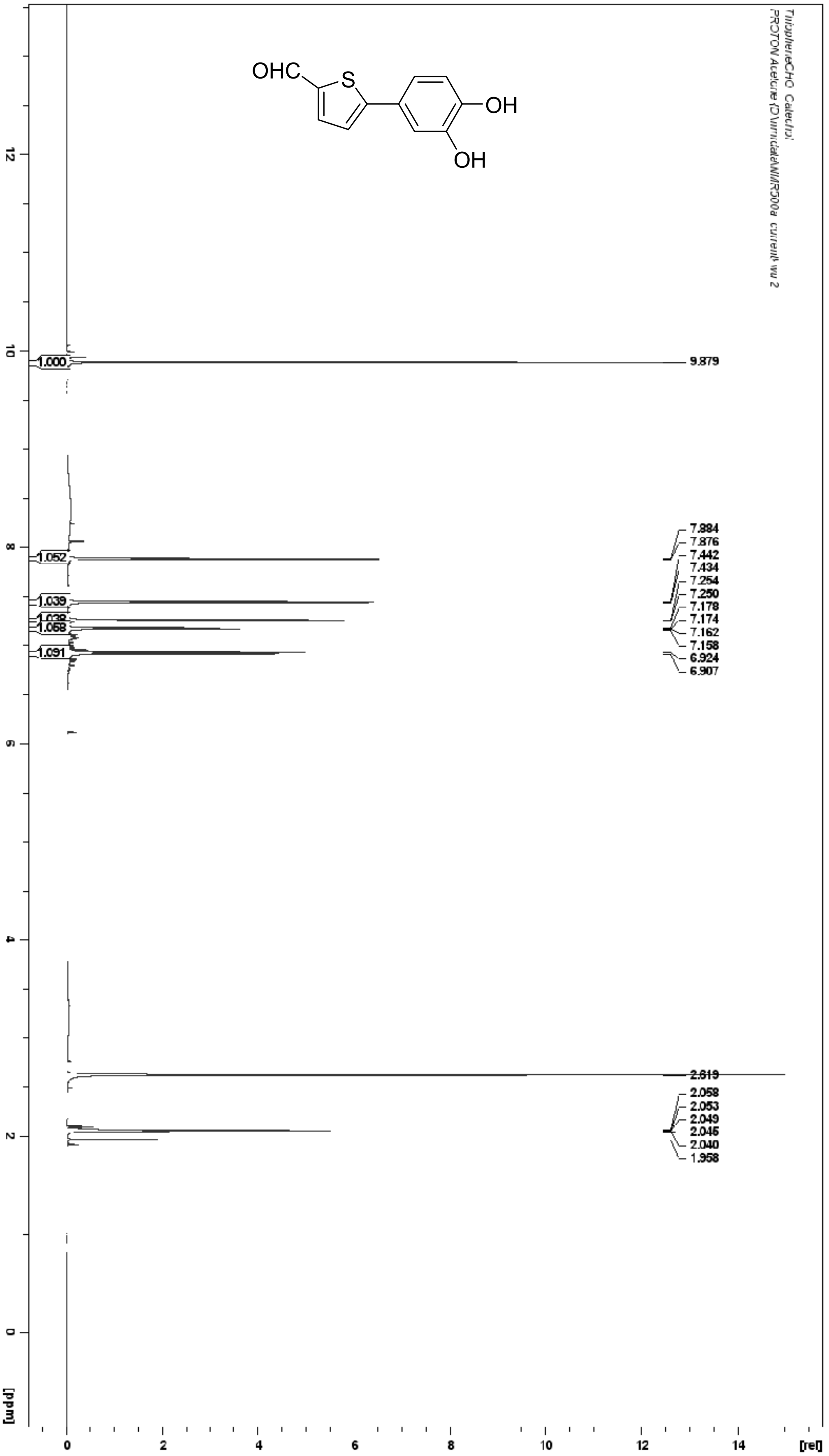

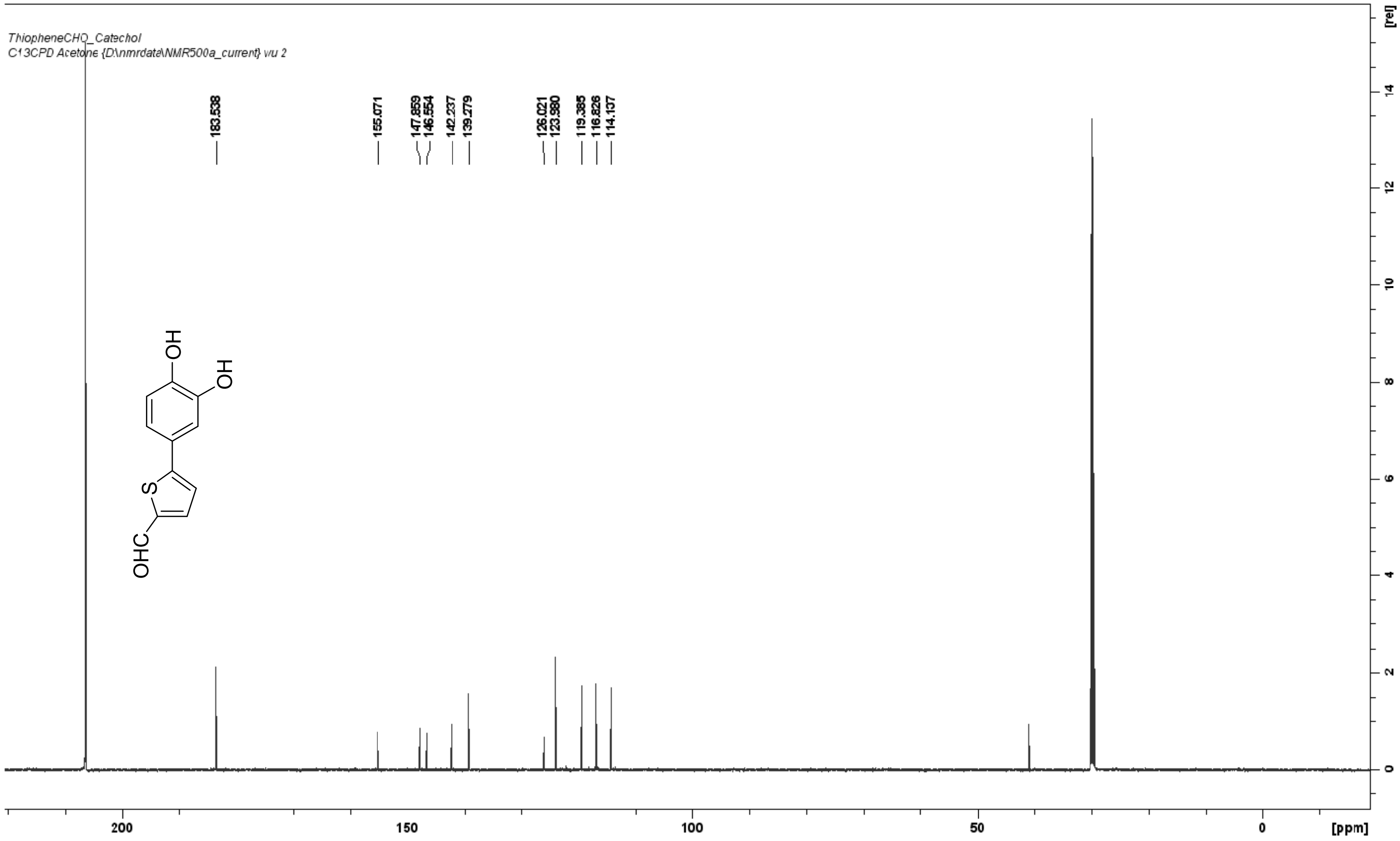
ThiopheneCHO_Catechol
C13CPD Acetone {D:\nmrdata\NMR500a_current} vu 2
183.538
155.071
147.859
146.554
142.237
139.279
126.021
123.980
119.385
116.826
114.137
OHC
S
OH
OH
200
150
100
50
0
[ppm]
[rel]
0
2
4
6
8
10
12
14

$^{1}H$ and $^{13}C$-NMR of **7c**:

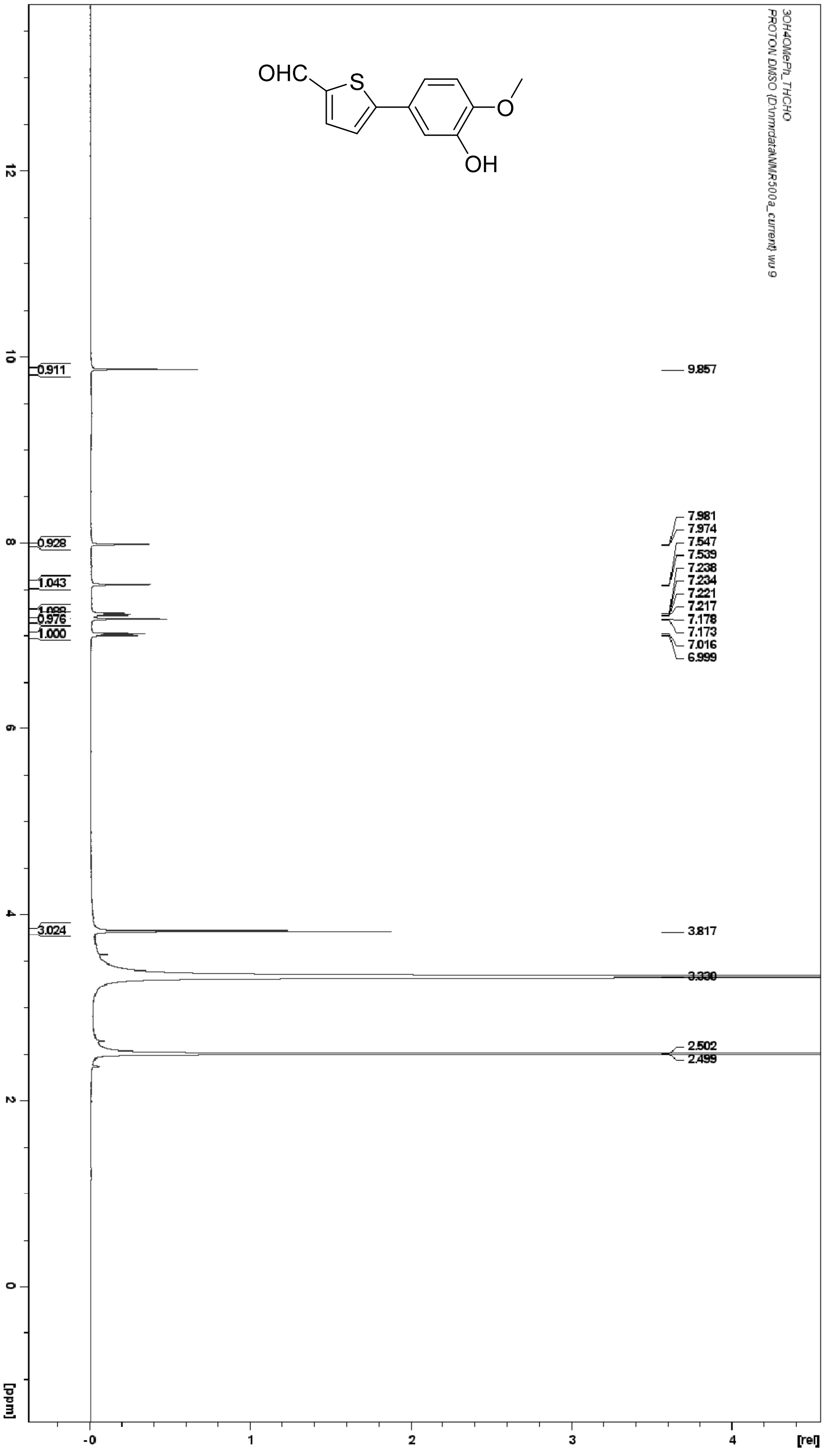

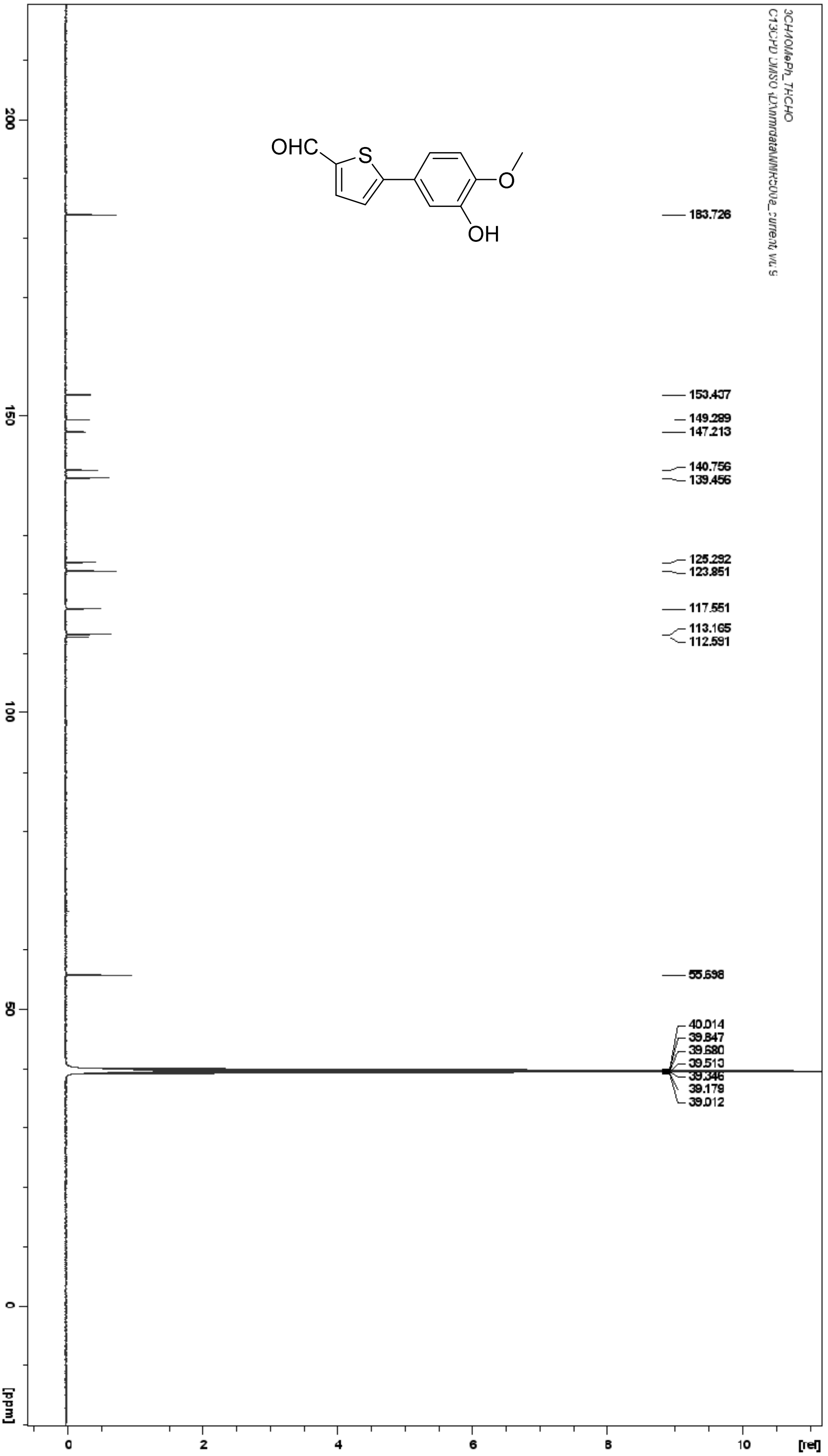

3CH/OMePh_THCHO
OHC
S
O
OH
183.726
153.437
149.289
147.213
140.756
139.456
125.292
123.851
117.551
113.165
112.591
55.598
40.014
39.847
39.680
39.513
39.346
39.179
39.012
200
150
100
50
0
[ppm]
0
2
4
6
8
10
[rel]

$^{1}$H and $^{13}$C-NMR of **7d**:

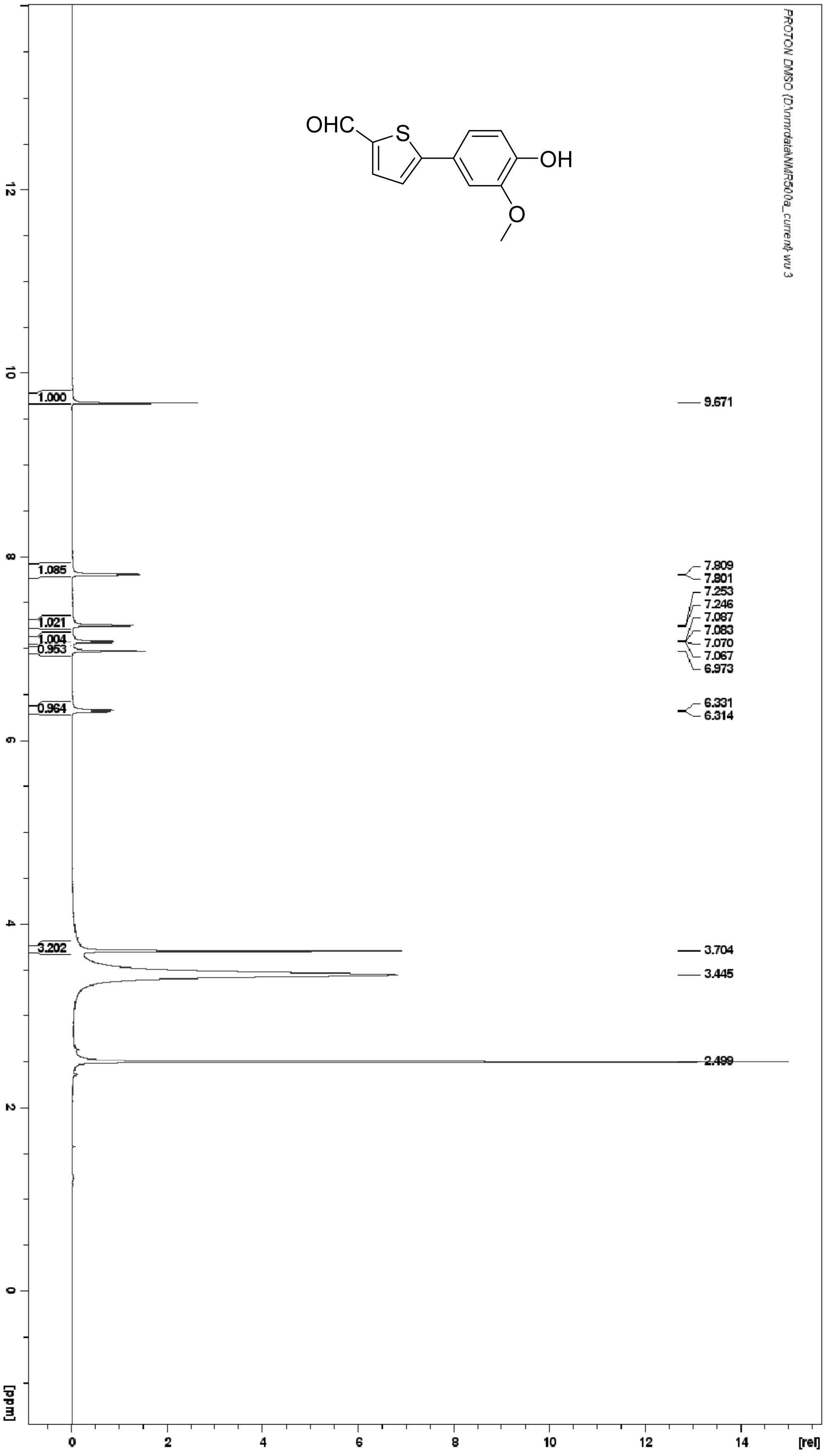

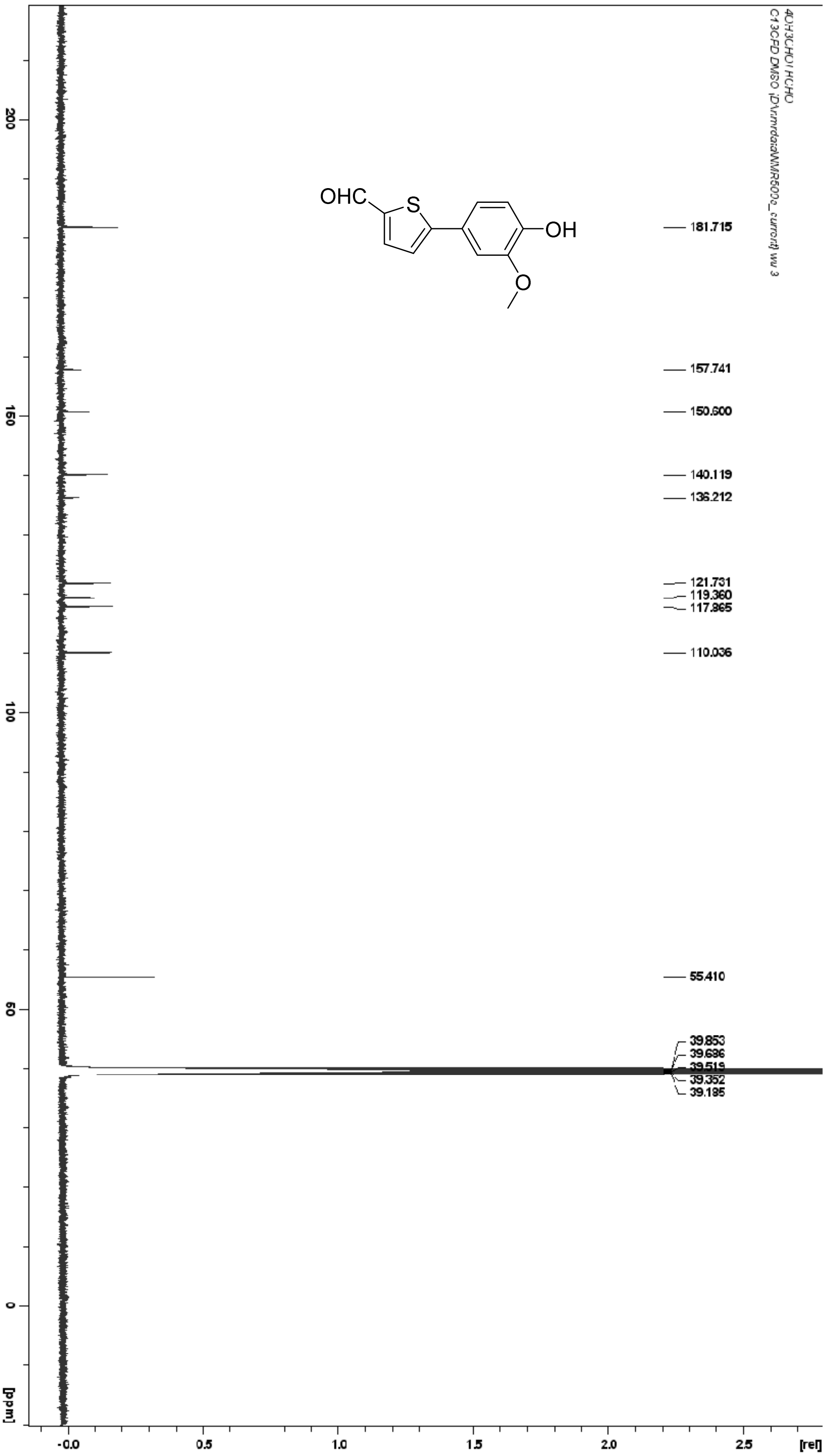
OHC
S
OH
O
181.715
157.741
150.600
140.119
136.212
121.731
119.360
117.865
110.036
55.410
39.853
39.686
39.519
39.352
39.185
200
150
100
50
0
[ppm]
-0.0
0.5
1.0
1.5
2.0
2.5
[rel]

$^{1}$H and $^{13}$C-NMR of ligand HS-350:

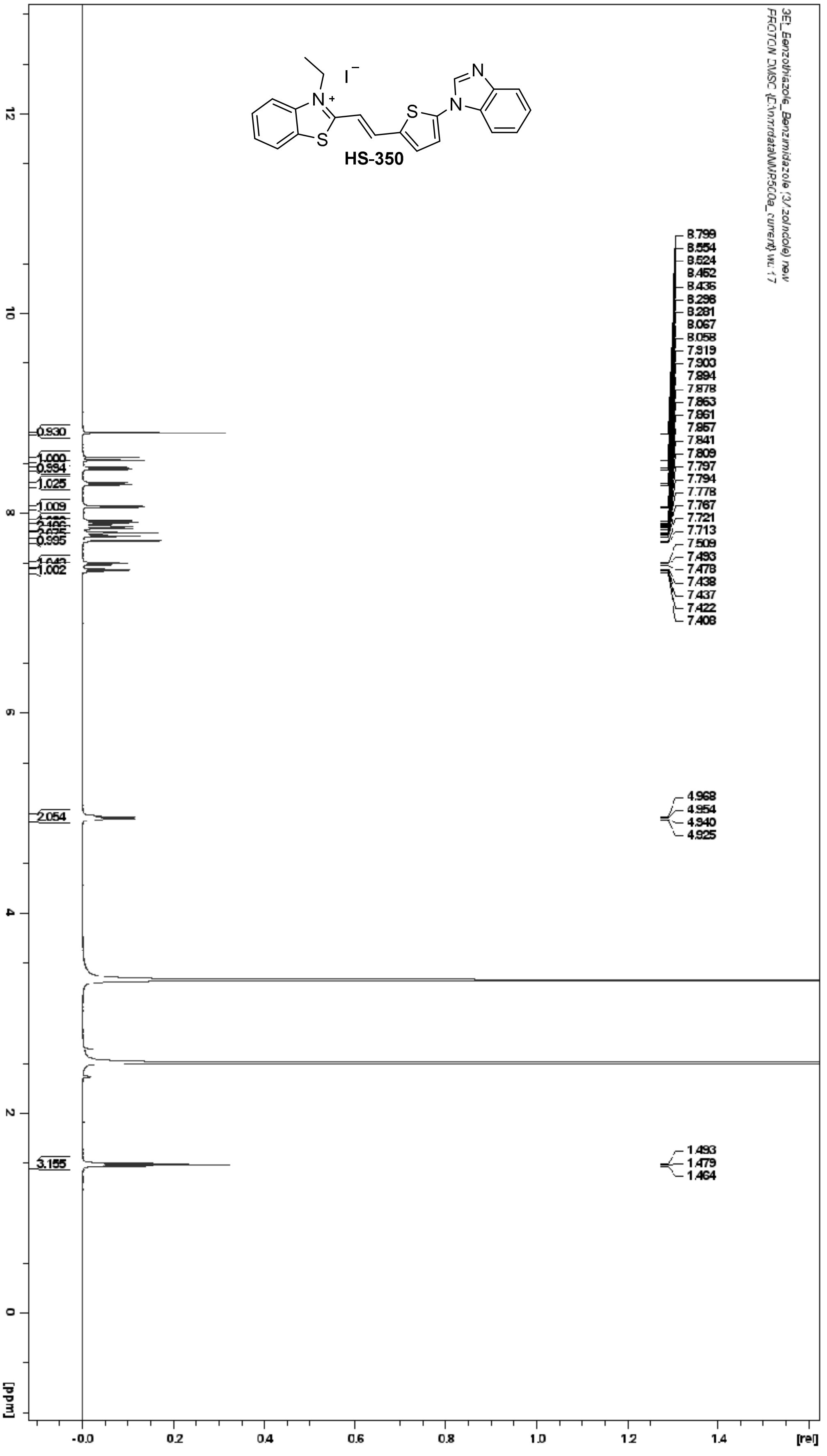

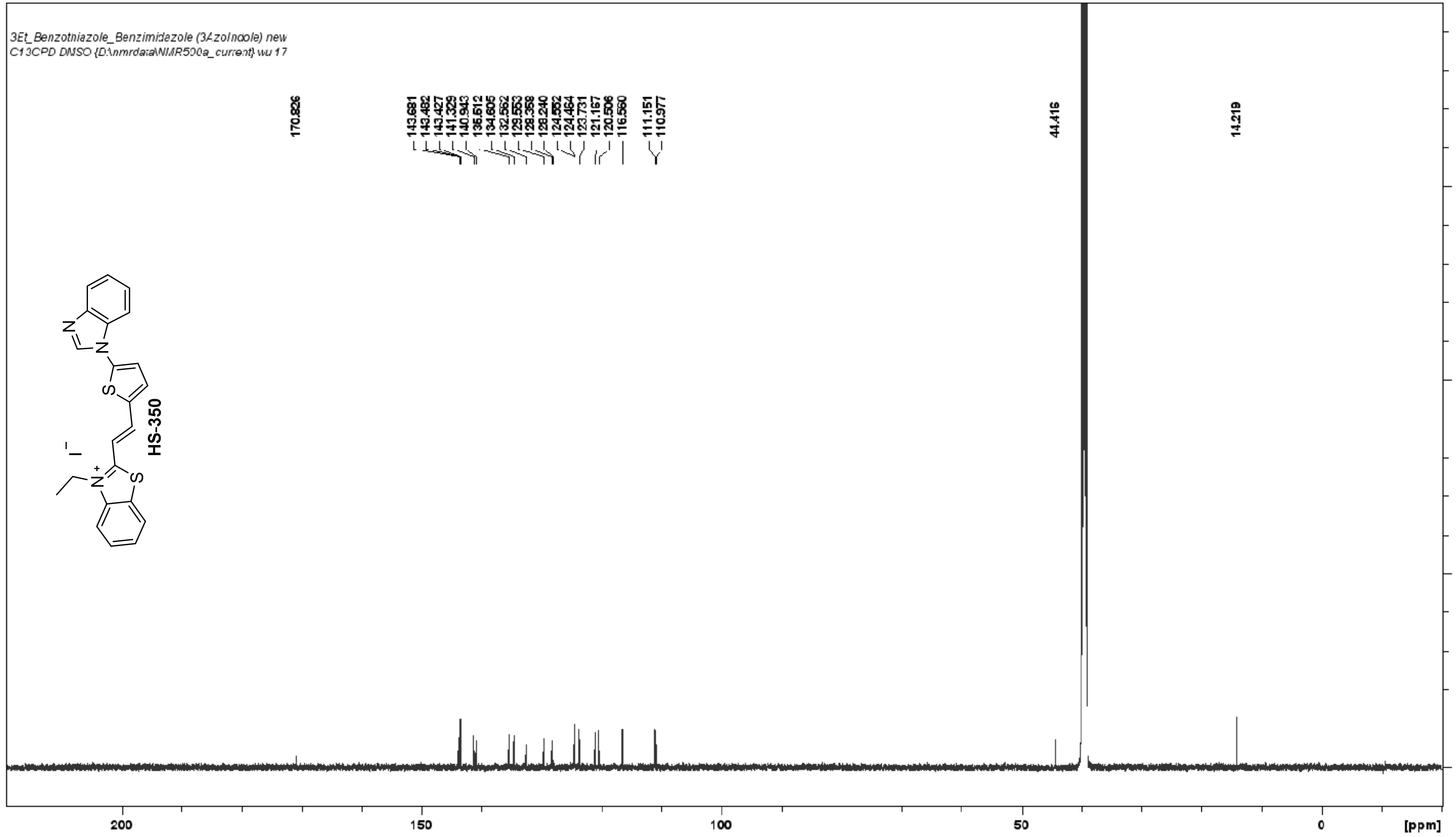
3Et_Benzothiazole_Benzimidazole (3AzoIndole) new
C13CPD DMSO {D:\nmrdata\NMR500a_current} wu 17
HS-350
I⁻
170.826
143.681
143.482
143.427
141.329
140.943
136.612
134.605
132.562
129.553
128.358
128.240
124.552
124.464
123.731
121.167
120.506
116.560
111.151
110.977
44.416
14.219
200
150
100
50
0
[ppm]
-0.0
0.5
1.0
1.5
[rel]

$^{1}H$ and $^{13}C$-NMR of ligand HS-351:

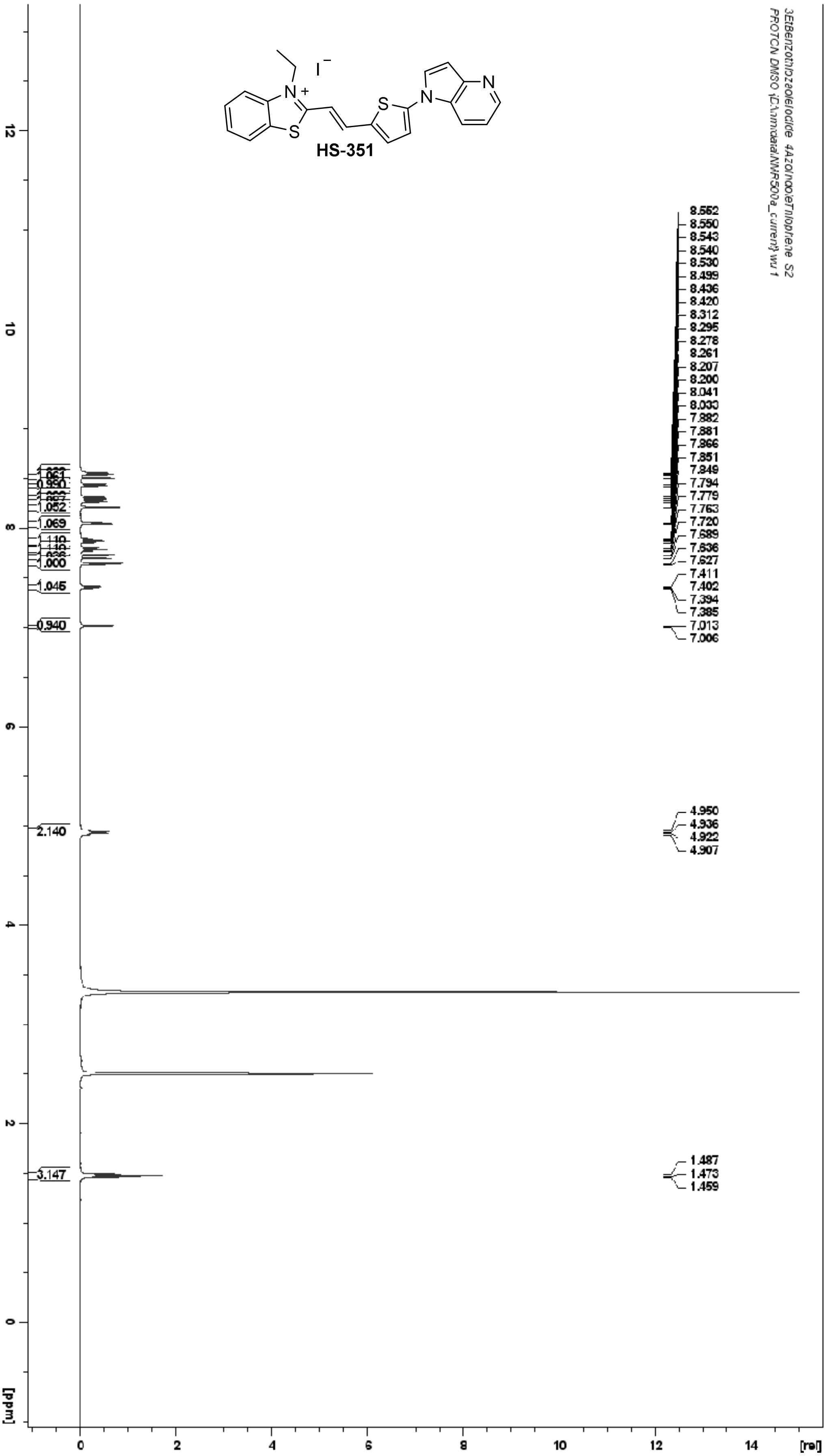

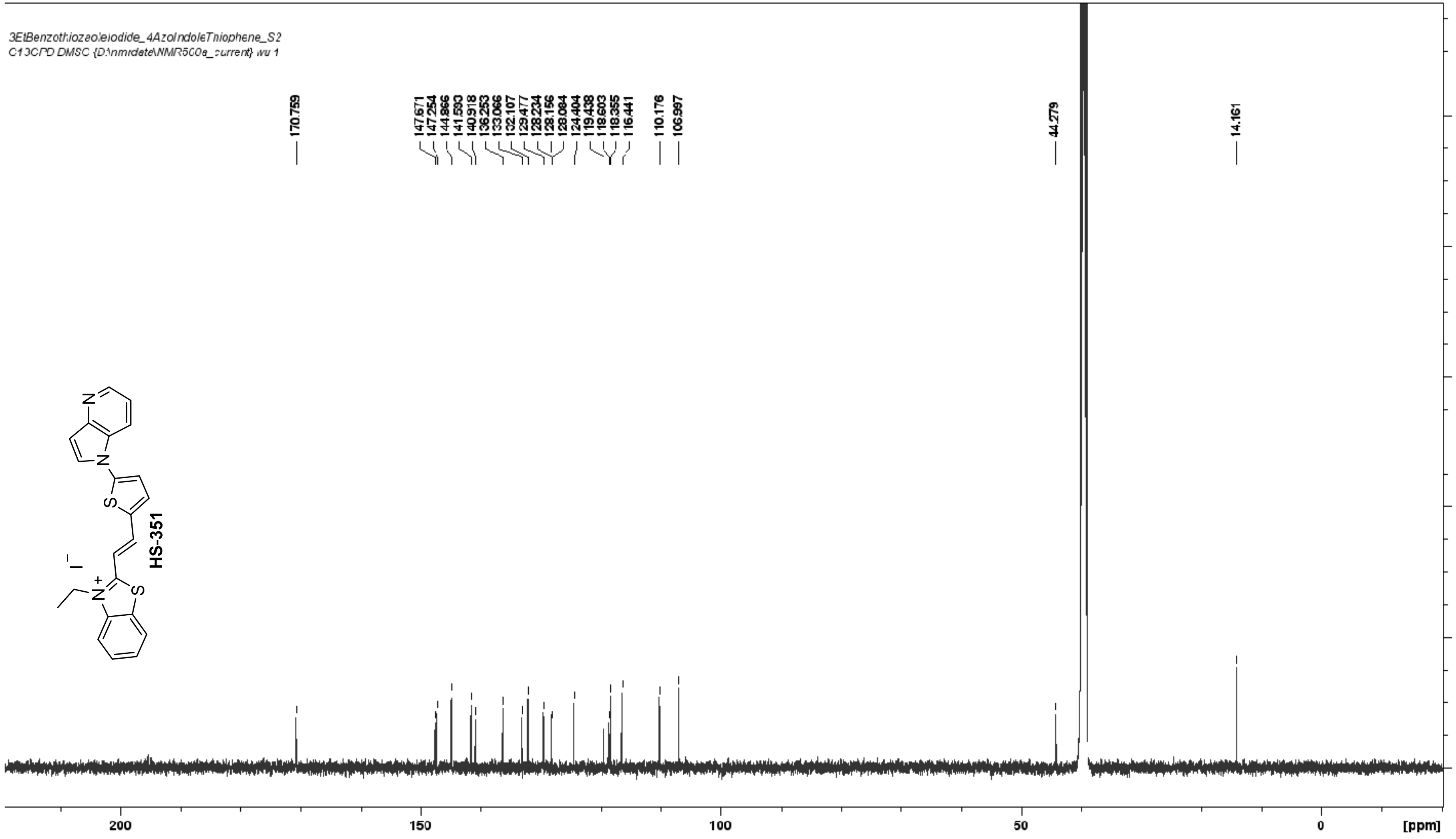
3EtBenzothiozeoleiodide_4AzoIndoleThiophene_S2
C13CPD DMSO {D:\nmrdate\NMR500a_current} wu 1
170.759
147.671
147.254
144.866
141.593
140.918
136.253
133.066
132.107
129.477
128.234
128.156
128.084
124.404
119.438
118.603
118.355
116.441
110.176
106.997
44.279
14.161
HS-351
I−
[rel]
[ppm]

$^{1}H$ and $^{13}C$-NMR of ligand HS-352:

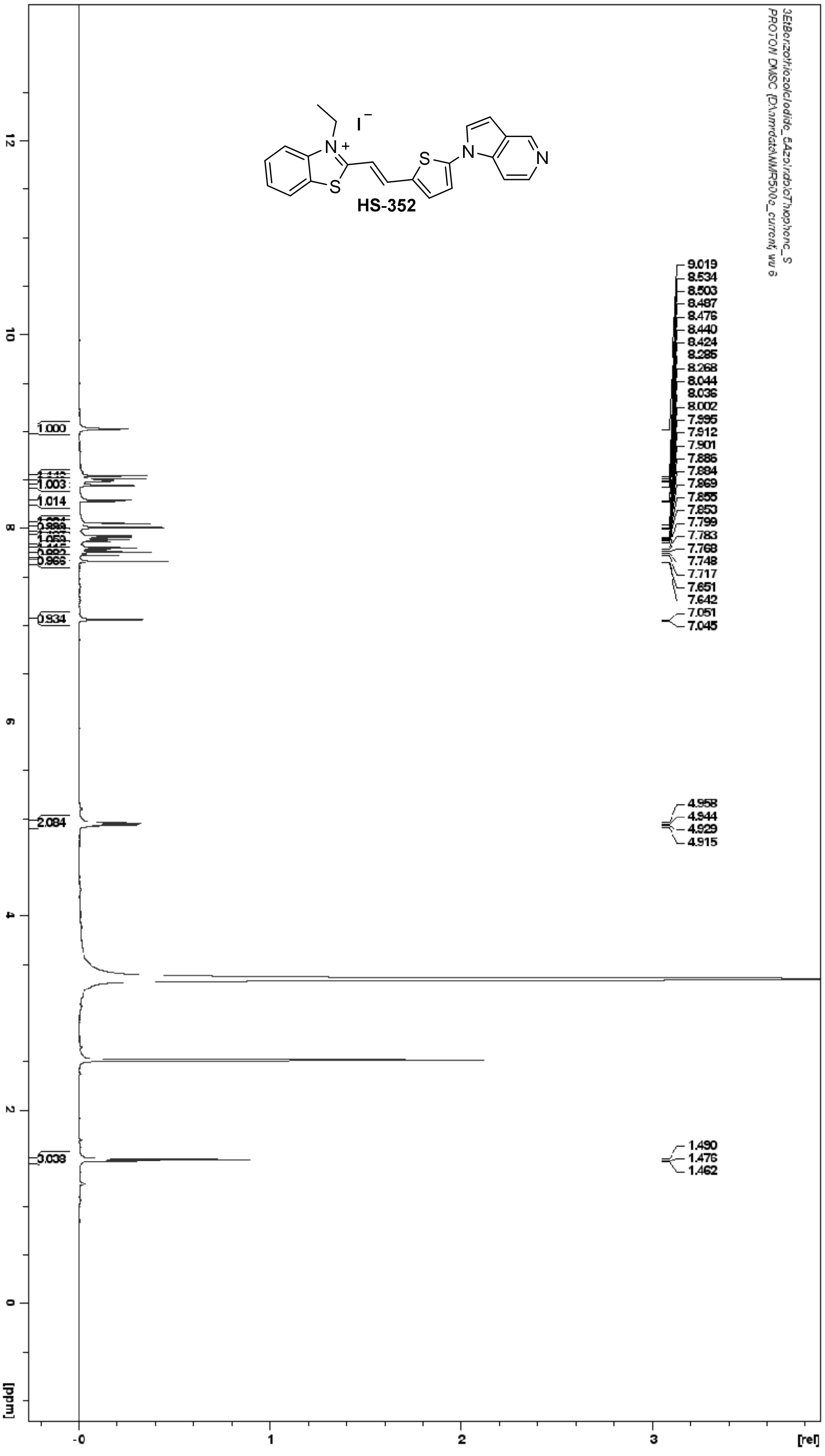

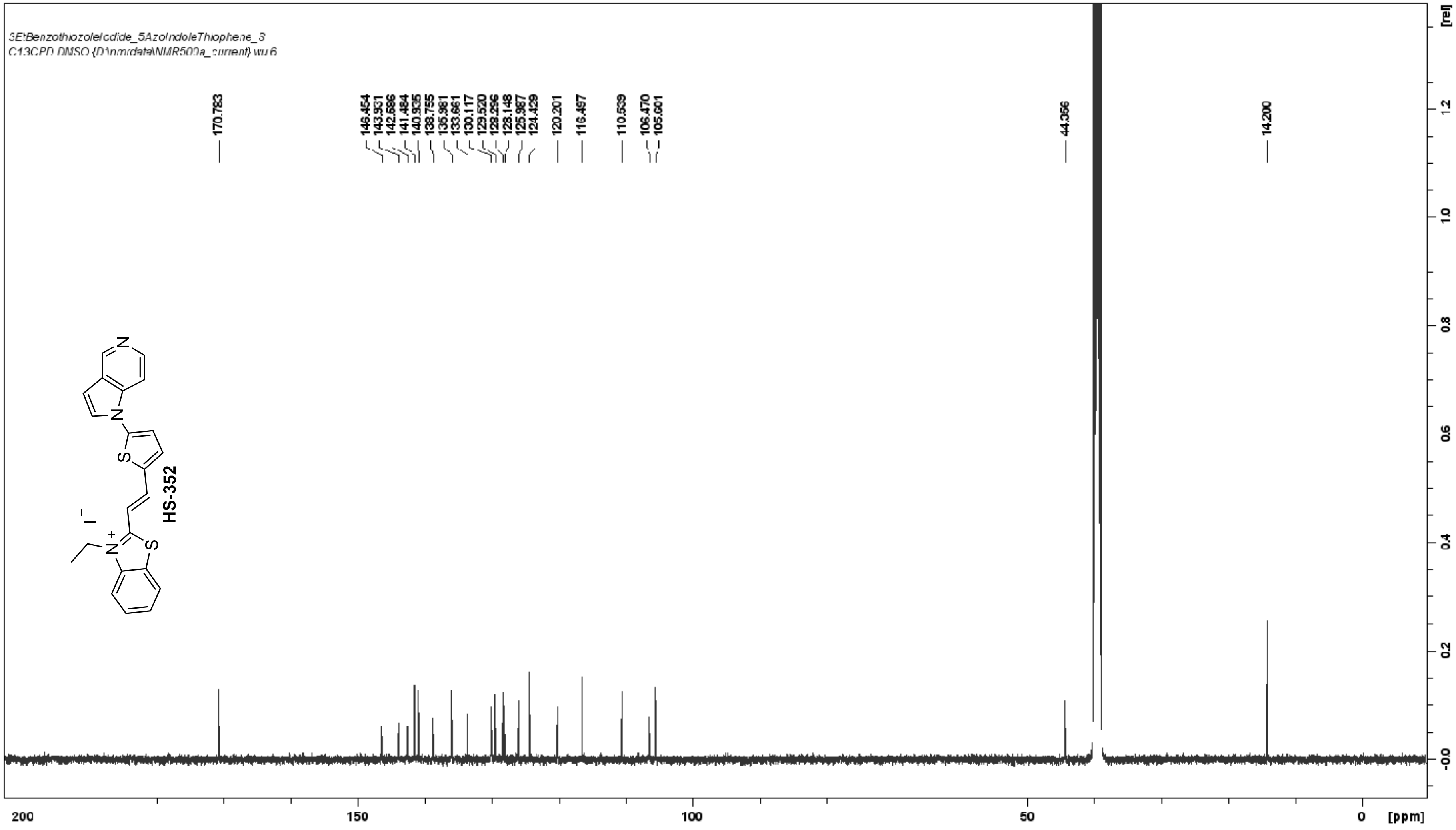
3E'Benzothiazoleiodide_5AzoIndoleThiophene_3
C13CPD DMSO {D:\nmrdata\NMR500a_current} wu 6
HS-352
I⁻
170.783
146.454
143.931
142.686
141.484
140.935
138.755
135.981
133.661
130.117
129.520
128.296
128.148
125.987
124.429
120.201
116.497
110.539
106.470
105.601
44.356
14.200
200
150
100
50
0
[ppm]
[rel]

$^{1}H$ and $^{13}C$-NMR of ligand HS-353:

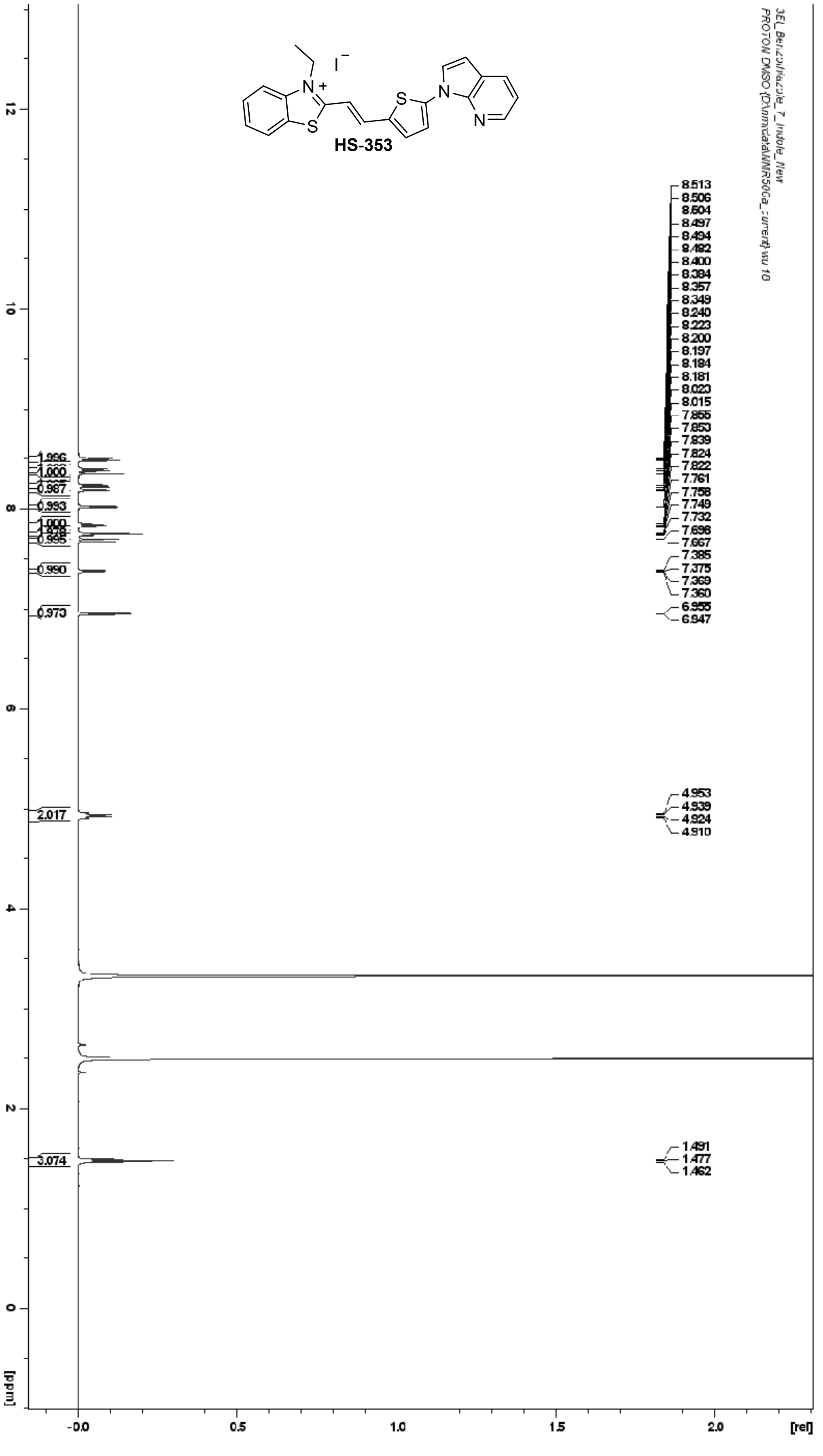

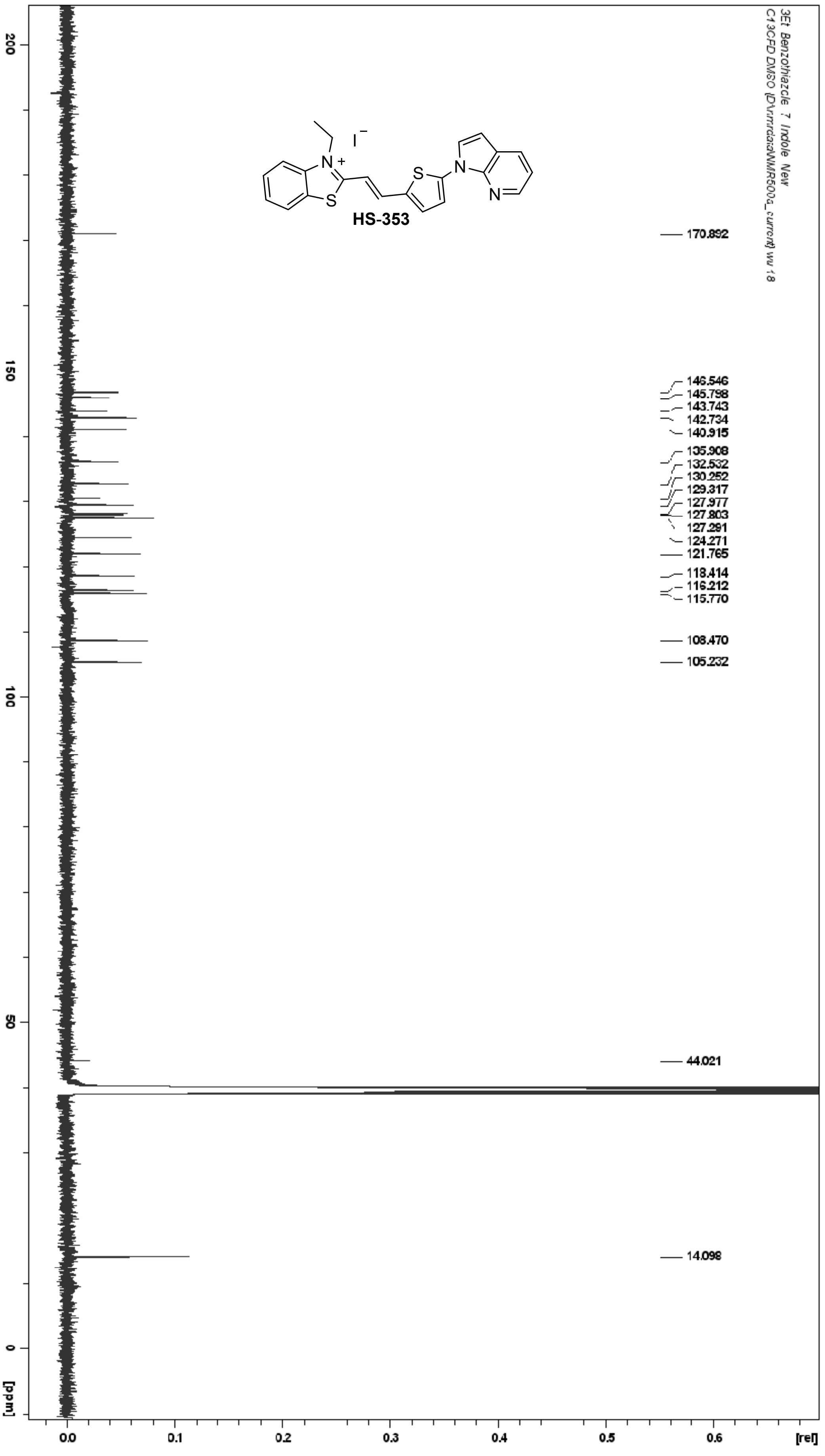

3Et Benzothiazole 7 Indole New
C13CPD DMSO {D:\nmrdata\NMR500_current} wu 18
I⁻
N+
S
N
N
HS-353
170.892
146.546
145.798
143.743
142.734
140.915
135.908
132.532
130.252
129.317
127.977
127.803
127.291
124.271
121.765
118.414
116.212
115.770
108.470
105.232
44.021
14.098
200
150
100
50
0
[ppm]
0.0
0.1
0.2
0.3
0.4
0.5
0.6
[rel]

$^{1}H$ and $^{13}C$-NMR of ligand HS-354:

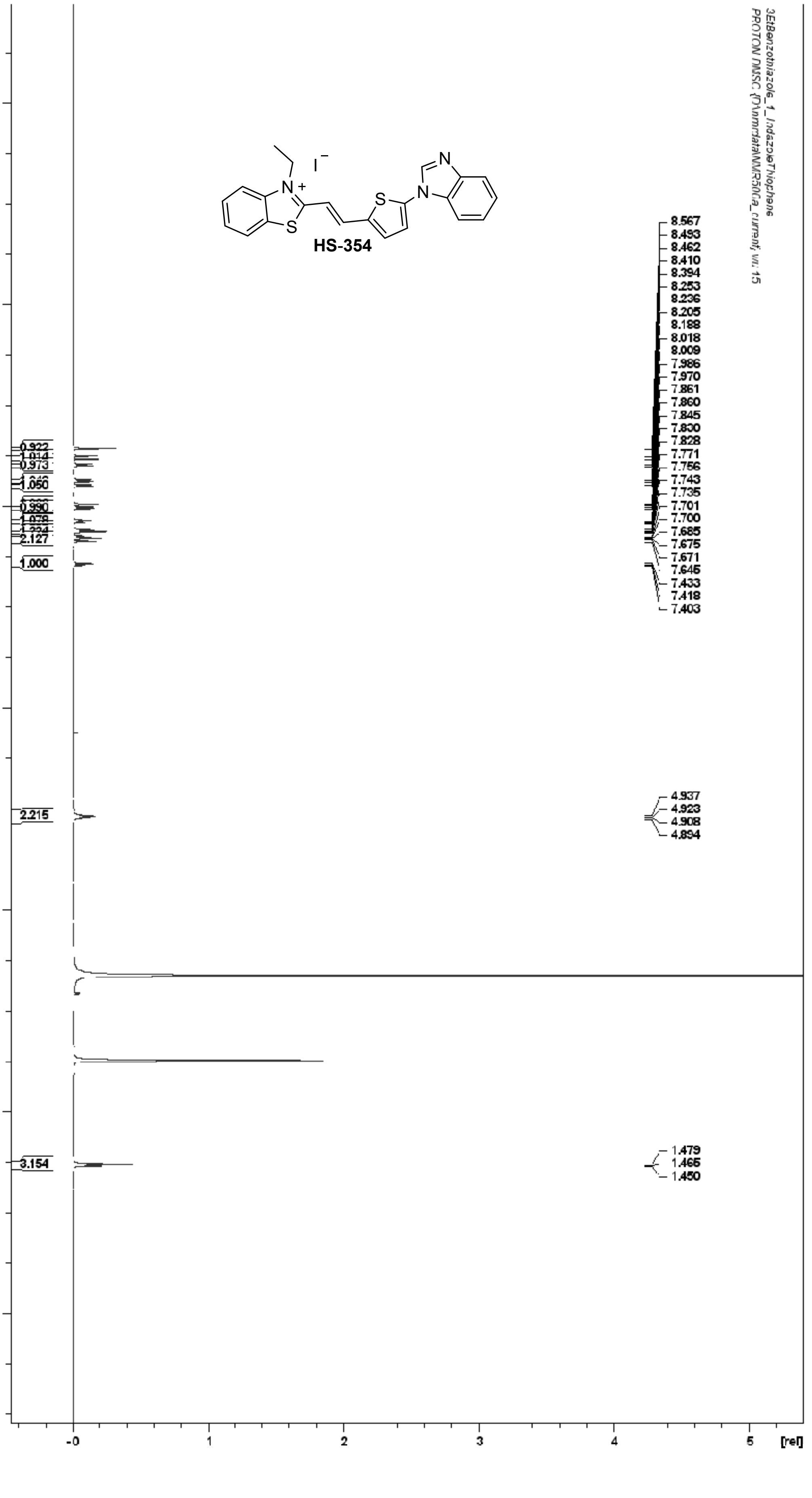

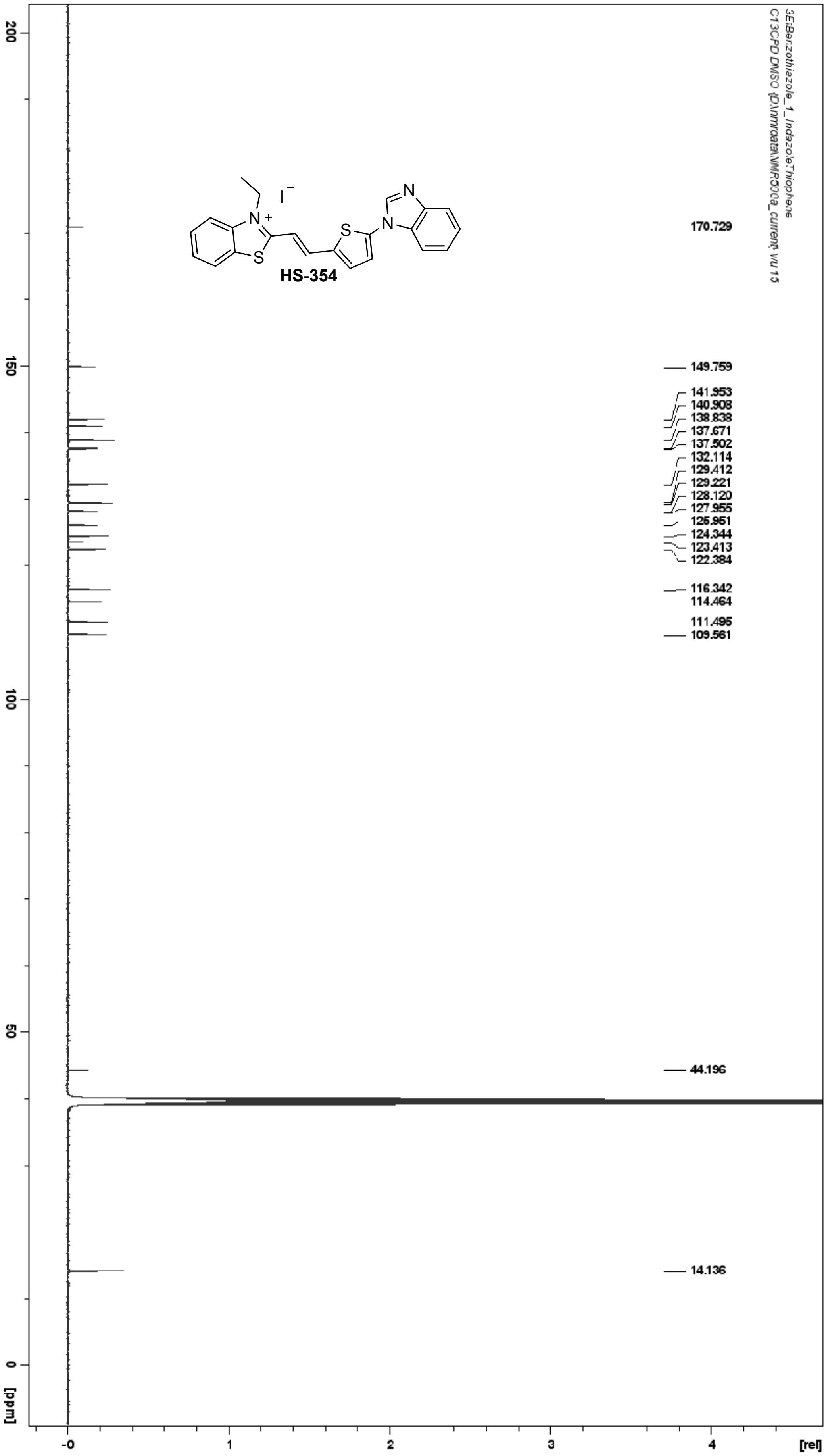
3EtBenzothiazole_1_IndazoleThiophene
C13CPD DMSO {D:\nmrdata\NMR500a_current} vu 15
I⁻
N+
S
S
N
N
HS-354
170.729
149.759
141.953
140.908
138.838
137.671
137.502
132.114
129.412
129.221
128.120
127.955
125.951
124.344
123.413
122.384
116.342
114.464
111.495
109.561
44.196
14.136
200
150
100
50
0
[ppm]
-0
1
2
3
4
[rel]

$^{1}H$ and $^{13}C$-NMR of ligand HS-355:

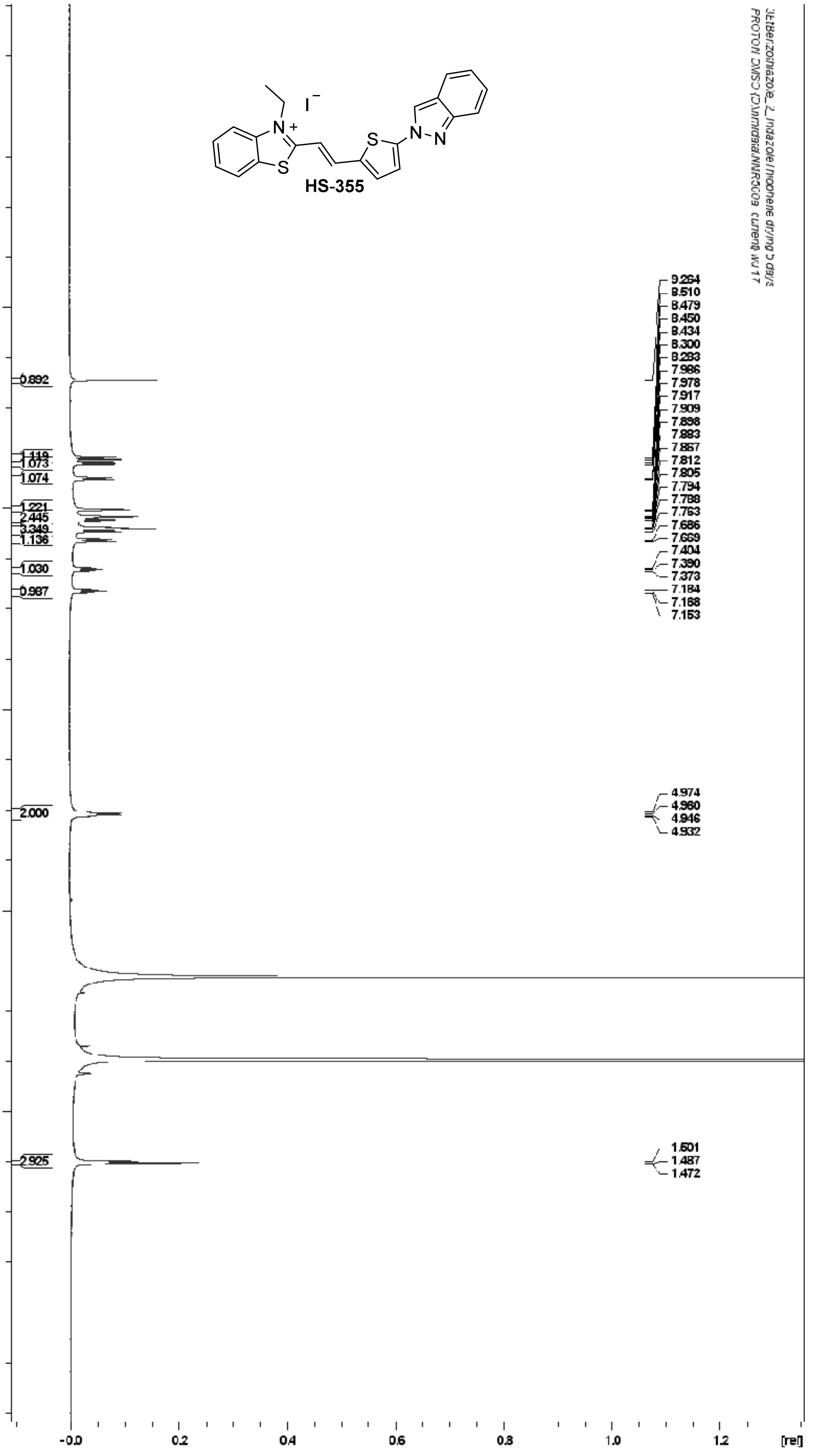

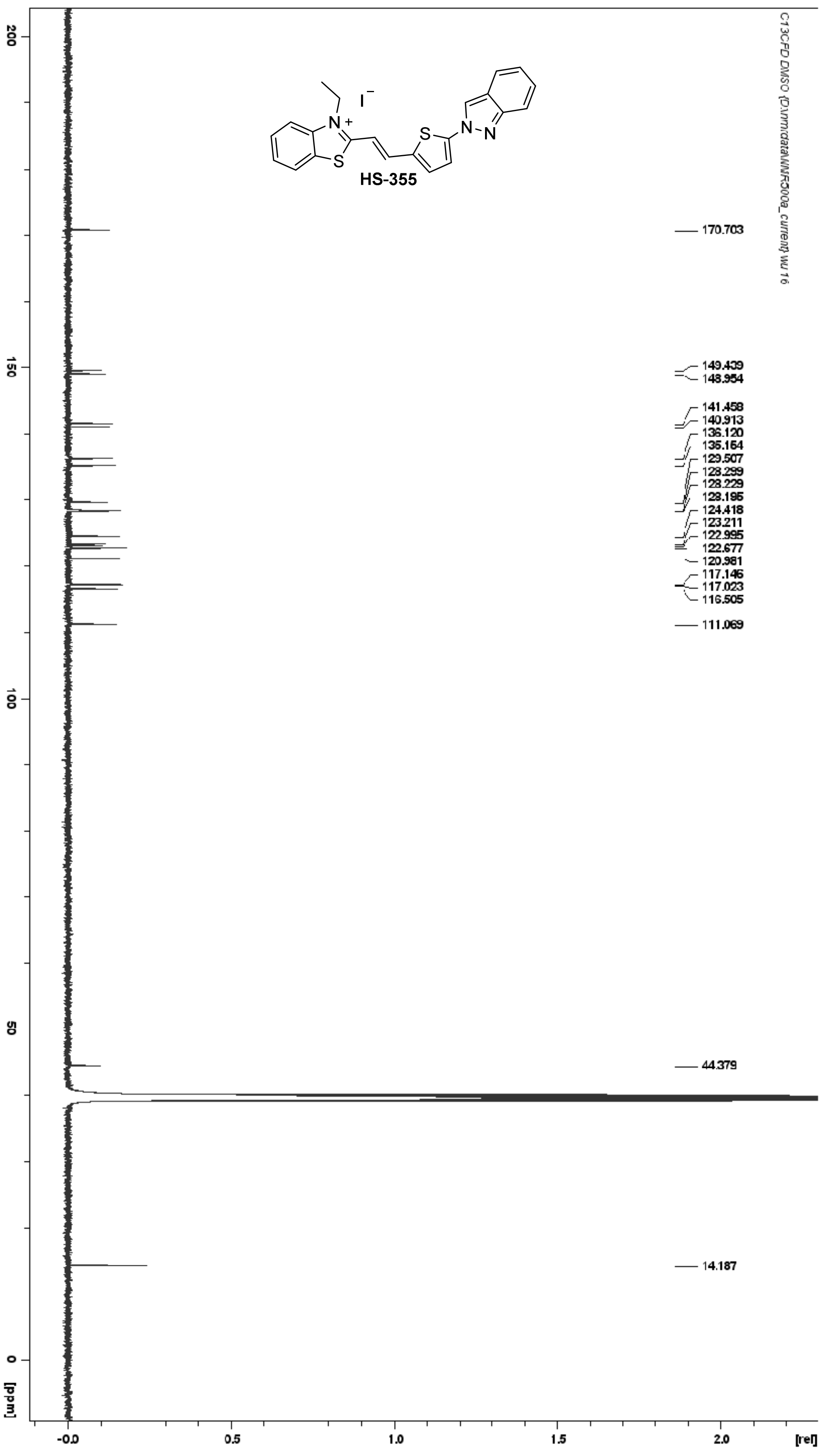

HS-355
I⁻
170.703
149.439
148.954
141.458
140.913
136.120
135.164
129.507
128.299
128.229
128.195
124.418
123.211
122.995
122.677
120.981
117.146
117.023
116.505
111.069
44.379
14.187
[ppm]
[rel]

$^1$H and $^{13}$C-NMR of ligand HS-356:

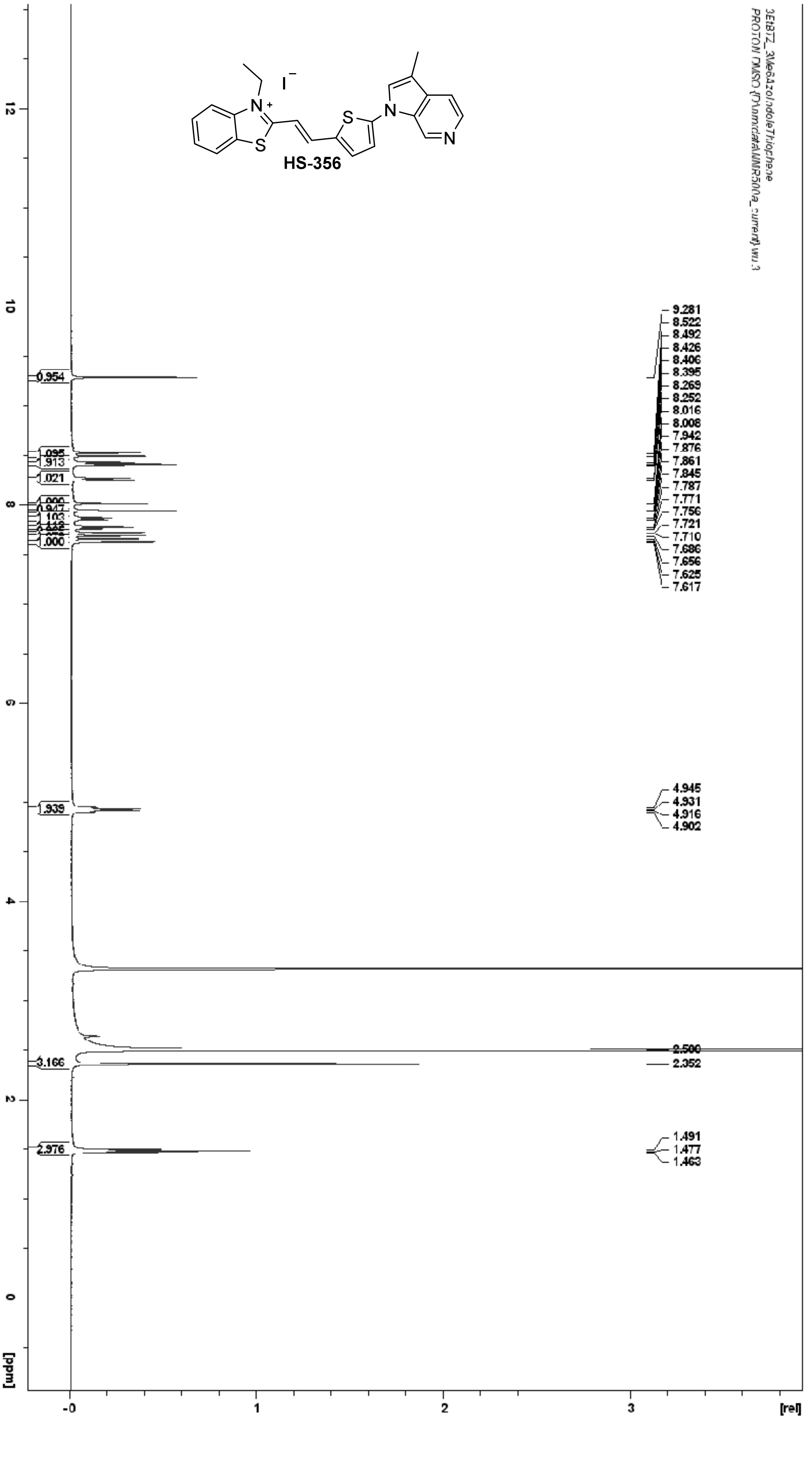

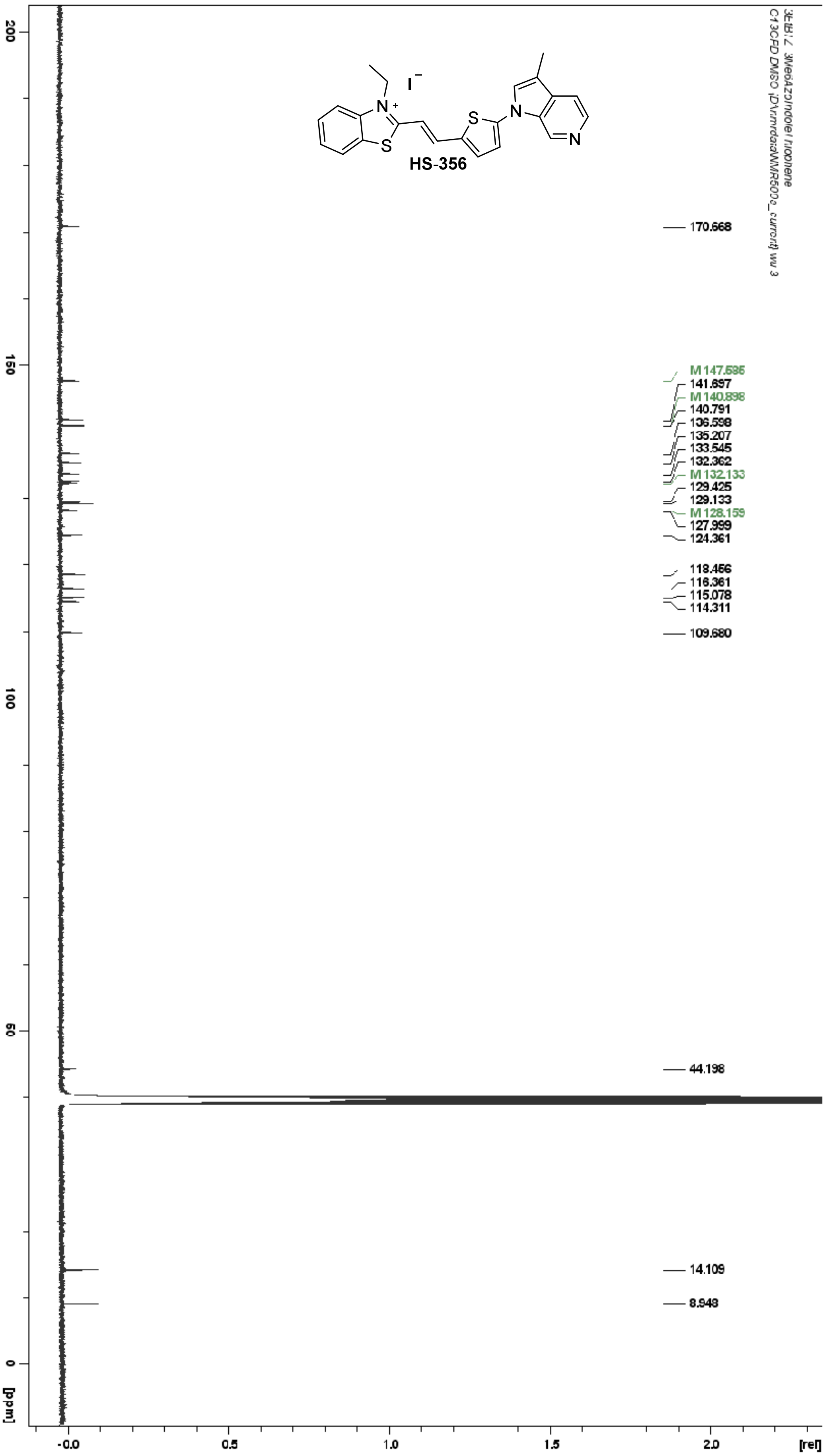

HS-356
I⁻
170.668
M 147.686
141.697
M 140.898
140.791
136.598
135.207
133.545
132.362
M 132.133
129.425
129.133
M 128.159
127.999
124.361
118.456
116.361
115.078
114.311
109.680
44.198
14.109
8.948
200
150
100
50
0
[ppm]
-0.0
0.5
1.0
1.5
2.0
[rel]

$^1$H and $^{13}$C-NMR of ligand HS-357:

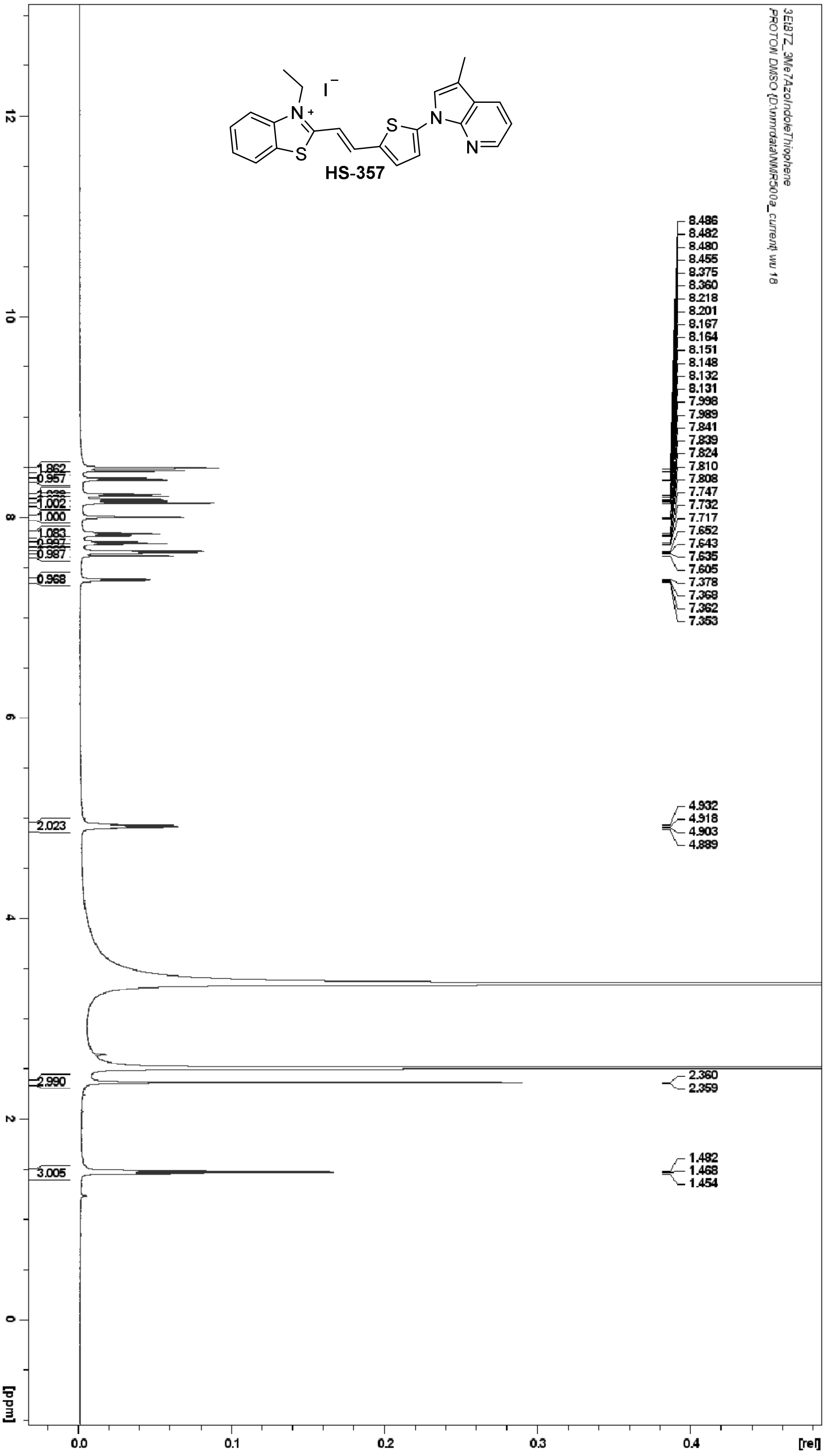

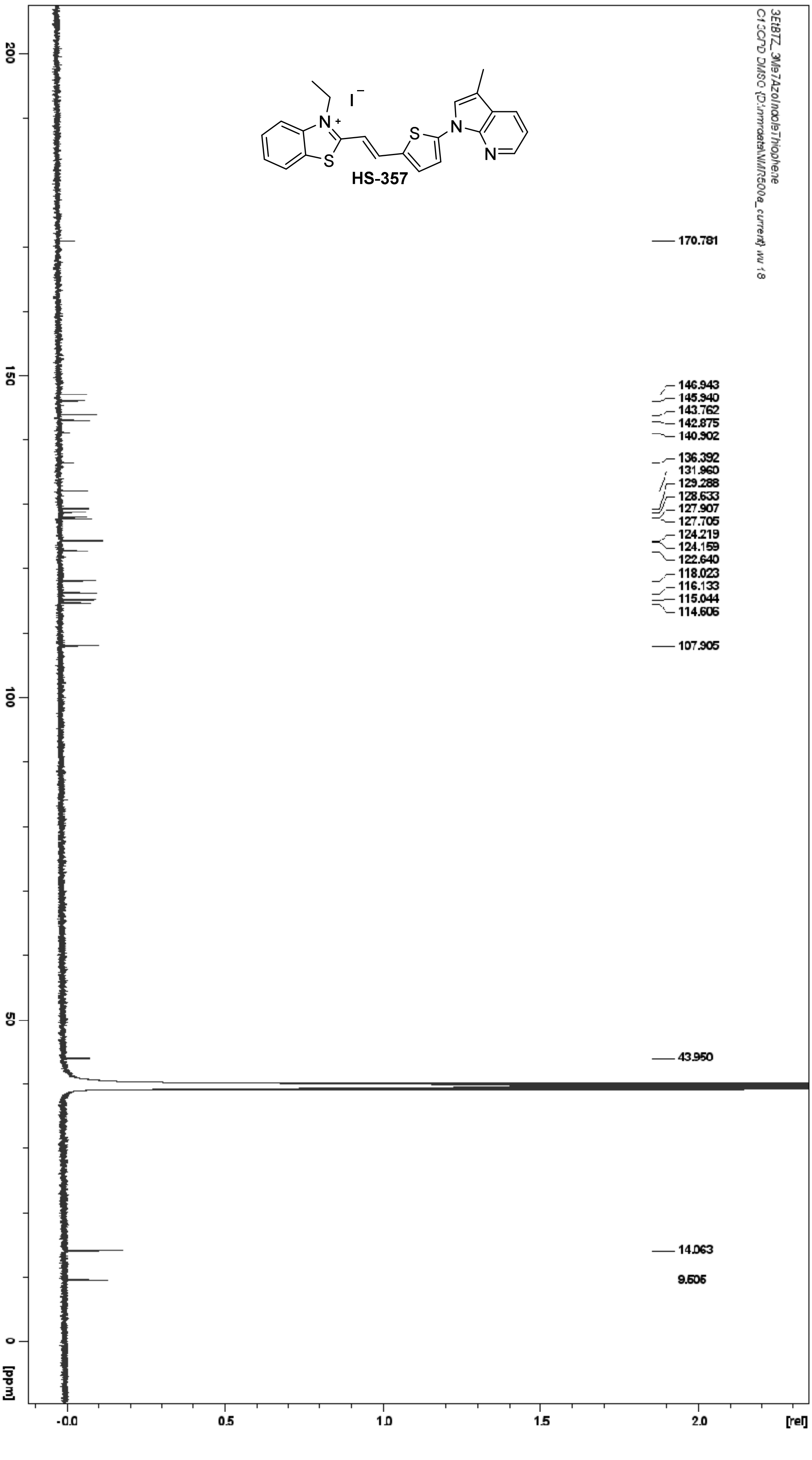
3EtBTZ_3Me7AzoIndolThiophene
C13CPD DMSO {D:\nmrdata\NMR500a_current} wu 18
HS-357
I⁻
170.781
146.943
145.940
143.762
142.875
140.902
136.392
131.960
129.288
128.633
127.907
127.705
124.219
124.159
122.640
118.023
116.133
115.044
114.606
107.905
43.950
14.063
9.505
[ppm]
[rel]

$^{1}H$ and $^{13}C$-NMR of ligand HS-358:

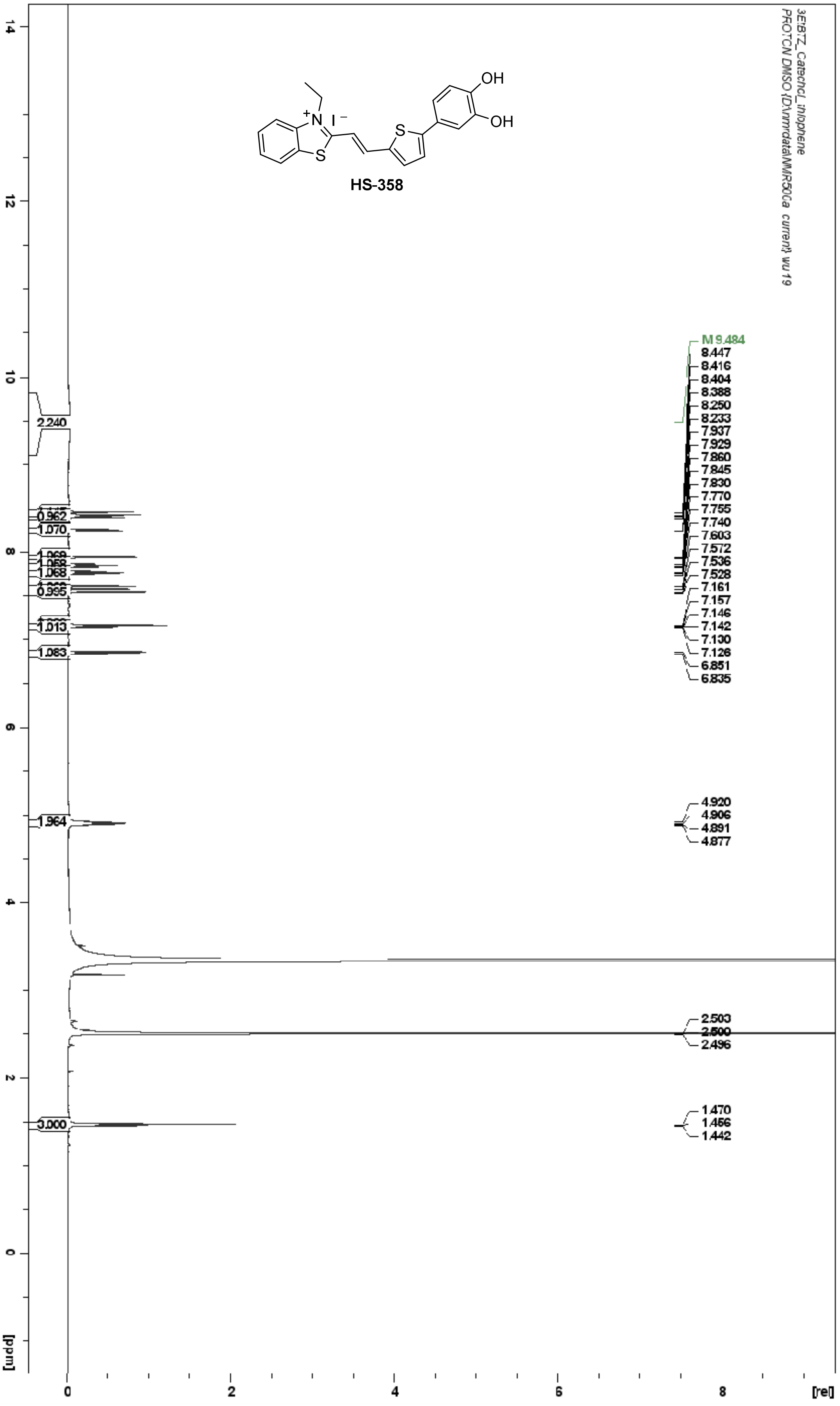

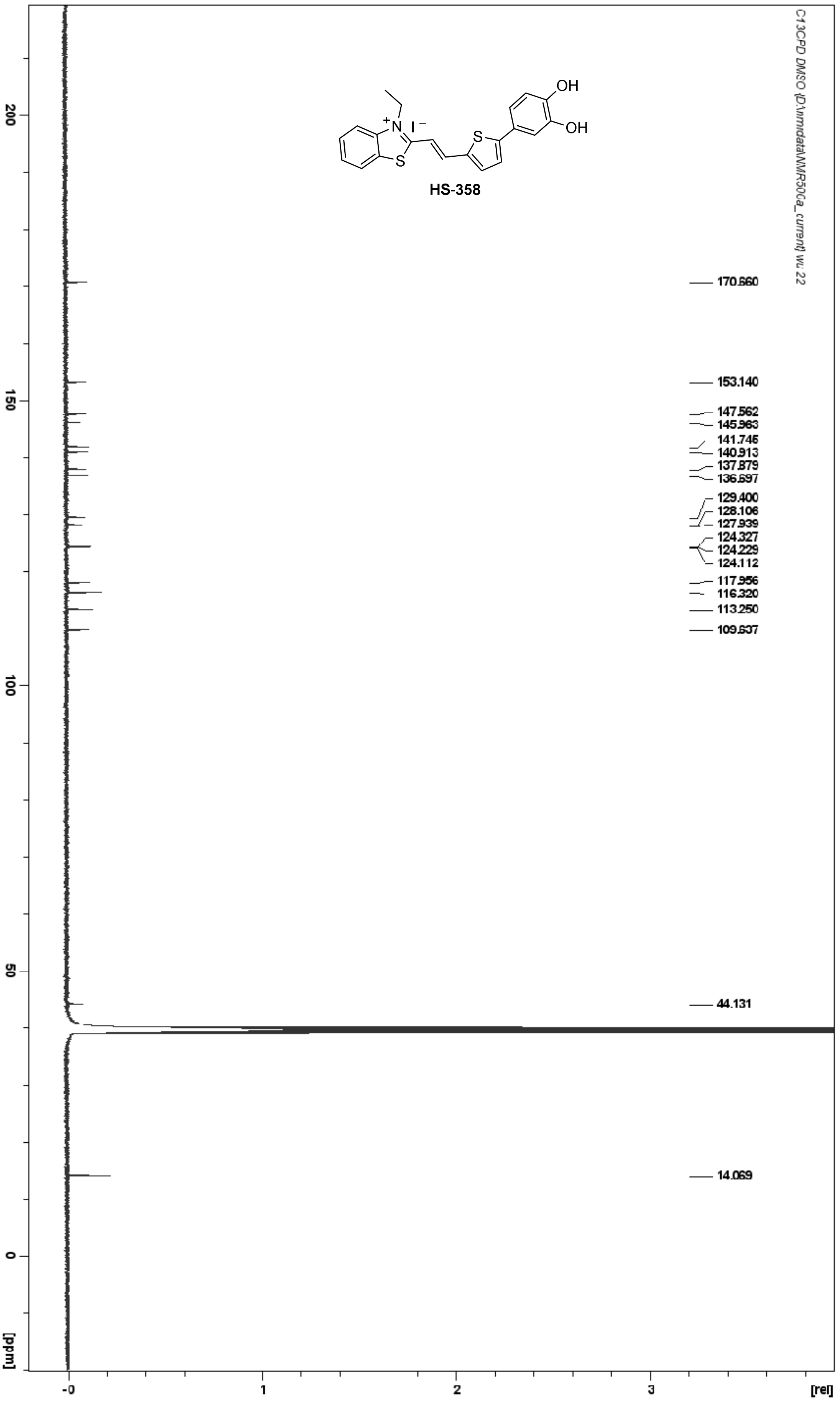

HS-358
C13CPD DMSO {D:\nmrdata\NMR50Ga_current} wu 22
170.660
153.140
147.562
145.963
141.745
140.913
137.879
136.697
129.400
128.106
127.939
124.327
124.229
124.112
117.956
116.320
113.250
109.637
44.131
14.069
[ppm]
[rel]

$^{1}H$ and $^{13}C$-NMR of ligand HS-359:

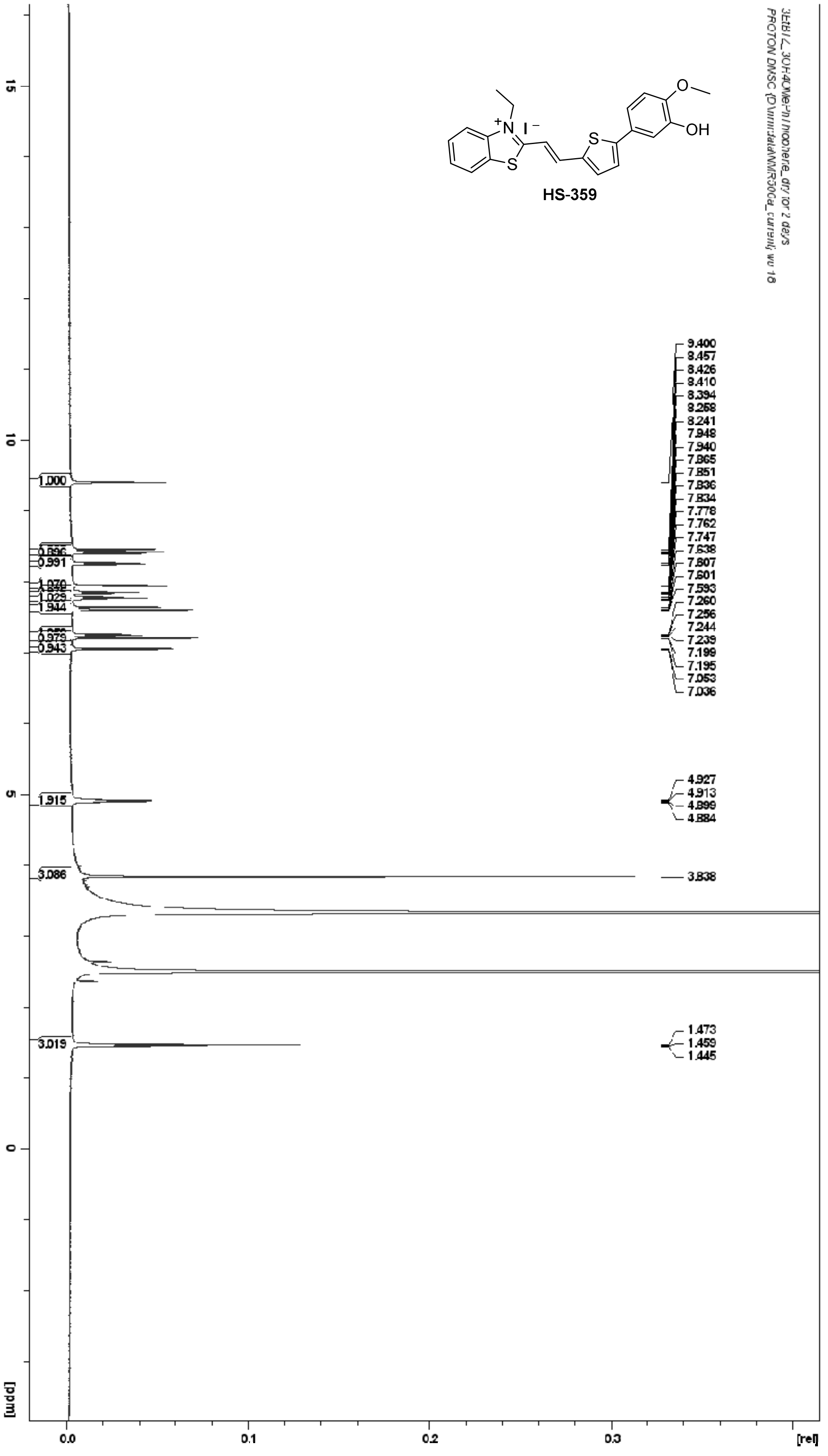

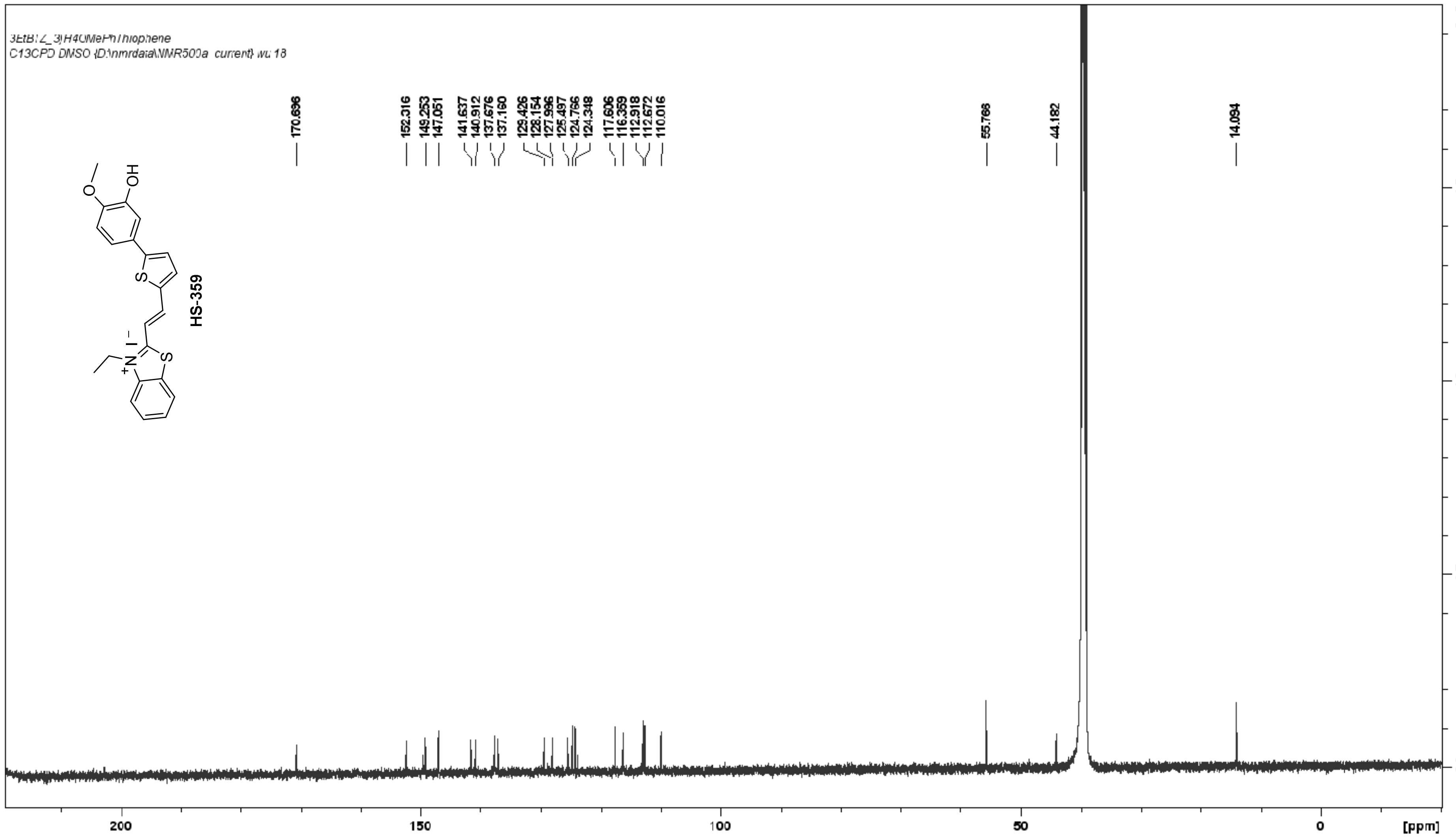
3EtBTZ_3)H4OMePhThiophene
C13CPD DMSO {D:\nmrdata\NMR500a current} wu 18
HS-359
S
OH
O
N
I
170.696
152.316
149.253
147.051
141.637
140.912
137.676
137.160
129.426
128.154
127.996
125.497
124.766
124.348
117.606
116.359
112.918
112.672
110.016
55.766
44.182
14.094
200
150
100
50
0
[ppm]
[rel]
-0.0
0.5
1.0
1.5

$^{1}H$ and $^{13}C$-NMR of ligand HS-360:

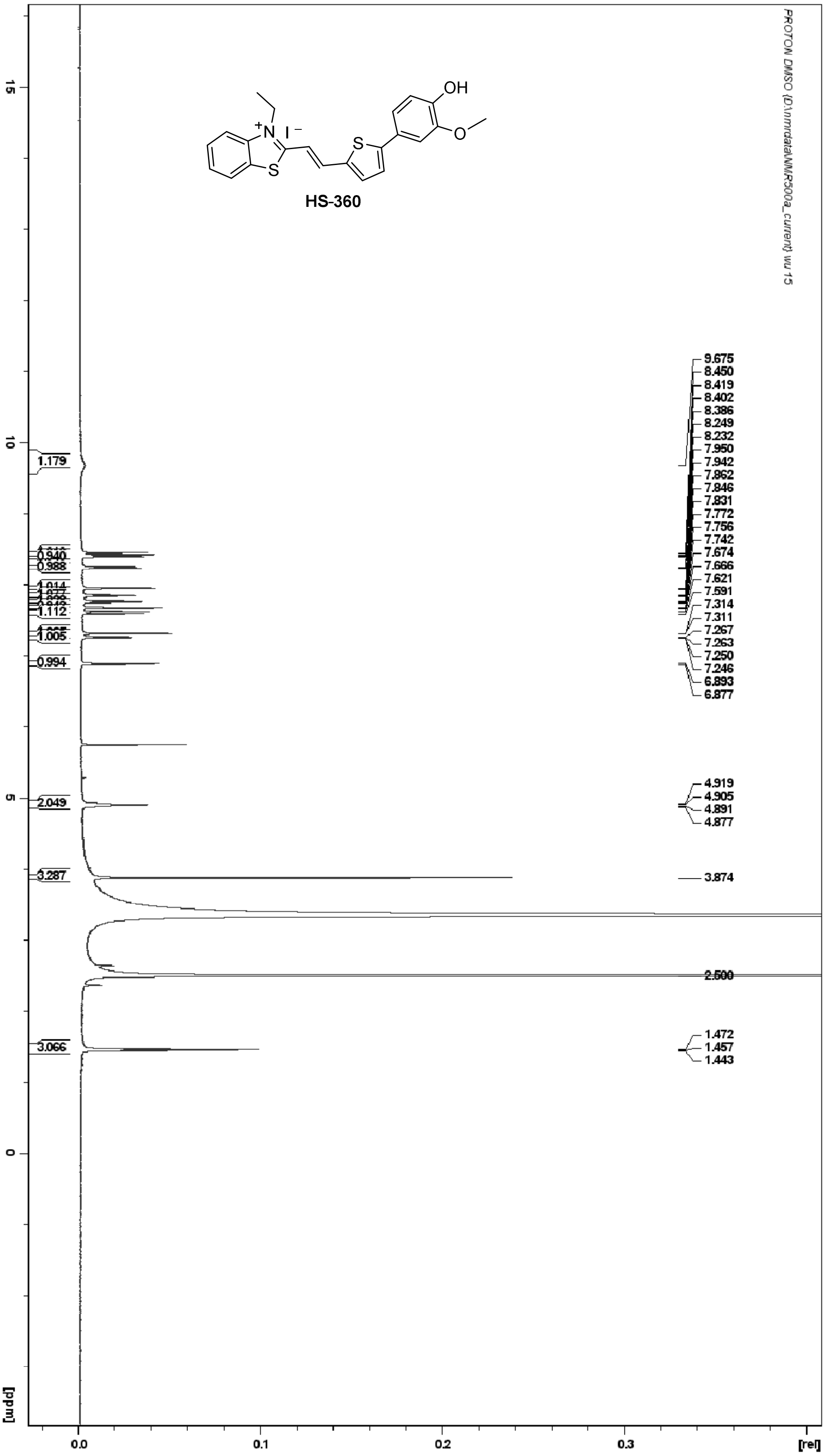

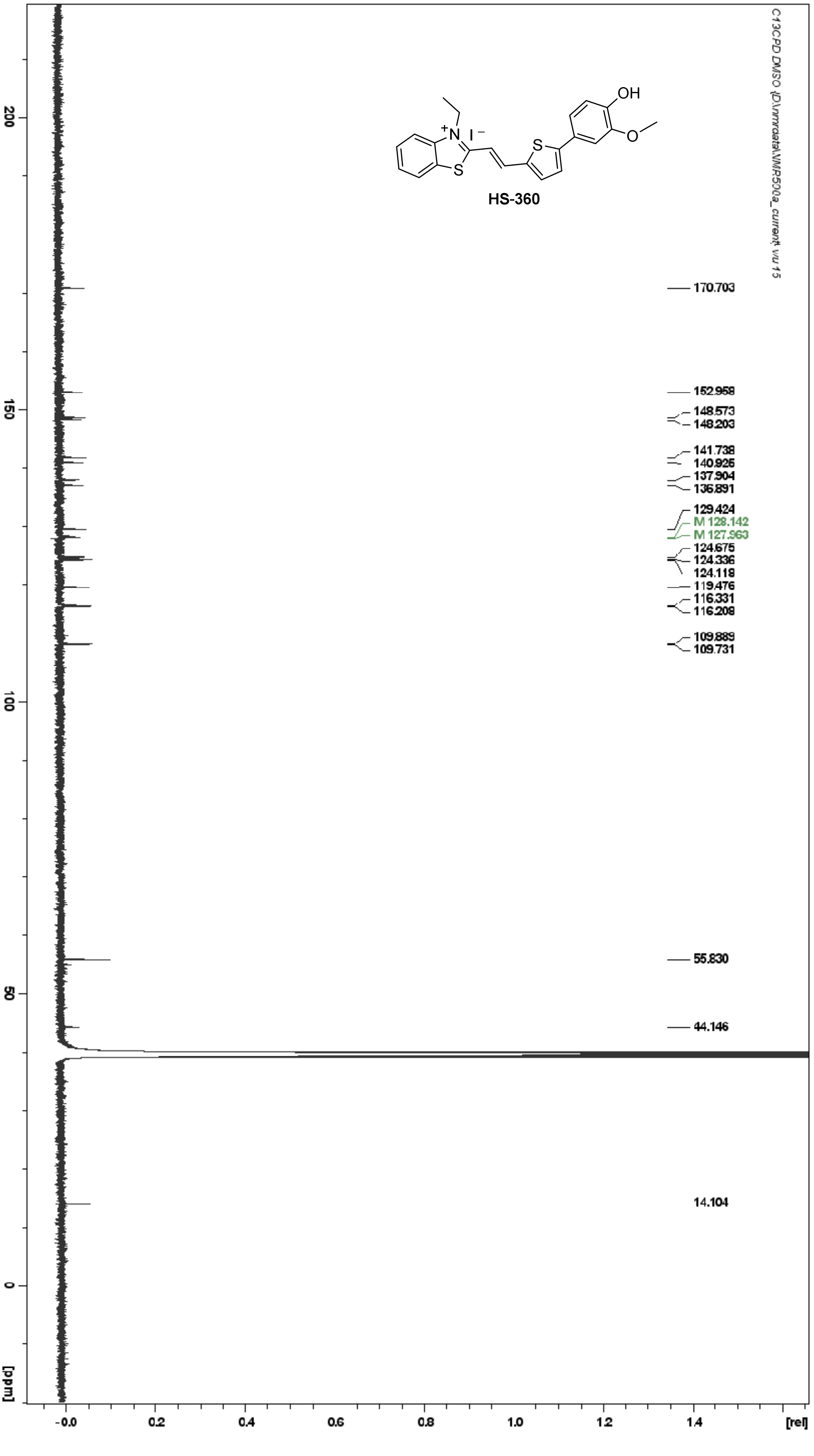

HS-360
C13CPD DMSO {D:\nmrdata\NMR500a_current} vu 15
170.703
152.958
148.573
148.203
141.738
140.925
137.904
136.891
129.424
M 128.142
M 127.963
124.675
124.336
124.118
119.476
116.331
116.208
109.889
109.731
55.830
44.146
14.104
200
150
100
50
0
[ppm]
-0.0
0.2
0.4
0.6
0.8
1.0
1.2
1.4
[rel]
